# Phylogenetics of Archerfishes (Toxotidae) and Evolution of the Toxotid Shooting Apparatus

**DOI:** 10.1093/iob/obac013

**Published:** 2022-03-21

**Authors:** M G Girard, M P Davis, Tan H.H., D J Wedd, P Chakrabarty, W B Ludt, A P Summers, W L Smith

**Affiliations:** Department of Ecology and Evolutionary Biology and Biodiversity Institute, University of Kansas, Lawrence, KS 66045, USA; Department of Vertebrate Zoology, Smithsonian National Museum of Natural History, Washington, DC 20560, USA; Department of Biological Sciences, St. Cloud State University, St. Cloud, MN 56301, USA; Lee Kong Chian Natural History Museum, National University of Singapore, 117377, SGP; Research Institute for the Environment and Livelihoods, Charles Darwin University, Darwin, NT 0810, AUS; Ichthyology Section, Museum of Natural Science, Department of Biological Sciences, Louisiana State University, Baton Rouge, LA 70803, USA; Department of Ichthyology, Natural History Museum of Los Angeles County, Los Angeles, CA 90007, USA; Department of Biology and SAFS, University of Washington's Friday Harbor Laboratories, Friday Harbor, WA 98250, USA; Department of Ecology and Evolutionary Biology and Biodiversity Institute, University of Kansas, Lawrence, KS 66045, USA

## Abstract

**Synopsis:**

Archerfishes (Toxotidae) are variously found in the fresh- and brackish-water environments of Asia Pacific and are well known for their ability to shoot water at terrestrial prey. These shots of water are intended to strike their prey and cause it to fall into the water for capture and consumption. While this behavior is well known, there are competing hypotheses (blowpipe vs. pressure tank hypothesis) of how archerfishes shoot and which oral structures are involved. Current understanding of archerfish shooting structures is largely based on two species, *Toxotes chatareus* and *T. jaculatrix*. We do not know if all archerfishes possess the same oral structures to shoot water, if anatomical variation is present within these oral structures, or how these features have evolved. Additionally, there is little information on the evolution of the Toxotidae as a whole, with all previous systematic works focusing on the interrelationships of the family. We first investigate the limits of archerfish species using new and previously published genetic data. Our analyses highlight that the current taxonomy of archerfishes does not conform to the relationships we recover. *Toxotes mekongensis* and *T. siamensis* are placed in the synonymy of *T. chatareus*, *Toxotes carpentariensis* is recognized as a species and removed from the synonymy of *T. chatareus*, and the genus *Protoxotes* is recognized for *T. lorentzi* based on the results of our analyses. We then take an integrative approach, using a combined analysis of discrete hard- and soft-tissue morphological characters with genetic data, to construct a phylogeny of the Toxotidae. Using the resulting phylogenetic hypothesis, we then characterize the evolutionary history and anatomical variation within the archerfishes. We discuss variation in the oral structures and the evolution of the mechanism with respect to the interrelationships of archerfishes, and find that the oral structures of archerfishes support the blowpipe hypothesis but soft-tissue oral structures may also play a role in shooting. Finally, by comparing the morphology of archerfishes to their sister group, we find that the Leptobramidae has relevant shooting features in the oral cavity, suggesting that some components of the archerfish shooting mechanism are examples of co-opted or exapted traits.

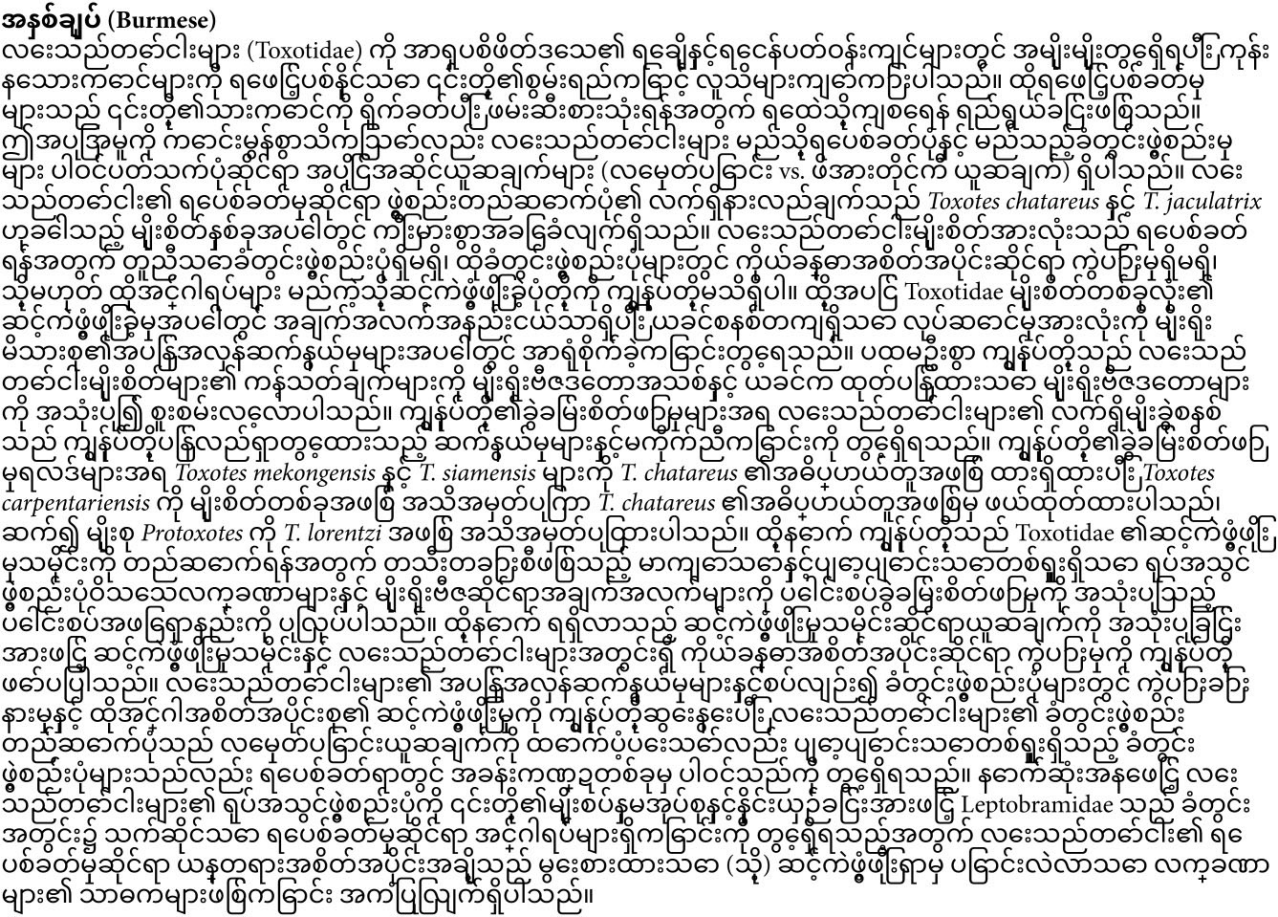

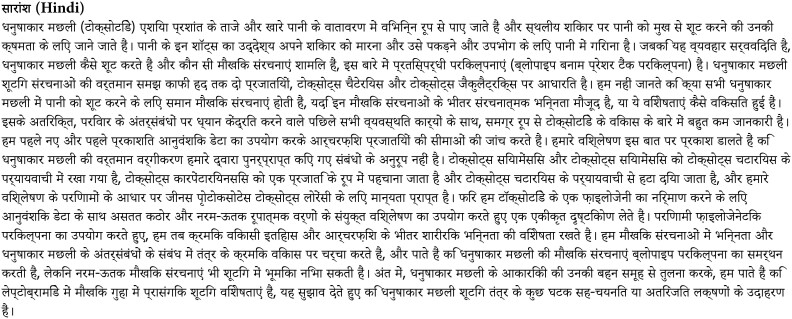

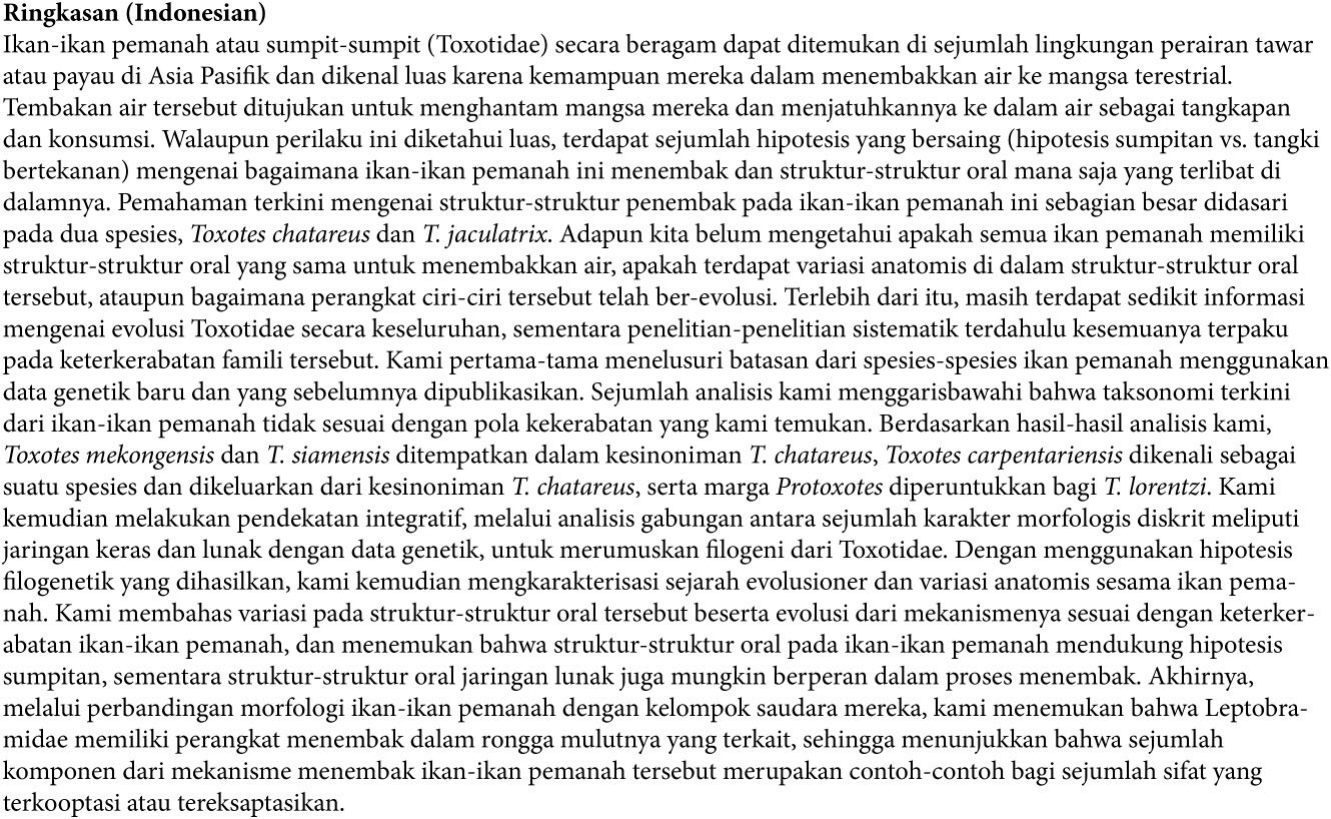

**Sinopsis (Malay):**

Pelbagai jenis Ikan Sumpit (Toxotidae) dapat dijumpai di persekitaran air tawar dan payau di Asia Pasifik dan mereka terkenal dengan kebolehan mereka menembak air ke arah mangsa di darat. Tembakan air ini bertujuan untuk menyerang mangsa mereka dan menyebabkan mereka jatuh ke dalam air untuk ditangkap dan dimakan. Walaupun tingkah laku ini diketahui umum, terdapat hipotesis yang bersaing (hipotesis sumpitan vs. tangki tekanan) tentang cara ikan sumpit menembak dan struktur mulut yang terlibat. Pemahaman semasa tentang struktur menembak ikan sumpit adalah sebahagian besarnya berdasarkan dua spesies, *Toxotes chatareus* dan *T. jaculatrix*. Kami tidak pasti sama ada semua ikan sumpit mempunyai struktur mulut yang sama untuk menembak air, jika variasi anatomi terdapat dalam struktur mulut ini, atau bagaimana ciri-ciri ini telah berkembang. Tambahan pula, terdapat sedikit maklumat tentang evolusi Toxotidae secara keseluruhan, dengan semua penyelidikan sistematik sebelum ini memfokuskan pada hubungan saling keluarga. Kami pada mulanya mengkaji had spesies ikan sumpit ini menggunakan data genetik baharu dan yang diterbitkan sebelum ini. Analisis kami menunjukkan bahawa taksonomi semasa ikan sumpit tidak mematuhi hubungan yang kami perolehi. *Toxotes mekongensis* dan *T. siamensis* diletakkan bersama kesinoniman *T. chatareus*, *Toxotes carpentariensis* yang diiktiraf sebagai satu spesies dan dikeluarkan daripada kesinoniman *T. chatareus*, dan genus *Protoxotes* yang diiktiraf untuk *T. lorentzi* adalah berdasarkan hasil analisis kami. Kemudian kami mengambil pendekatan integratif, menggunakan analisis gabungan karakter morfologi tisu keras dan lembut diskret dengan data genetik, untuk membina filogeni Toxotidae. Menggunakan hipotesis filogenetik yang terhasil, kami kemudian mencirikan sejarah evolusi dan variasi anatomi dalam ikan sumpit. Kami membincangkan variasi dalam struktur mulut dan evolusi mekanisme berkenaan yang berkaitan dengan ikan sumpit, dan mendapati bahawa struktur mulut ikan sumpit menyokong hipotesis sumpitan tetapi struktur mulut tisu lembut juga mungkin memainkan peranan dalam cara menembak. Akhir sekali, dengan membandingkan morfologi ikan sumpit kepada kumpulan saudara mereka, kami mendapati bahawa Leptobramidae mempunyai ciri penangkapan yang relevan dalam rongga mulut mereka, menunjukkan bahawa beberapa komponen mekanisme penangkapan ikan sumpit merupakan contoh ciri-ciri yang diikut-sertakan atau diguna semula.

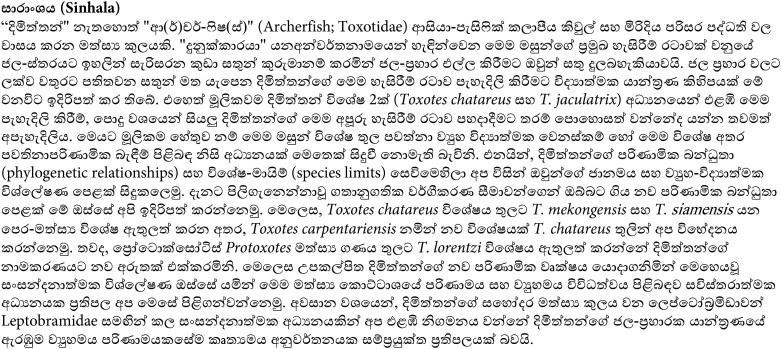

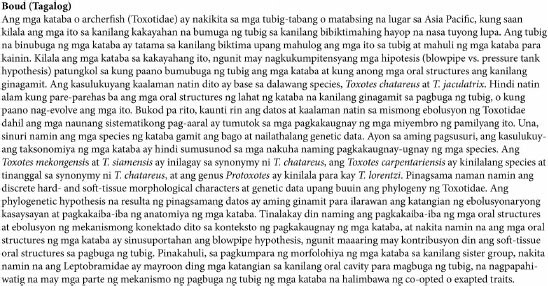

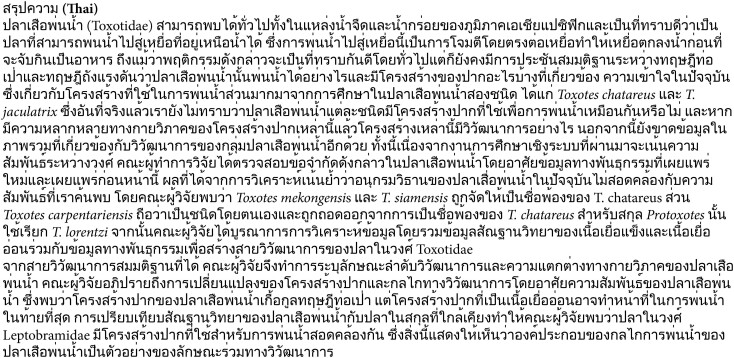

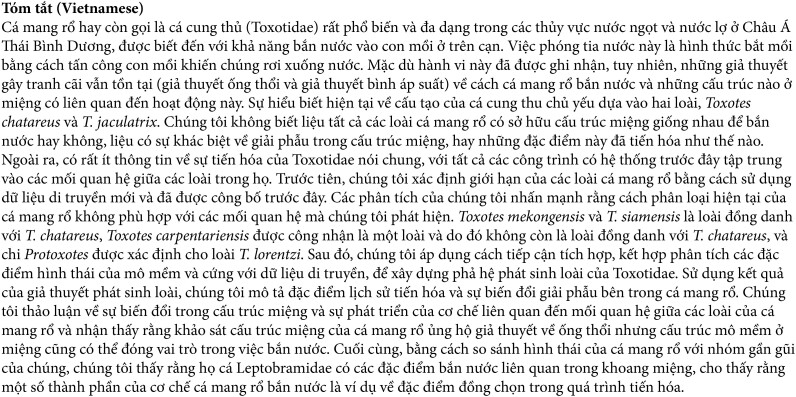

## Introduction

**Fig. 1:**
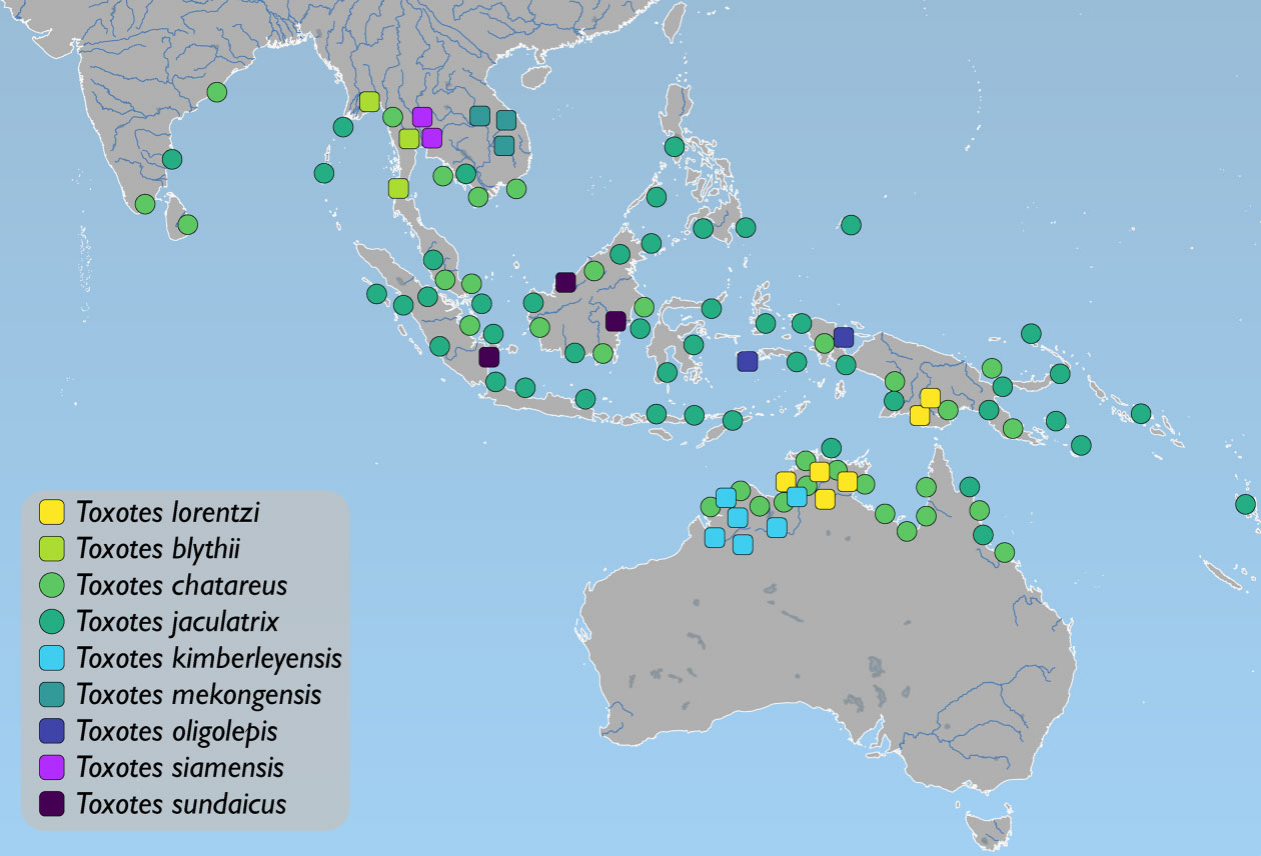
Distribution of archerfishes (Toxotidae) across the Indo-West Pacific based on Allen (1978, fig. 1) with modification. Map generated in QGIS v3.10 (QGIS Development Team, 2020) using the ocean, rivers + lake centerlines, lakes + reservoirs, and land vector polygons from naturalearthdata.com. Data for *Toxotes kimberleyensis* and *T. lorentzi* supplemented by GBIF data. Data for *T. mekongensis*, *T. siamensis*, and *T. sundaicus* added based on distributions described in Kottelat and Tan (2018). Round symbols indicate freshwater- and brackish-water-dwelling taxa. Square symbols indicate freshwater-dwelling taxa.

Few predation strategies are more striking than those of the archerfishes (Toxotidae). Archerfishes are well known for their ability to shoot a jet of water from their mouth, striking terrestrial prey such that they fall into the water for capture and consumption (Smith, 1936, 1945; Myers, 1952; Dill, 1977; Schuster et al., 2004; Schuster, 2007). Archerfishes will also shoot water at the benthos, dispersing sediment to dislodge buried prey (Dewenter et al., 2017). Found in the fresh- and brackish-water environments of the Indo-West Pacific (Fig. 1), these fishes attack a variety of organisms—from small arthropods to tetrapods, such as juvenile lizards (e.g., Gill, 1909; Smith, 1936; Salini et al., 1998). While the archerfish hunting behavior was initially noted by Schlosser (1764), a hypothesis of how these fishes shoot water was not proposed until H. M. Smith, with the assistance of G. S. Myers (see Smith, 1945; Myers, 1952), described two structures in the mouth of archerfishes—an elongate and narrow groove formed by soft tissue in the roof of the mouth, between the tooth patches on the endopterygoids (hereafter, palatal groove), and a basihyal with an elevation on its posterior aspect (Smith, 1936, 1945). Often called the “blowpipe” or “blowtube” hypothesis, a shot would be formed via a tube-like opening between the palatal groove and the hard and soft tissues of the basihyal. Although the blowpipe hypothesis proposed by Smith is the predominant hypothesis, the morphology of the shooting apparatus and the blowpipe hypothesis have been disputed. Elshoud and Koomen (1985:251) state that the ventral margin of the parasphenoid is nearly flat, allowing for “no good match” between the roof of the mouth and basihyal to gather and direct water. They also note that the palatal groove terminates at the posterior margin of the vomer, “nullifying” the effects of the groove for shooting. Instead, Elshoud and Koomen (1985) hypothesize that the archerfish shot is formed by an anterior aperture or break in the oral valves, calling this mechanism the “pressure tank” hypothesis. Although these two hypotheses appear to compete, particularly because Elshoud and Koomen (1985) explicitly refute Smith (1936, 1945) and Myers’ (1952) findings, the blowpipe and pressure-tank hypotheses may not be mutually exclusive and the palatal groove, basihyal, and oral valves may all contribute to the mechanism. In addition to questions about which oral structures make up the archerfish shooting mechanism, our current understanding of the behavior is based on the two of the most widespread archerfish species, the Banded Archerfish (*Toxotes jaculatrix*) and the Spotted Archerfish (*T. chatareus*). Only three of the ten currently recognized

 

species of archerfishes (*T. blythii, T. chatareus,* and *T. jaculatrix*) have been reported to perform this namesake hunting strategy (Kottelat and Tan, 2018). We do not know if all archerfishes possess the same oral structures, if anatomical variation is present among these oral structures, or how these features have evolved.

Previous investigations that sampled archerfishes focused on the interrelationships of the Toxotidae, which have allied the family among other perch-like fishes (Percoidei *sensu*Johnson, 1984; e.g., Greenwood et al., 1966; McAllister, 1968; Gosline, 1971; Springer and Orrell, 2004, Mirande, 2016; Girard et al., 2020). Early morphology-based hypotheses (e.g., Günther, 1860; Gosline, 1971) suggested several allies to the Toxot-idae, from the Chaetodontidae to the Nandidae, but explicit relationships were not necessarily identified. The works by McAllister (1968) and Mok and Shen (1983) allied the Toxotidae with all or a subset of the squamipinne fishes (*sensu*Mok and Shen, 1983), which included the families Acanthuridae, Chaetodontidae, Drepaneidae, Enoplosidae, Ephippidae, Girellidae, Kyphosidae, Monodactylidae, Pentacerotidae, Pomacanthidae, Scatophagidae, Scorpididae, Siganidae, and Zanclidae. Mok and Shen (1983) found osteological support for a sister-group relationship between the Kyphosidae and the Toxotidae based on the presence of an additional pharyngobranchial element on the second infrapharyngobranchial (their character 32), seven circumorbitals (their character 35), and an unbranched sensory canal in the lachrymal (their character 38). Subsequent DNA-based phylogenetic analyses recovered the archerfishes among a different clade of fishes that includes the Carangidae, Cynoglossidae, Latidae, Nematistiidae, and Psettodidae, among others (Chen et al., 2003, 2007; B. Li et al., 2009; Yagishita et al., 2009; C. Li et al., 2011; Betancur-R. and Ortí, 2014; Smith et al., 2016). Here, we follow the terminology used by Girard et al. (2020) and refer to this clade as the Carangiformes. While these DNA-based hypotheses consistently recover the Toxot-idae within the Carangiformes, archerfishes have been found sister to different carangiform clades, including the Latidae (Li et al., 2009; Rabosky et al., 2018), Istiophoridae and Xiphiidae (Near et al., 2013; Campbell et al., 2014), Menidae (Near et al., 2012), Nematistiidae (Li et al., 2011; Mirande, 2016; Smith et al., 2016), Psettodidae (Campbell et al., 2013), a clade containing Carangidae, Coryphaenidae, and Menidae (Chen et al., 2007), or a clade containing Carangidae, Paralichthyidae, and Pleuronectidae (Yagishita et al., 2009). However, as studies have sampled more carangiform families, including depauperate lineages, there has been growing evidence for the archerfishes being sister to the beachsalmons (Leptobramidae) based on various scales of genetic loci (Betancur-R. et al., 2013a, 2013b, 2017; Betancur-R. and Ortí, 2014; Harrington et al., 2016; Sanciangco et al., 2016) as well as a combination of morphology-based and DNA-based data (Girard et al., 2020). Girard et al. (2020) highlighted several anatomical characters supporting a sister-group relationship between the Leptobramidae and the Toxotidae (=Toxotoidei), including, but not limited to, the posterior placement of the basihyal and the absence of a medial extrascapular. While we are making progress in understanding the placement of the Toxotidae with respect to the larger carangiform radiation, there has yet to be a study focusing on the intrarelationships among archerfishes.

To date, three taxonomic studies have focused on describing and differentiating extant species of archerfishes (Allen, 1978, 2004; Kottelat and Tan, 2018). These studies emphasized external counts, pigmentation patterns, and measurements to separate species of *Toxotes*, with the most-recently described taxa, *T. mekongensis, T. siamensis,* and *T. sundaicus*, primarily differentiated from congeners based on flank pigmentation and body shape. With the emphasis on external characters, there is little information available regarding internal characters for the described species of archerfishes. Furthermore, no published works have included DNA-sequence data to test the current taxonomy of the Toxotidae or to hypothesize relationships among recognized species.

In this study, we ask the following questions: (1) Is the current taxonomy of the Toxotidae supported by DNA-sequence data?; (2) What are the intrarelationships among species of the Toxotidae?; (3) Is there variation in oral structures across archerfishes?; (4) Do the oral structures of archerfishes support either or both of the two functional hypotheses for how archerfishes shoot?; and (5) How do the intra- and inter-relationships of the Toxotidae inform the evolution of the archerfish's shooting structures? We first approach the taxonomy of archerfishes using new and previously published genetic data to test the limits of archerfish species. Considering these initial findings, we take an integrative approach, using a combination of hard- and soft-tissue discrete morphological characters and genetic data, to construct a phylogeny of archerfishes. Then, we highlight variation in the oral cavity of archerfishes and discuss previously described misinterpretations about archerfish oral structures. Finally, we use the resulting hypothesis of relationships and morphological findings to characterize the evolutionary history of this clade.

## Materials and methods

### Taxon sampling

We generated two partially overlapping molecular datasets and one morphological dataset in this study. The first molecular dataset will be called the “22-terminal” dataset and includes 11 taxa, including five outgroup taxa and six nominal species of the Toxotidae. Outgroup taxa were chosen based on the results of previous DNA-based (e.g., Betancur-R. et al., 2013a, 2013b; Harrington et al., 2016; Smith et al., 2016) and combined studies (i.e., Mirande, 2016; Girard et al., 2020) that recovered the Toxotidae among the carangiform fishes. These outgroups included taxa from the Centrarchidae, Latidae, Leptobramidae, Nematistiidae, and Percidae. Within archerfishes, four species were represented by multiple individuals including: *Toxotes blythii* (three individuals), *T. chatareus* (eight individuals), *T. jaculatrix* (two individuals), and *T. siamensis* (two individuals). In total, the 22-terminal dataset sampled six of the ten currently recognized species of archerfishes. Tissue samples for the remaining four nominal species of archerfishes were not available to be sampled. When constructing our second molecular dataset for the “13-terminal” analysis, we removed duplicate archerfish taxa, so each taxon was represented by one individual. Taxa in the morphological dataset were selected based on the results of the 22-terminal analyses and sampled 13 taxa. These include the five outgroup taxa from the 22-terminal analyses and eight archerfish species. We identified three specimens that match the paralectotype of *T. microlepis* (BMNH 1859.7.1.43) in lateral-line scale count, body shape, and pigmentation pattern (see Kottelat and Tan, 2018). However, Kottelat and Tan (2018) identified conflict between the lectotype and paralectotype of *T. microlepis*, causing us to question the identity of these specimens. Considering this, we did not include specimens of *T. microlepis* in this study. The molecular dataset for the 13-terminal dataset sampled ten taxa, including five outgroup taxa and five members of the Toxotidae. Taxa in the 13-terminal molecular dataset were selected based on the results of the 22-terminal analyses, locality information, specimens used in the morphological dataset, and amount of missing molecular data in the sample. Genetic data from *T. kimberleyensis*, *T. oligolepis*, and *T. sundaicus* were not available for analysis but were represented in the 13-terminal matrix by morphological data. Lists of taxa used in both the morphological and molecular components of this study can be found in the Material examined section and Supplementary Table 1, respectively. Symbolic codes for institutional resource collections follow Sabaj (2020). Both the morphological and molecular datasets were rooted with the centrarchid *Lepomis cyanellus.*

### Collection of morphological data

A novel morphological dataset was constructed for this study that included 100 hard and soft-tissue characters coded for all taxa sampled in the 13-terminal dataset. Of the 100 characters included in the morphological dataset, 36 characters were either explicitly coded from, based on, or modified from the following sources: McAllister, 1968; Patterson, 1970; Miller and Lea, 1972; Greenwood, 1976; Allen, 1978; 2004; Mok and Shen, 1983; Webb, 1989; Munroe, 1992; Westneat, 1993; Shinohara, 1994; Ross, 2001; Morgan and Gill, 2006; Springer and Smith-Vaniz, 2008; Pethiyagoda and Gill, 2013; Kimura et al., 2016; Märss et al., 2017; Kottelat and Tan, 2018; Rojo, 2018; Girard et al., 2020. All multistate characters were coded and analyzed as unordered characters. Descriptions of characters examined are listed in Supplementary File 1. Morphological characters were coded from formalin-fixed and ethanol-preserved specimens, disarticulated dry skeletons, dissected cleared-and-stained specimens, and formalin-fixed and ethanol-preserved specimens that were scanned using micro-computed tomography (µCT). We followed the methods described in Potthoff (1984) in preparing cleared-and-stained specimens with the modifications listed in Girard et al. (2020). Cleared-and-stained specimens were dissected following the protocol of Weitzman (1974) as it pertains to the circumorbital series, suspensorium, branchial basket, and pectoral girdle. Additionally, a subset of specimens were stained using the methods listed above but the “clearing” components of the protocol (i.e., steps involving trypsin) were omitted so soft-tissue features could be examined. The final morphological matrix (Supplementary Table 2) includes 1,306 of 1,313 possible entries and is 99% complete at the level of individual character states. Most of the missing character states are those that could not be determined from µCT samples.

As many archerfish species are rare in museum collections, it was not possible to sample every species via clearing and staining. To view and code anatomical features of these rare taxa, specimens were µCT scanned in batches of up to eight specimens at a time using either a Bruker SkyScan 1173 at the Karl F. Liem Bioimaging Center at the University of Washington's Friday Harbor Laboratories (FHL) and in association with the ScanAllFish project or a GE Phoenix v|tome|x m at the National Museum of Natural History (USNM). Scanning at FHL was performed using 65 kV, 123 µA, a 1.175 s exposure time, a 1 mm aluminum filter, and between 19.8 µm and 35.5 µm voxel size. The resulting image stacks were reconstructed into three-dimensional images using the software package NRecon. Three-dimensional reconstructions of the µCT scans were input into DataViewer v1.5.1.2 to isolate individual specimens from the batches they were scanned in without resizing or compression. Scanning at USNM was performed using 110 kV, 220 µA, a 200 ms exposure time, and a 74.8 µm voxel size. The resulting image stack was reconstructed into a three-dimensional image using the software package datos|x v2.4.0.1199. Specimen catalog numbers, preparation types, and MorphoSource media identifiers for the resulting µCT scans can be found in the Material examined section.

### Imaging of morphology

For specimens that were whole or stained, morphological features were examined with a Leica M205 C or a Nikon SMZ-745T microscope. Digital photography was used to visualize specimens and morphological features using a variety of imaging techniques. Images were taken using a Nikon D500 with either a Venus Optics Laowa 60 mm f/2.8 2X Ultra-Macro lens or a Venus Optics Laowa 25 mm f/2.8 2.5–5X Ultra-Macro lens. Specimens were illuminated by either daylight LED lighting (=5,000 K) from two eighteen-watt CREE Daylight bulbs or high-energy Royal Blue lighting (440–460 nm) emitted from two twelve-watt ABI Blue LED PAR38 bulbs plugged into two independent E26 lightbulb sockets. When anatomical features were difficult to view due to being poorly stained, surrounded by non-fluorescent soft tissue, or closely applied to other elements, these features were viewed and imaged under Royal Blue lighting to take advantage of the autofluroescent properties of alizarine staining, similar to those shown in Girard and Smith (2016) and Ponssa and Abdala (2020). To view and image features under Royal Blue lighting, we followed the protocol established by Smith et al. (2018) and the filter modifications listed by Girard et al. (2020). To overcome the small depth of field in high magnification images, several images were photographed via z-stacking (also known as focus stacking; see Smith et al., 2018). Images for z-stacking were taken using the camera, lens, and optional filter combinations listed above with the camera attached to a WeMacro 100 mm focus stacking rail controlled by a Cognisys Stackshot Controller and Helicon Remote v3.9.5. Digital images at different focal distances were then algorithmically combined into a single composite image using Helicon Focus v6.7.1.

For µCT scanned specimens, the resulting isolated specimen image stacks were viewed and manipulated for lighting and opacity using CTvox v3.0 (Bruker) or the SlicerMorph module (Rolfe et al., 2021) in 3D Slicer v4.13.0 (Fedorov et al., 2012). Hard tissues were coded and imaged using the tools in CTvox, with custom transfer functions, or in 3D Slicer by modifying the region of interest (ROI) box in the “volume rendering” module. For specific anatomical elements, features were segmented from the surrounding scanned specimen using 3D Slicer. All morphology that was segmented with 3D Slicer were first isolated from the larger scan using the ROI feature in the “volume rendering” module. These ROIs were separated from the background using the “threshold” or “grow from seeds” functions within the “Segment Editor” module. For the “threshold” function, the osteological feature was further isolated using the scissors tool and then visualized using the “volume rendering” module in 3D Slicer.

### DNA extraction, locus amplification, and sequence alignment

A DNeasy Tissue Extraction Kit (Qiagen) or a Maxwell RSC Blood DNA Kit and Instrument (Promega) were used to extract DNA from the tissue samples of archerfishes following manufacturers’ extraction protocols. The first or combined first and second elution from a Qiagen filter were dried to a volume of 102 µL using a Savant DNA120 SpeedVac Concentrator (Thermo Scientific). The combined elution increased the amount of DNA collected. Once the DNA was extracted, 2 µL of the DNA extracts were quantified using a Qubit Fluorometer 2.0 (Invitrogen) with the Qubit dsDNA BR Assay Kit (Invitrogen). Final quantified samples (100 µL in volume) were sent to Arbor Biosciences for library preparation (e.g., DNA shearing, size selection, cleanup), target capture, enrichment, sequencing on an Illumina HiSeq 2500 or NovaSeq 6000 (Illumina), and demultiplexing. The 500 ultraconserved element (UCE) actinopterygian-loci probe set (Faircloth et al., 2013) was used for target capture.

Sequence data were provided as compressed FASTQ formatted files of demultiplexed sequence data from multiple runs from Arbor Biosciences. These data were uncompressed and combined into two read files for each newly sequenced taxon. Two read files were cleaned of adapter contamination and low-quality bases using the parallel wrapper illumiprocessor v2.10 (Faircloth, 2013) around trimmomatic v0.39 (Bolger et al., 2014). Cleaned sequencing reads were submitted to GenBank and have been assigned SRA Accession Numbers SRR17680273–SRR17680287 under BioProject PRJNA798425 (Supplementary Table 1). Cleaned reads and previously published UCE data, obtained from Girard et al. (2020; BioProject Accession Number PRJNA604383; Supplementary Table 1), were assembled using a Python script (assemblo_spades.py) in PHYLUCE v1.7.0 (Faircloth et al., 2012; Faircloth, 2016) and the short paired-end read sequence assembler SPAdes v3.14.1 (Prjibelski et al., 2020) with the default settings. Once assembled, a relational database that contained all probes was built using a Python script (match_contigs_to_probes.py, PHYLUCE) and LASTZ v.1.0.4 (Harris, 2007) to identify taxon-specific contigs within the assembled UCE loci. Minimum coverage and minimum identity for identifying UCEs were set to 80%. The relational database was searched using the PHYLUCE script get_match_counts.py to generate a list of UCE loci shared among all taxa. This list was input into the PHYLUCE script get_fastas_from_match_counts.py to create a single FASTA file containing all UCE sequence data for all taxa. The data in this file were divided by locus using explode_get_fastas_file.py within PHYLUCE and aligned using MAFFT v7.475 (Katoh and Standley, 2013). Each locus alignment that contained data from a minimum of 14 taxa for the 22-terminal and six taxa for the 13-terminal dataset (minimally 65% complete) was converted into PHYLIP-format files for analyses.

In addition to UCE loci, we extracted mitochondrial gene fragments of cytochrome oxidase subunit 1 (hereafter, COI) from high-throughput cleaned sequencing reads received from Arbor Biosciences. Recent studies have performed similar extractions of these data to verify identification and include additional character information in the analyses (e.g., Ghedotti et al., 2018; Martin et al., 2018; Girard et al., 2022). Gene fragments were extracted from cleaned reads using Geneious v11.1.5 (Kearse et al., 2012) and the protocol outlined in Girard et al. (2022). Homologous regions collected were cleaned of ambiguities and inspected for stop codons using Geneious. These extracted COI sequences are available in Supplementary File 2. Sequences that correspond to taxa within the 22-terminal and 13-terminal datasets were then collated into a PHYLIP file aligned with MAFFT v7 (Katoh and Standley, 2013).

### Partitioning schemes and nucleotide substitution models

For the 22-terminal dataset, 433 aligned UCE loci were analyzed. For the 13-terminal dataset, 458 aligned UCE loci were analyzed. Mean sequence fragment length was 1,300 base pairs (bps), with a range of 146–3,853 bps (Supplementary Table 1) across all UCE loci. For the 22-terminal dataset, UCE loci were concatenated into a single matrix that contained 596,707 bps in length (≈73% complete) and 47,396 parsimony-informative sites. For the 13-terminal dataset, UCE loci were concatenated into a single matrix that contained 655,372 bps in length (≈78% complete) and 41,802 parsimony-informative sites. The two matrices of UCE loci were partitioned using the sliding-window site characteristics–entropy method (Tagliacollo and Lanfear, 2018), which split each UCE locus into left and right flanking regions and the ultraconserved core (i.e., center segment). The left, central, and right UCE segments were used as input data blocks for PartitionFinder v2.1.1 (Lanfear et al., 2014, 2017; Stamatakis, 2014) to find the best-fitting nucleotide substitution model for each data partition. The following PartitionFinder parameters were set to the subsequent option: branchlengths set to linked; models set to GTR, GTR + G, and GTR + I + G; model_selection set to AICc; schemes search set to rclusterf. PartitionFinder was used with the default setting along with –raxml. The output from PartitionFinder listed 409 subsets for the 22-terminal matrix and 400 subsets for 13-terminal matrix, both with associated models for these regions. A list of the subsets of UCEs, partitions, and associated models can be found in Supplementary Tables 3 and 4.

The two mitochondrial gene matrices both resulted in a 655 bp alignment. The 22-terminal dataset was ≈96% complete and contained 203 parsimony-informative sites. The 13-terminal dataset was ≈ 94% complete and contained 191 parsimony-informative sites. Both COI matrices were broken into three partitions: one partition designated for each of the three codon positions in the protein-coding locus. These three partitions were then used as input for PartitionFinder v2.1.1 (Lanfear et al., 2014, 2017; Stamatakis, 2014) using the same settings as above. PartitionFinder designated three subsets with associated models for both matrices. These partitions and associated models are listed in Supplementary Tables 3 and 4.

### Analysis of molecular data matrices

**Fig. 2:**
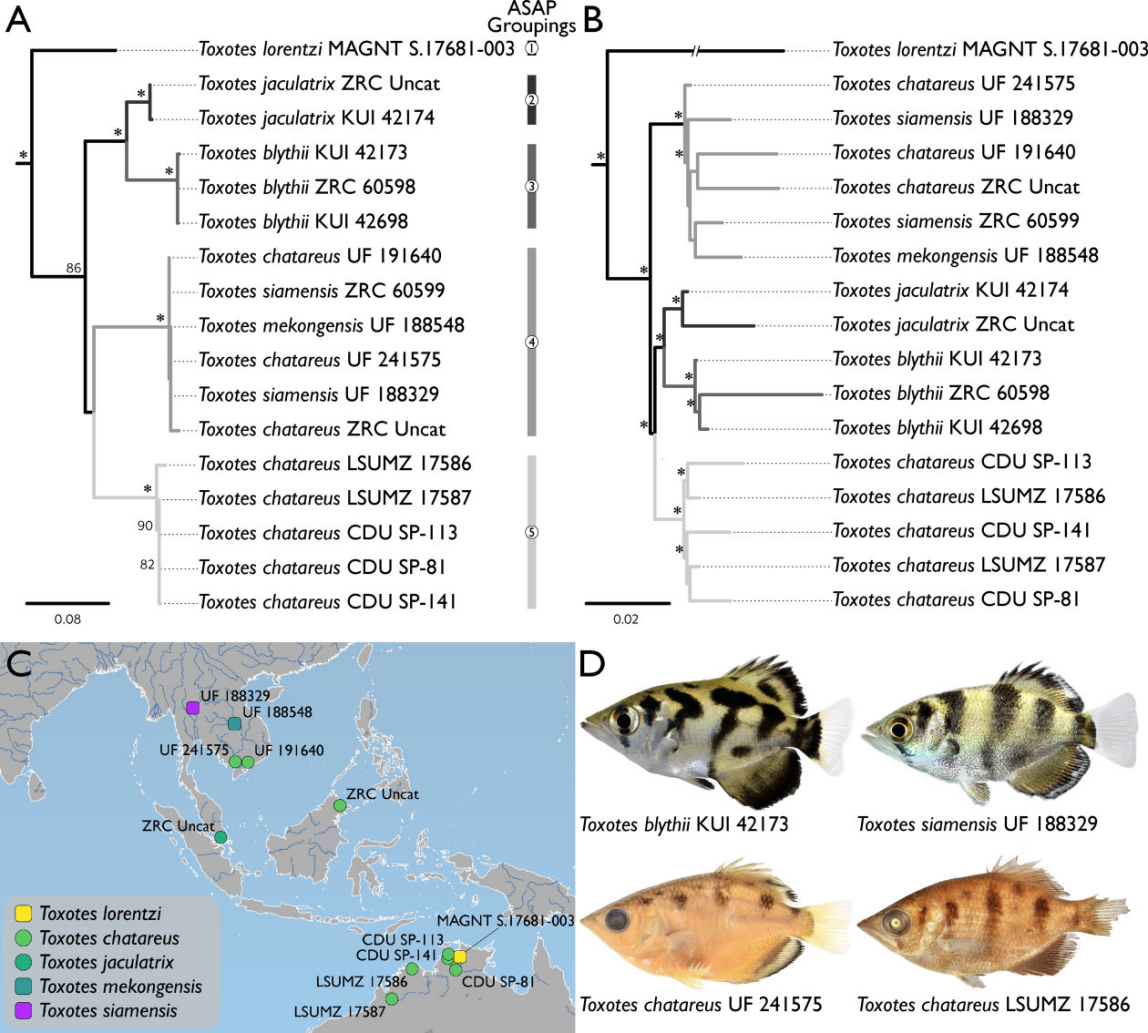
Hypotheses of relationships from partitioned likelihood analysis of archerfishes (Toxotidae) in the 22-terminal dataset. All specimens from Charles Darwin University marked as “CDU.” (A) Dataset included one mitochondrial locus. Support was determined based on 200 bootstrap replicates. All nodes in the support analysis that yielded bootstrap values ≥95% are indicated by a “*.” Phylogeny truncated to only show the Toxotidae. Group designations from ASAP shown by bars on right-side of phylogeny. Branches of phylogeny colored to match group designations from ASAP. (B) Dataset included 433 UCE loci. Support was determined based on 200 bootstrap replicates. Branch with “//” is reduced in length by half. All nodes in the support analysis that yielded a bootstrap value ≥95% are indicated by a “*.” Phylogeny truncated to only show the Toxotidae. Branches of phylogeny colored to match group designations from ASAP. (C) Map shows areas where samples included in the above analyses were collected. Samples from the aquarium trade are excluded from the map. Map generated in QGIS v3.10 (QGIS Development Team, 2020) using the ocean, rivers + lake centerlines, lakes + reservoirs, and land vector polygons from naturalearthdata.com. Round symbols indicate freshwater- and brackish-water-dwelling taxa. Square symbols indicate freshwater-dwelling taxa. (D) Photos of vouchers included in analyses under white or daylight LED light. Images not to scale. Photo of UF 188329 voucher courtesy of Zachary S. Randall, Florida Museum of Natural History, with modification.

Following assembly and partitioning of DNA-based matrices, we performed two different sets of analyses of the 22-terminal dataset. We first analyzed the 22-terminal COI data matrix and an independent partition model file using IQ-Tree v2.1.2 (Chernomor et al., 2016; Minh et al., 2020). The model file included the three PartitionFinder-designated subsets and associated models. We performed 20 independent runs of IQ-Tree, setting the modification number of unsuccessful iterations to stop (-nstop) to 2,000. We then used the resulting trees from these analyses as starting trees for a second set of analyses in IQ-Tree. These starting trees, along with the matrix and partition model file, were analyzed with the following settings: nearest-neighbor interchange search (-allnni), number of trees in the candidate set to maintain during tree search (-nbest) to 25, and number of unsuccessful iterations to stop (-nstop) set to 5,000. Support for the resulting topology with the best likelihood score was assessed by analyzing 200 standard bootstrap replicates (-bo). The second set of analyses of the 22-terminal dataset analyzed the UCE sequences using IQ-Tree and an independent partition model file that included the 409 subsets and associated models. Analyses were conducted using the same methods as described for the COI matrix above. The phylogeny with the best likelihood score from both analyses was visualized with FigTree v1.4.4 (Rambaut, 2012). All standard bootstrap replicates were reconciled with the best-fitting phylogeny and the resulting bootstrap replicates using IQ-Tree (-con; Fig. 2).

### Species-hypotheses analysis

To objectively propose species boundaries of taxa within the 22-terminal dataset, we used the software Assemble Species by Automatic Partitioning (ASAP; Puillandre et al., 2021) to independently analyze the mitochondrial locus. We used the software via the web interface (https://bioinfo.mnhn.fr/abi/public/asap/) to analyze the data three times, once using each of the JC69, K80, and p-distance options.

### Combined analysis of morphological and molecular data matrices

Considering the results from the 22-terminal analyses, we refined the identity and taxonomy of archerfishes and simultaneously analyzed the 13-terminal COI, UCE, and morphological matrices using IQ-Tree and an independent partition model file. The number of partitions in the combined analysis was 404 (400 from the UCE matrix). Analyses in IQ-Tree were conducted using the same methods as described for the 22-terminal datasets with one exception: taxa represented by exclusively morphological data (see above) were excluded from the support analysis.

### Character optimization

**Fig. 3:**
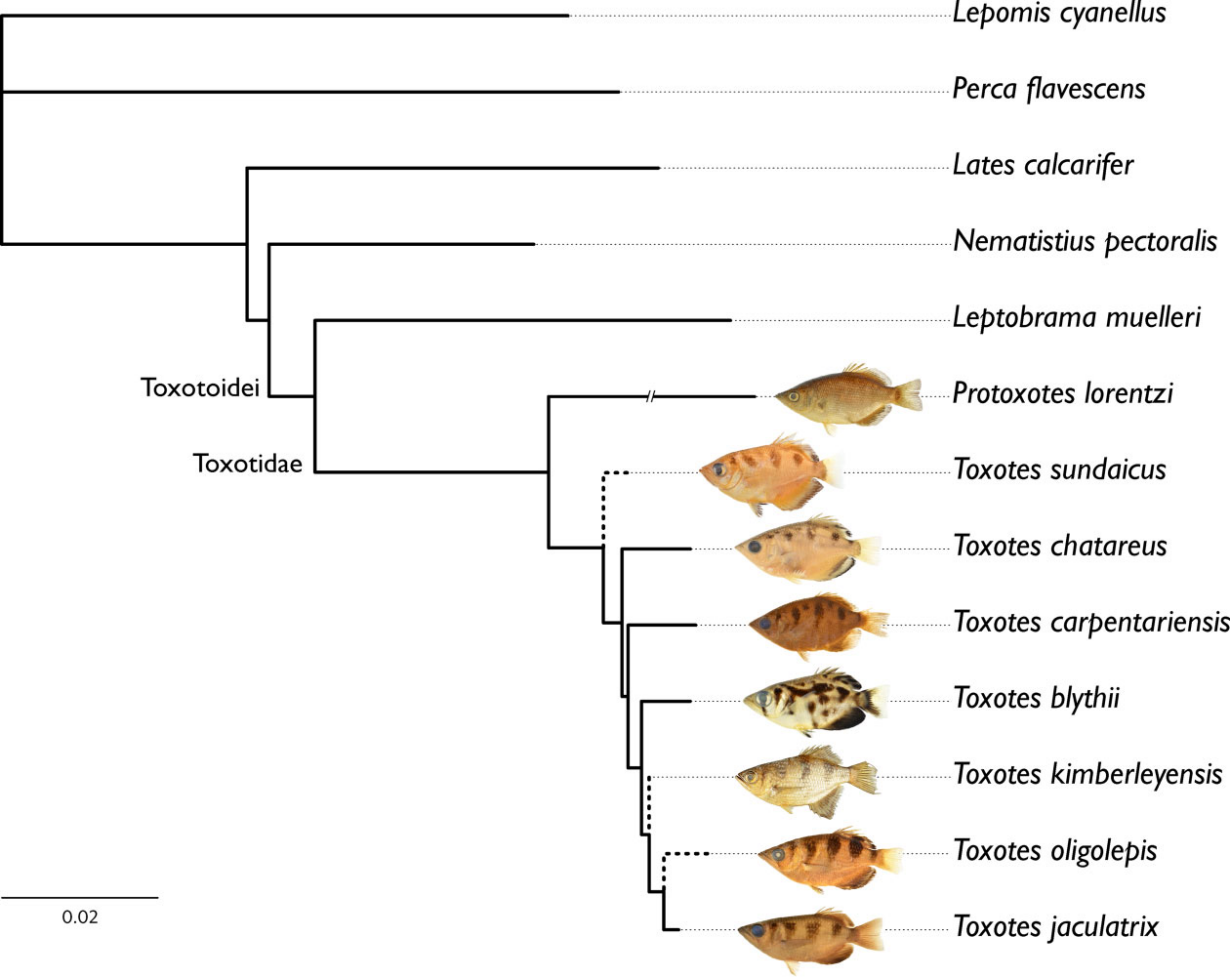
Hypothesis of relationships from partitioned likelihood analysis of archerfishes (Toxotidae) and outgroup taxa from the 13-terminal dataset. Dataset included 100 morphological characters, 458 ultraconserved element loci, and one mitochondrial locus. Support was determined based on 200 bootstrap replicates. All nodes included in the support analysis yielded a bootstrap value ≥99%. Branch with “//” is reduced in length by half. Dashed branches indicate terminals that were represented only by morphological data that were excluded from the bootstrap analyses. Catalog numbers for the images of specimens as they appear in the figure: USNM 406792; ZRC 42270 paratype; UF 241575; USNM 173503; LSUMZ 17019; AMS I.42570–001 paratype (courtesy of Kerryn Parkinson and Amanda Hay, Australian Museum, with modification); SU 29567; CAS 206640. Specimen images not to scale.

**Fig. 4:**
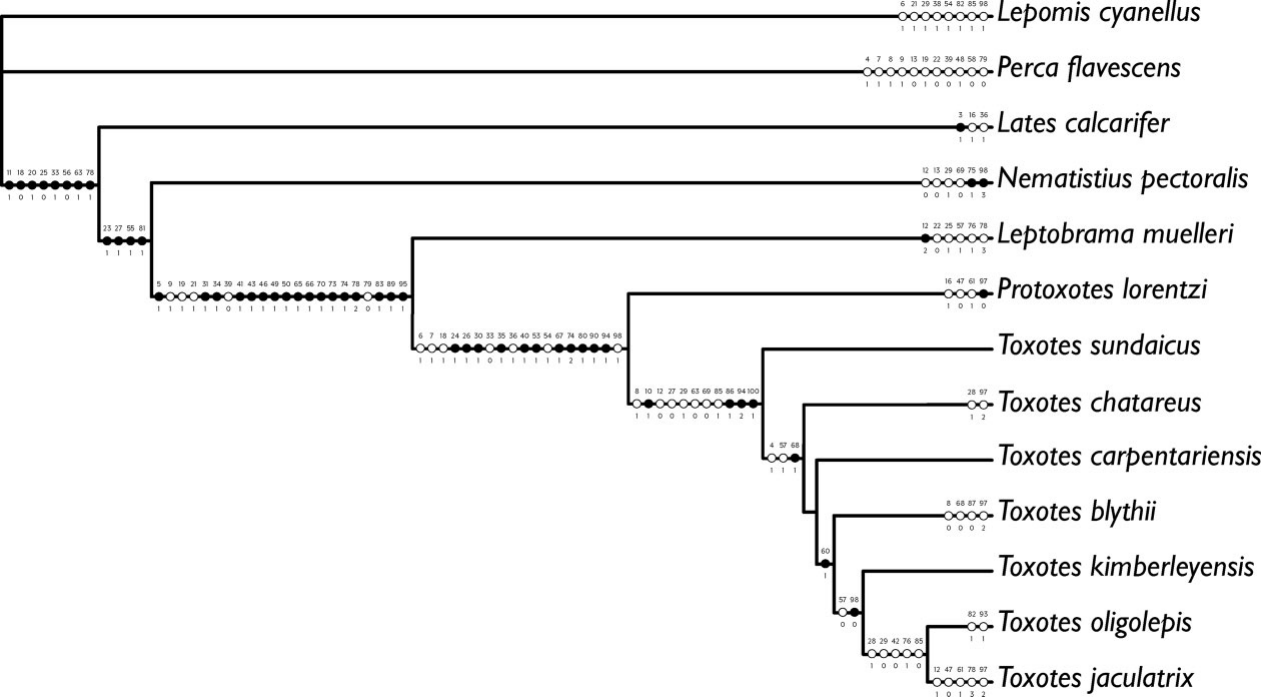
Hypothesis of relationships from partitioned likelihood analysis of archerfishes and outgroup taxa from the 13-terminal dataset. Morphological characters optimizing unambiguously onto each branch are represented by a circle with the corresponding character number listed above and corresponding character state below. Circles with black fill are unique and unreversed character states. Circles with white fill are states that optimize multiple times in the phylogeny.

With the inferred phylogeny of archerfishes, we used the resulting tree topology and our morphological matrix (Figs. 3 and 4, Supplementary Table 2) as input data for WinClada v1.00.08 (Nixon, 2002) to view morphological transformations across the phylogeny. The characters were mapped onto our topology using a parsimony optimization and the WinClada option that allows for unambiguous changes only. We also used WinClada to view fast and slow optimizations (i.e., ACCTRAN and DELTRAN, respectively) of characters. However, we will focus on the unambiguous changes in the following sections with this optimization shown in Fig. 4.

## Results

Hereafter, order- and suborder-level terminology follow Girard et al. (2020).

### Species of archerfishes and taxonomic changes

The hypotheses of relationships for the 22-terminal dataset are shown in Fig. 2. The support analyses of the COI matrix yielded 11 nodes (≈57%) with a bootstrap value of ≥80% and seven nodes (≈36%) with a bootstrap value of ≥95%. The support analyses of the UCE matrix yielded 15 nodes (≈78%) with a bootstrap value of ≥95%. The resulting topologies from both analyses support five archerfish lineages: a clade that includes *Toxotes lorentzi*; a clade that includes all samples of *T. blythii*; a clade that includes all samples of *T. jaculatrix*; a clade that includes all samples of *T. mekongensis, T. siamensis,* and the non-Australian *T. chatareus*; a clade that includes all samples of the Australian *T. chatareus* (Fig. 2). The results from the three executions of the ASAP software designated a set of species grouping options with associated p-values (Fig. 2A). All three executions of the ASAP software yielded the lowest p-value also in support of five groups of archerfishes within our analysis (JC69 = 0.0101, K80 = 0.0152, dist = 0.0166).

Based on the results from the 22-terminal analyses, we treat *T. mekongensis*Kottelat and Tan 2018 and *T. siamensis*Kottelat and Tan 2018 as synonyms of *T. chatareus* (Hamilton 1822)*.* Additionally, we recognize *T. carpentariensis* Castelnau 1878 as a species of archerfish that represents a southern or Australian lineage of the specimens typically referred to as *T. chatareus* following Allen (1978). Finally, due both morphological and genetic difference between *T. lorentzi* and the remaining species of archerfishes and its placement as the sister species to all other archerfishes, we recognize the genus *Protoxotes*Whitley 1950 for this taxon to highlight its distinctiveness and the similarity among species of *Toxotes*. The following sections discuss these taxonomic changes. Subsequent analyses, results, and discussion in this study follow this revised taxonomy for the Toxotidae.

### Phylogeny of archerfishes

The hypothesis of relationships for the 13-terminal analysis is shown in Figs. 3 and 4. All nodes that were recovered in the support analysis yielded a bootstrap value of ≥99%. The resulting topology from the 13-terminal analysis showed a monophyletic Toxotidae in the carangiform suborder Toxotoidei that is sister to the Leptobramidae (Figs. 3 and 4). *Protoxotes lorentzi* is recovered as the sister group to a monophyletic *Toxotes. Toxotes sundaicus* is recovered as the sister group of all other *Toxotes*. Within *Toxotes,* we recover *T. chatareus*, *T. carpentariensis, T. blythii,* and *T. kimberleyensis* in a grade leading to a clade composed of *T. jaculatrix* sister to *T. oligolepis*.

To examine character evolution within archerfishes and allies, morphological characters were optimized onto the topology (Fig. 4). Of these character transformations, 52 of 125 (≈41%) are unique and unreversed (Fig. 4). The following section discusses a subset of characters that optimize in support of the relationships recovered with respect to the Toxotoidei.

### Morphological variation in archerfish oral structures

**Fig. 5:**
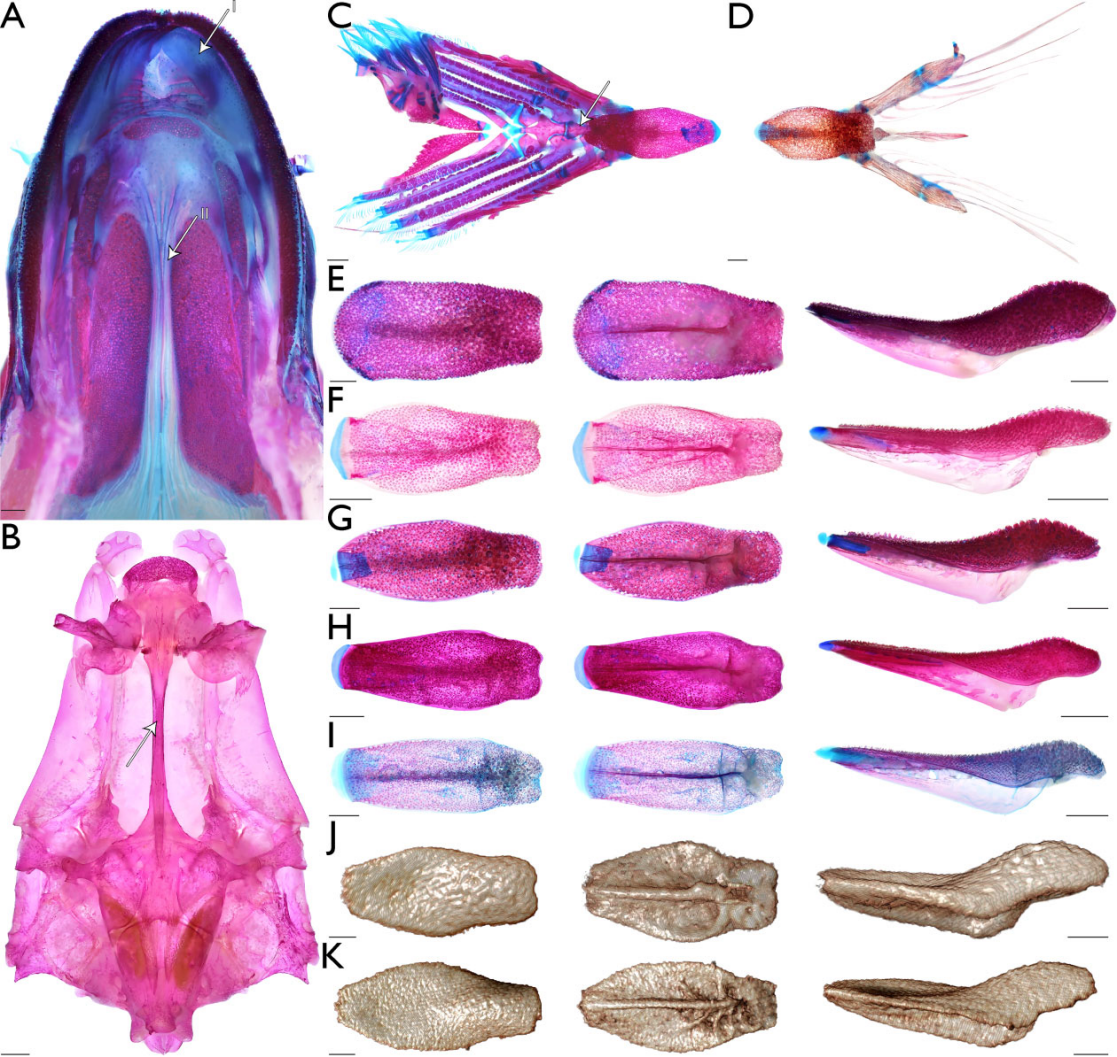
Morphological variation in archerfish oral structures. Images of stained specimens under white or daylight LED light. Images of µCT-scanned specimens generated using CTvox. Oral valves and palatal groove in dorsal aspect of oral cavity—(A) *Toxotes chatareus* (KUI 42697), arrow I points to left dorsal oral valve, arrow II points to palatal groove, ventral view, scale bar 1 mm. Groove in ventral aspect of parasphenoid (character state 5_1_)—(B) *T. jaculatrix* (FMNH 69510), arrow points to groove in parasphenoid, ventral view, scale bar 1 mm. Basihyal inserts above basibranchial one and covers the first basibranchial when viewing the branchial and hyoid arches dorsally (character state 50_1_)—(C) *T. chatareus* (KUI 42697), arrow points to basibranchial three, dorsal view, scale bar 1 mm; (D) *T. carpentariensis* (USNM 454834), dorsal view, scale bar 1 mm. Shape and anatomical variation in the archerfish basihyal (characters 40–45 and 49). For all images of an isolated basihyal, leftmost column shows the dorsal view of basihyal with anterior margin to the left; central column shows the ventral view of basihyal with anterior margin to the left; rightmost column shows the left lateral view of basihyal with anterior margin to the left; scale bar 1 mm—(E) *Protoxotes lorentzi* (USNM 454833); (F) *T. blythii* (KUI 42173); (G) *T. chatareus* (UMMZ 236673); (H) *T. jaculatrix* (KUI 42174); (I) *T. oligolepis* (SU 29567); (J) *T. kimberleyensis* (WAM P.2620–001); (K) *T. sundaicus* (ZRC 42270).

We found that all species of archerfishes possess an elongate and narrow palatal groove and an enlarged and toothed basihyal with an elevation on the posterior aspect (Fig. 5). The soft-tissue palatal groove extends from near the posterior aspect of the endopterygoids to near the anterior margin of the palatine tooth plates (Fig. 5A) and is associated with an indentation or groove in the ventral aspect of the parasphenoid (Fig. 5B). The soft tissue associated with the palatal groove is robust in cross section and there is an abundance of tissue within the groove such that it folds onto itself (Fig. 5A). The groove tissue is considerably flexible, even in fixed specimens. The lateral walls of the bony parasphenoid groove originate near the medial part of the orbit, becoming most pronounced anteriorly and before the end of the parasphenoid (Fig. 5B). The ventral margin of the vomer also possesses a groove posterior to the vomerine tooth plate, but this groove is reduced compared to the parasphenoid groove. The soft tissue overlying this groove is largely smooth and slopes towards the vomerine tooth plate (Fig. 5A). Finally, the posterior aspect of the vomerine tooth plate is indented mesially, near the termination of the vomerine groove (Fig. 5A and 5B). A large basihyal occupies most of the oral cavity in all archerfishes. The overall topology of the dorsal aspect of the basihyal, the posterior elevation of the basihyal, the presence of teeth on the basihyal, and the presence of soft tissue surrounding the basihyal are conserved across all toxotid species examined (Fig. 5C–5K). When moving across the basihyal from the posterior to anterior aspect, the posterior elevation of the basihyal descends to the middle of the element and a concave or trough-like region. This concave region of the basihyal then ascends to the anterior aspect of the element, which is flattened and plate-like. The lateral aspects of the basihyal ascend from the concave region before quickly descending immediately before the lateral margins of the basihyal. All toxotids have a prominent keel on the ventral margin of the basihyal, extending from the anterior margin to near the posterior margin of the element. This keel's maximum depth is near the latter ¼ of the basihyal length before quickly ascending near the posterior aspect of the basihyal and near where the element contacts the first basibranchial. These keels may be laterally flanked by fossae or ridges of bone that serve as attachment points for soft tissue that surrounds the basihyal. While many aspects of the basihyal are conserved, variation in the amount of tissue surrounding the basihyal, position of the basihyal rostral cartilaginous cap, and the location in which the basihyal obtains its maximum width is present across species of archerfishes. The soft tissue surrounding the basihyal is robust and forms a smooth and gasket-like rim in *Protoxotes lorentzi* and species of *Toxotes*. However, *P. lorentzi* possess a narrow rim of soft tissue that is rounded and ventrally directed compared to the wide and dorsolaterally directed margin of tissue in *Toxotes*. *Protoxotes lorentzi* (Fig. 5E) possesses a robust basihyal that is somewhat tapering posteriorly in overall shape. The rostral cap of the element is well ossified, with only a slight rim of cartilage (i.e., alcian stained) that was ventrally displaced. The basihyal is broad overall anteriorly, with dentition present up to the margin, and the posterior margin is slightly concave mesially. *Toxotes sundaicus* possesses an ovoid basihyal that is not as broad anteriorly as *P. lorentzi*. The basihyal in *T. sundaicus* achieves its greatest width within the anterior ⅓ to ½ of the length of the element. While we could view the posterior margin of the element is straight to convex, we could not identify if the rostral cap of the basihyal was cartilaginous in the µCT scan of *T. sundaicus*. However, when examining two formalin-fixed and ethanol-preserved specimens under daylight LED light, the consistency, position, and appearance of the rostral aspect of the basihyal is consistent with other species of *Toxotes*. Therefore, we consider *T. sundaicus* (Fig. 5K) to possess a cartilaginous basihyal cap in line with the dorsal margin of the basihyal. A similarly shaped basihyal to *T. sundaicus* is found in *T. blythii*, *T. carpentariensis, T. chatareus,* and *T. kimberleyensis* (Fig. 5D, 5F–5G, 5J, and 5K), which have an ovoid element that achieves its greatest width within the anterior ⅓ to ½ of the length of the element. While the basihyal in these species have a cartilaginous rostral cap that is in line with the dorsal margin of the element, basihyal dentition is absent from the anterior margin of the ossified element in *T. blythii* and *T. carpentariensis*. For *T. kimberleyensis*, we could not observe the extent of the basihyal dentition in the µCT scan or under polarized light and further investigation is needed for this character. The basihyal in *T. jaculatrix* and *T. oligolepis* (Fig. 5H and 5I) obtains its greatest width posteriorly to medioposteriorly when compared to other species of *Toxotes,* with the greatest width of the element obtained within the posterior ⅓ of the element's length. In *T. jaculatrix* and *T. oligolepis,* the dentition on the basihyal reaches the anterior margin of the ossified element. A cartilaginous rostral cap was observed in the cleared-and-stained specimens of *T. jaculatrix* and *T. oligolepis*, and it was in line with the dorsal margin of the basihyal*.* Additionally, the posterior margin of the basihyal in *T. jaculatrix* and *T. oligolepis* is concave and like other archerfish species.

## Discussion

### Species of archerfishes and nomenclature

**Fig. 6:**
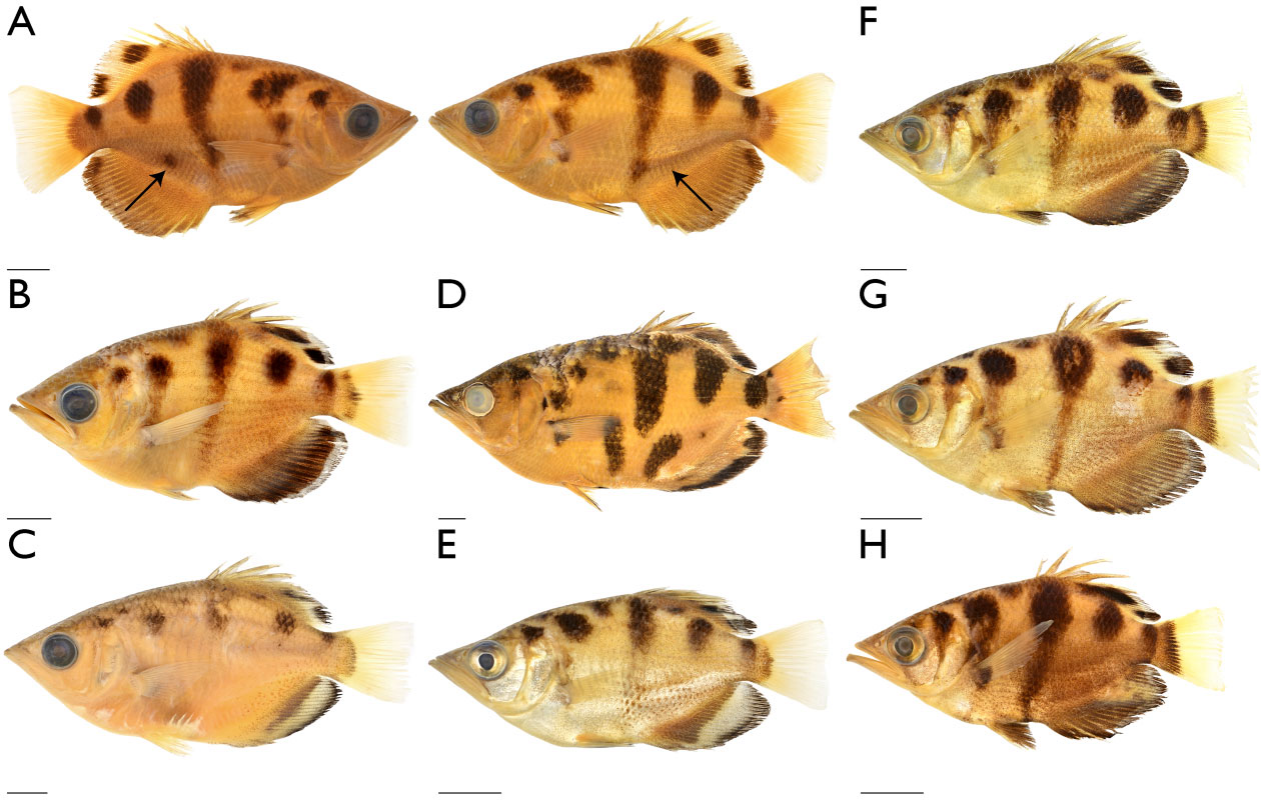
Variation in flank pigmentation among *Toxotes chatareus* collected in Asia and Oceania. Images of whole ethanol specimens under white or daylight LED light. Scale bars 1 cm. (A) UMMZ 236673, arrow points to presence and absence of pigmentation spot above anal fin on each flank, from Thailand, right lateral view and left lateral view; (B) CAS 97101, from Laos, left lateral view; (C) UF 241575, from Vietnam, left lateral view; (D) CAS 93958, from Thailand, left lateral view; (E) UMMZ 241226, from Vietnam, left lateral view; (F) UMMZ 232688, from Cambodia, left lateral view; (G) UMMZ 232688, from Cambodia, left lateral view; (H) UMMZ 232688, from Cambodia, left lateral view.

The genus *Toxotes* was described by Cloquet (1816) and Cuvier (1816; see Kottelat, 2013), with the type species of *Sciaena jaculatrix* Pallas 1767 by monotypy, and most synopses have included all species of archerfishes in a single monogeneric family. While the taxonomy of the Toxotidae has been largely stable, a second genus of archerfish and several new species have been named, including three taxa recently described by Kottelat and Tan (2018). The first analyses in our study, which exclusively used DNA-based characters in the 22-terminal dataset, tested the species limits within the Toxotidae, recovering a monophyletic family with five groups of archerfish (Fig. 2). Our sampling of *Toxotes* from Asia and Oceania included *T. blythii* (three individuals), *T. chatareus* (three individuals), *T. jaculatrix* (two individuals), *T. mekongensis* (one individual), and *T. siamensis* (two individuals). These individuals were collected from disparate localities, including Cambodia, Malaysia, Thailand, and Vietnam (see Fig. 2). While samples of *T. blythii* and *T. jaculatrix* formed reciprocally monophyletic groups, the remaining samples from Asia and Oceania were found in a single clade that included three described species. *T. mekongensis* and *T. siamensis* were recently described as distinct species based on pigmentation and squamation, among other measurements (Kottelat and Tan, 2018), but genetic data was not included with their description. Our results from the 22-terminal analyses find *T.mekongensis* and *T. siamensis* in the same lineage as *T. chatareus* from Asia. Samples from these taxa differed by at most eight bps at the COI locus, compared to the >60 bp differences between these samples and other species of archerfishes. In addition to the genetic similarity among *T. chatareus*, *T. mekongensis*, and *T. siamensis*, we found inconsistencies in the pigmentation patterns for *T.mekongensis* and *T. siamensis* and few internal morphological characters that differentiated these taxa from *T.chatareus*. One of the diagnostic features of *T. siamensis* is the shape and elongation of the second vertical pigmentation area along the flank, along with a large black spot above the anterior-half of the anal-fin base (Kottelat and Tan, 2018). We found several specimens of *T. siamensis* that inconsistently possessed the black spot above the anal-fin base between the left and right flanks (Fig. 6A) in addition to variation in the shape of the second pigmentation area on the flank. This pigmentation inconsistency was further confounded by the overall variability in pigmentation among specimens of *T. chatareus*, *T. mekongensis*, and *T. siamensis* from Asia and Oceania, which can be seen in Fig. 6, including those caught in the same location (Fig. 6F–6H). Finally, an individual from Lake Argyle of the Australian Ord River system, which resembles the pigmentation pattern of *T. siamensis* from Asia and Oceania, is shown by Merrick and Schmida (1984, fig. 225). These authors briefly discuss pigmentation-pattern variation within *Toxotes*, highlighting that pigmentation may change in a single individual due to stress and/or light availability (Merrick and Schmida 1984, figs. 223 and 224), a trait authors of this study have observed in *T.carpentariensis* and *T. chatareus*. Although pigmentation is currently an important character for differentiating species of archerfishes, we caution the use of these traits as diagnostic at the species level going forward. Based on the genetic and morphological data collected in this study, we treat *T.mekongensis* and *T. siamensis* as junior subjective synonyms of *T. chatareus*.

Our sampling of Australian archerfishes included six individuals from both Western Australia and the Northern Territory (see Fig. 2). Of these six samples, five were identified as *T.chatareus* based on lateral-line counts and pigmentation (see Allen, 1978, 2004). These five samples were recovered in a single clade separate from all other species of *Toxotes*. We were surprised by this finding because we were unable to find consistent external morphological features, including lateral-line scale counts or pigmentation, that separated these samples from *T.chatareus* collected in Asia and Oceania. While Allen (1978) notes that speciation may be occurring among the populations of *T. chatareus* in Australia and New Guinea based on fin-spine morphology, we did not find consistent differences in spine morphology between specimens collected from Asia and Oceania and those from Australia and New Guinea. While some differences occur in the length of the pelvic axillary “scale” between these two lineages, additional research is needed to find diagnostic morphological characters to differentiate these two lineages of *Toxotes*. A current synonym of *T.chatareus* is *T.carpentariensis* Castelnau 1878*,* which was described from a single adult specimen in the Norman River, Queensland, Australia. The description of this taxon is brief, and the holotype was examined by Allen (1978), who concluded that the species was synonymous with *T. chatareus* from the Kimberley region of Western Australia and the Gulf of Carpentaria drainage system. Based on geography and genetic differences to *T.chatareus* from Asia and Oceania, we treat these southern samples as *T.carpentariensis*.

Previous researchers debated whether *T. lorentzi* is a member of the genus *Toxotes* or in a different genus, *Protoxotes*. Whitley (1950:244) placed the taxon in the genus *Protoxotes* to represent the “most primitive” species of archerfish based on the following combination of characters: absence of lateral pigmentation along the flank, straight lateral line along the flank, small size of scales, anterior position of the dorsal fin, and a more elongate body. However, subsequent authors (Taylor, 1964; Allen, 1978) placed the taxon within the genus *Toxotes*, which is the current generic designation. The results from our analyses recover *T. lorentzi* as the sister taxon to all other species of archerfishes based on both genetic and morphological traits. Therefore, we resurrect the genus *Protoxotes*Whitley 1950 for this taxon. In the following paragraphs, we discuss the inter- and intra-relationships of the archerfishes organized by the topology we recover in our 13-terminal analysis. In each of these sections, we highlight one or more morphological features that optimize in support of the relationships we recover.

### Monophyly of the Toxotoidei

**Fig. 7:**
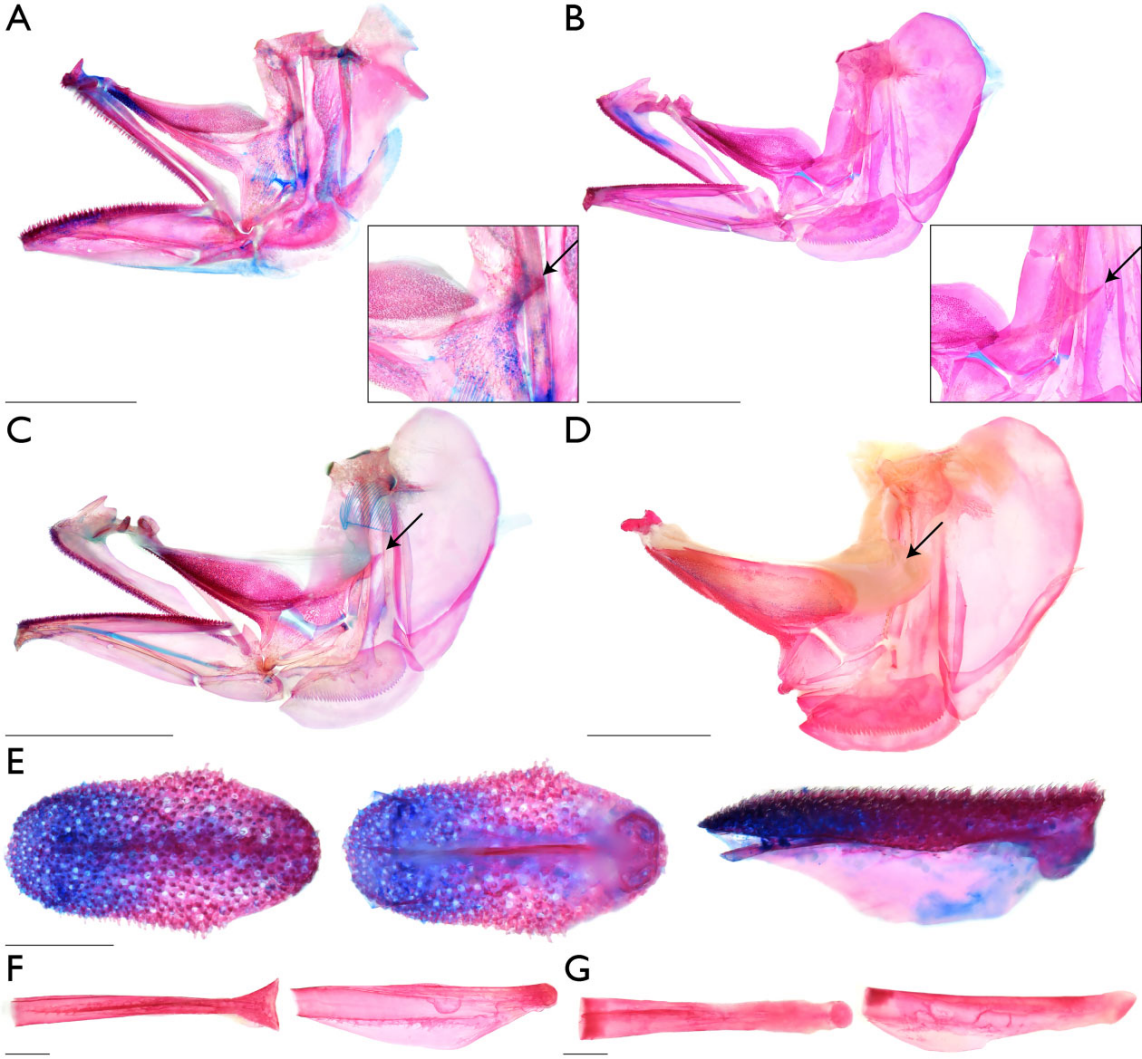
Morphological characters that support the Toxotoidei. Images of stained specimens under white or daylight LED light. Posterior margin of the metapterygoid with a flange-like extension that overlaps the hyomandibular ventral arm (character state 34_1_)—(A) *Leptobrama muelleri* (KUI 41406), box with arrow points to flange-like extension, medial view of suspensorium, scale bar 1 cm; (B) *Toxotes jaculatrix* (KUI 42174), box with arrow points to flange-like extension, medial view of suspensorium, scale bar 1 cm; (C) *T. chatareus* (UMMZ 236673), arrow points to pseudobranch tissue supported by flange-like extension, medial view of suspensorium, scale bar 1 cm; (D) *T. jaculatrix* (USNM 331437), arrow points to posterior portion of *adductor arcus palatini* supported by flange-like extension, medial view of suspensorium, scale bar 5 mm. Leptobramid basihyal for comparison with basihyal in toxotids (Fig. 5). The leftmost image shows the dorsal view of basihyal, the central image shows the ventral view of basihyal, rightmost image shows the left lateral view of basihyal (characters 40–45 and 49)—(E) *Leptobrama muelleri* (KUI 41406), scale bar 1 mm. Comparative basihyal keels in taxa outside the Toxotoidei—(F) *Forcipiger flavissimus* (USNM 166647), left image shows the ventral view of basihyal, right image shows the left lateral view of basihyal, scale bar 1 mm; (G) *Lethrinus harak* (USNM 290483), left image shows the ventral view of basihyal, right image shows the left lateral view of basihyal, scale bar 1 mm.

With the refined taxonomy of archerfishes, our 13-terminal analysis combined discrete morphological characters and genetic data to recover a hypothesis of relationships for the Toxotidae. Our combined analysis recovers a monophyletic Toxotidae within the Toxotoidei, sister to *Leptobrama muelleri* (Figs. 3 and 4) supported by 22 unambiguously optimized morphological character states and DNA-sequence data. The morphological study by Tominaga (1965) highlighted several similarities between the Leptobramidae and the Toxotidae, and beachsalmons have been recovered as the sister lineage of archerfishes in most DNA-based datasets when representatives of both families have been included (e.g., Betancur-R. et al., 2013a, 2013b, 2017; Harrington et al., 2016). The combined analysis of Girard et al. (2020) similarly recovered the Leptobramidae as the sister group to the Toxotidae supported by 14 morphological characters and DNA-sequence data. Five of the 14 characters supporting the Toxotoidei in Girard et al. (2020) also optimize in support of the suborder in this study (Fig. 4), including ankylosed endopterygoid teeth (their character state 40_1_, our character state 31_1_), posteriorly placed basihyal (their character state 63_1_, our character state 50_1_), ventral processes of coracoid and cleithrum distinctly separate (their character state 114_1_, our character state 66_1_), a vertically oriented first anal-fin pterygiophore (their character state 153_2_, our character state 83_1_), and an equal number or more anal-fin rays versus dorsal-fin rays (their character state 165_1_, our character state 89_1_). Among the remaining nine characters that optimize in support of the Toxotoidei in Girard et al. (2020) but do not optimize in support of the clade in our study, five were included and coded in our matrix (characters 13, 32, 64, 91, and 98) but did not optimize unambiguously in our analysis. This is likely due to differences in outgroup taxon sampling between the two studies. We also modified characters 135 and 136 from the study by Girard et al. (2020; our characters 75 and 76) to be more explicit in the homology of spines associated with dorsal-fin pterygiophores. We recover 15 additional characters that support a sister-group relationship between the Leptobramidae and the Toxotidae (Fig. 4). Nine of these 15 characters are described in this study (character 5, 9, 34, 41, 46, 49, 70, 74, and 95), and two characters are described in Allen (1978, 2004; our characters 73 and 78). The nine characters described in this study that support the monophyly of the Toxotoidei include: ventral aspect of parasphenoid containing a dorsally directed groove (character state 5_1_), posterior margin of the metapterygoid having a flange-like extension that overlaps the hyomandibular ventral arm (character state 34_1_), a basihyal that is ovoid in shape when viewed dorsally (character state 41_1_), presence of a ventral basihyal keel (character state 49_1_), and presence of a protuberance on the medioposterior margin of the cleithrum (character state 70_1_). In carangiform taxa examined outside of the Toxotoidei sampled in this study and in Girard et al. (2020), the posterior margin of the metapterygoid is largely simple. Some families (e.g., Centropomidae, Latidae) include species that possess a small rhomboid-shaped flange that may extend slightly into the margin of the hyomandibular ventral arm (see Greenwood et al., 1976). However, members of the Leptobramidae and the Toxotidae possess a flange-like extension of bone that originates from the posterior margin of the metapterygoid and extends posteriorly, completely overlapping the ventral arm of the hyomandibular and often overlapping elements of the opercular series (Fig. 7; Elshoud and Koomen, 1985, fig. 2). The flange typically possesses a broad base and tapers to a distal point. In both the Leptobramidae and the Toxotidae, the flange supports soft tissue leading to the pseudobranch (Fig. 7C), with this flange also serving as an attachment point for the posterior portion of the *adductor arcus palatini* in archerfishes (Fig. 7D). While we did not observe this flange in any other taxa sampled in this study beyond members of the Leptobramidae and the Toxotidae, a flange that is similar in position and extension is present in some members of the Lutjanidae (Johnson, 1980, figs. 29 and 30), but the flange in those taxa appear rounded distally compared to the tapering flange seen in members of the Toxotoidei. In addition to the metapterygoid flange, four characters that support the monophyly of the Toxotoidei relate to their oral structures. We find an indentation or groove in the ventral aspect of the parasphenoid and dorsal to the location of the palatal groove in members of the Toxotidae (Fig. 5A), as well as *Leptobrama muelleri* (character state 5_1_)*.* We also find three basihyal characters—basihyal ovoid in shape (character state 41_1_), basihyal bearing teeth (character state 43_1_), and presence of a basihyal keel (character state 49_1_)—that optimize in support of the Toxotoidei (Figs. 4, 5, and 7). Girard et al. (2020, figs. 7 and 8) showed a number basihyal shapes among carangiform taxa, which range from a slightly rounded basihyal (e.g., *Coryphaena*, *Oligoplites*) to a largely rectangular or hourglass shaped basihyal when viewed from the dorsally (*Achirus, Trachurus*). However, the basihyal of these taxa do not possess a keel-like extension along the ventral margin of the element. A distinct and ventrally directed keel on the basihyal was only found among members of the Toxotoidei (Figs. 5 and 7). The keel extended along most of the length of the basihyal in species of *Leptobrama, Protoxotes,* and *Toxotes*, becoming progressively deeper posteriorly. The keel ends in a cup-like dorsally directed indentation in all members of the Toxotoidei, which is where the first basibranchial inserts. Mok and Shen (1983) illustrated a similar basihyal keel in members of the Chaetodontidae (figs. 12P and 12Q) and Carpenter and Johnson (2002) noted a ventral basihyal keel in members of the Lethrinidae and *Pentapodus* (fig. 7A, their character state 35_1_). We examined the basihyal of *Forcipiger flavissimus*, *Heniochus diphreutes*, and *Lethrinus harak* (see Material examined) and, while these taxa do have basihyal keels, the keels differ from those in members of the Toxotoidei. The basihyal keel in chaetodontids extends along the entire ventral length of the basihyal, ascending as it reaches the posterior margin and does not end in a cup-like indentation (Fig. 7). Additionally, the keel in *Lethrinus* is restricted to the anterior part of the basihyal rather than continuing along the majority of the length as seen in members of the Toxotoidei (Fig. 7). Although keels can be found on the basihyal of fishes outside of the Toxotoidei, there are morphological differences between these keels compared to those in the Leptobramidae and the Toxotidae.

**Fig. 8:**
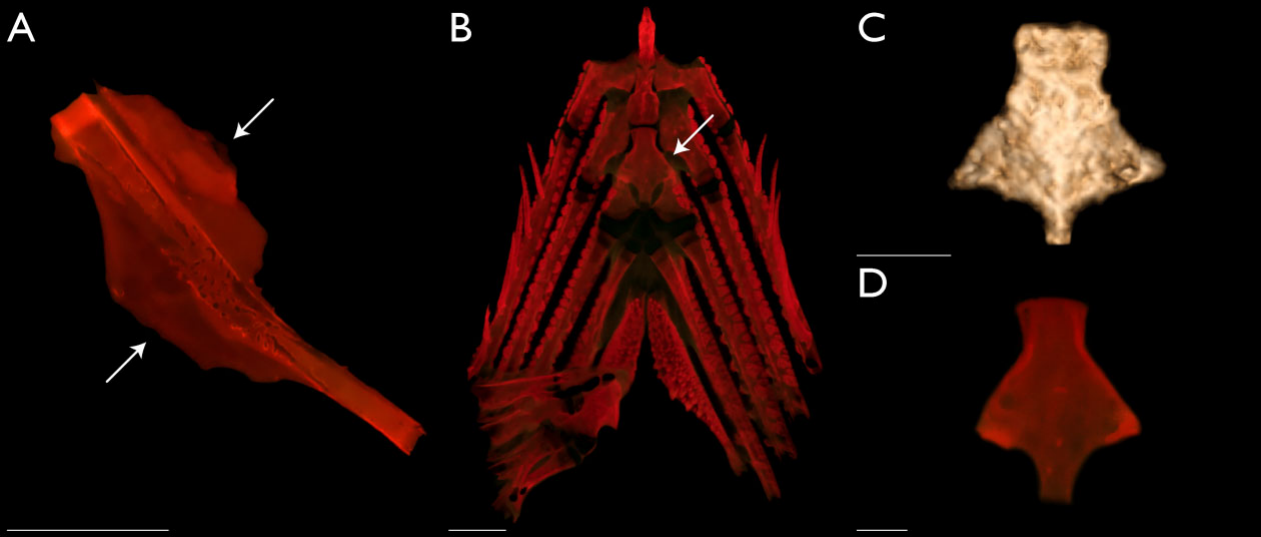
Morphological characters that support the Toxotidae. Images A, B, and D of cleared-and-stained specimens autofluorescing under royal blue LED light (see Materials and methods). Image C generated in 3D Slicer using µCT-scanned specimens. Lamellar expansions on the dorsal and ventral margins of the symplectic (character states 35_1_ and 36_1_)—(A) *Toxotes jaculatrix* (KUI 42174), arrows point to expansions, lateral view, scale bar 1 mm. Lateral expansions of the third basibranchial (character state 53_1_)—(B) *T. chatareus* (UMMZ 232688), arrow points to expansion, dorsal view, scale bar 1 mm; (C) *Protoxotes lorentzi* (USNM 406792), dorsal view, scale bar 1 cm; (D) *T. chatareus* (UMMZ 232688), dorsal view, scale bar 1 mm.

### Monophyly of the Toxotidae

We recover a monophyletic Toxotidae supported by 18 morphological characters (Figs. 3 and 4), including, but not limited to, the presence of a parasphenoid keel (character state 6_1_), maxillary largely uniform in depth throughout length (character state 24_1_), presence of flange-like lamellar expansions on the dorsal and ventral margins of the symplectic (character states 35_1_ and 36_1_; Fig. 8), and the lateral expansion of the third basibranchial (character state 53_1_; Fig. 8). Gushiken (1988) described a keel on the parasphenoid posterior to the vomer in members of the Carangoidei (i.e., Carangidae and Coryphaenidae) and illustrated a ventral expansion of the parasphenoid underneath the otic region of the neurocranium (his fig. 2). In this study, we find a similar ventral expansion of the parasphenoid in all members of the Toxotidae examined (character state 6_1_). The keel extends posteriorly, underneath the lateral arms of the parasphenoid, before becoming reduced approximately mid-way through the prootic. Aside from archerfishes, a parasphenoid keel is also present in *Lepomis cyanellus* among the taxa examined*.* Given that previous authors have noted the presence of a parasphenoid keel in other fishes, further investigation is needed to understand the distribution of this character across members of the Carangiformes and perch-like fishes more broadly. Another character supporting the monophyly of archerfishes relates to the depth of the posterior margin of the maxilla. In other members of the Carangiformes (e.g., Carangidae, Latidae, Polynemidae; Greenwood et al., 1976; Girard et al., 2020), the maxilla becomes dorsoventrally deeper in the posterior aspect of the element. A different condition exists in the archerfishes, in which the maxilla is largely uniform in its depth throughout its length (character state 24_1_). We also recover the presence of lamellar expansions on the symplectic in support of the monophyly of the Toxotidae (Fig. 8). The symplectic is often a rod-like element of the hyopalatine arch that joins the quadrate with the hyomandibular and articulates with the interhyal. Girard et al. (2020) described variation in the overall length of the element as well as lamellae extending from it. Two characters relating to the lamellar expansions on the dorsal and ventral margins of the symplectic (character states 35_1_ and 36_1_) optimize in support of the monophyly of the Toxotidae. The dorsal margin of the symplectic possesses a rounded lamellar expansion that extends dorsally, overlapping the ventral margin of the metapterygoid. The dorsal expansion begins approximately halfway up the length of the symplectic and continues to the dorsal end of the element. The ventral margin possesses a lamellar expansion of similar size, though it reaches its greatest length at the midline of the element. A similar dorsal lamellar expansion of the symplectic was not found outside of the Toxotidae among taxa sampled, but *Lates calcarifer* possesses a similar ventral expansion in both size and length to toxotids. The final character we highlight that supports toxotid monophyly is the shape of the third basibranchial when viewed dorsally (Fig. 8). In many carangiform and perch-like fishes, the third basibranchial is an elongate rectangular or hourglass shaped element (Girard et al., 2020). However, members of the Toxotidae sampled in this study have lateral expansions of the third basibranchial, giving the appearance of the capital letter “T” when viewed dorsally. The lateral expansions on the third basibranchial typically covers the dorsal margin of the third hypobranchial processes but otherwise does not interact with any other branchial elements.

### Intrarelationships of the Toxotidae

**Fig. 9:**
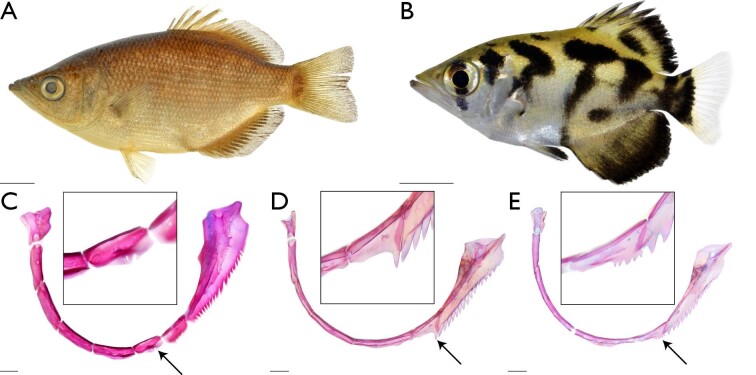
Morphological features that differ between *Protoxotes* and *Toxotes*. Images of whole or cleared-and-stained specimens under white or daylight LED light. Pigmentation absent along flank—(A) *P. lorentzi* (USNM 406792), left lateral view, scale bar 1 cm. Pigmentation present along flank—(B) *T. blythii* (KUI 42173), left lateral view, scale bar 1 cm. Serrations on the ventral margin of circumorbitals two and three (characters 14, 15, and 16)—(C) *P. lorentzi* (USNM 454833), arrow points to third circumorbital serrations, lateral view, cutout is close-up image of third circumorbital, scale bar 1 mm. Serrations on the ventral margin of circumorbital two (characters 14, 15, and 16)—(D) *T. chatareus* (UMMZ 236673); (E) *T. oligolepis* (SU 29567), arrow points to second circumorbital serration, lateral view, cutout is close-up image of second circumorbital, scale bar 1 mm.

**Fig. 10:**
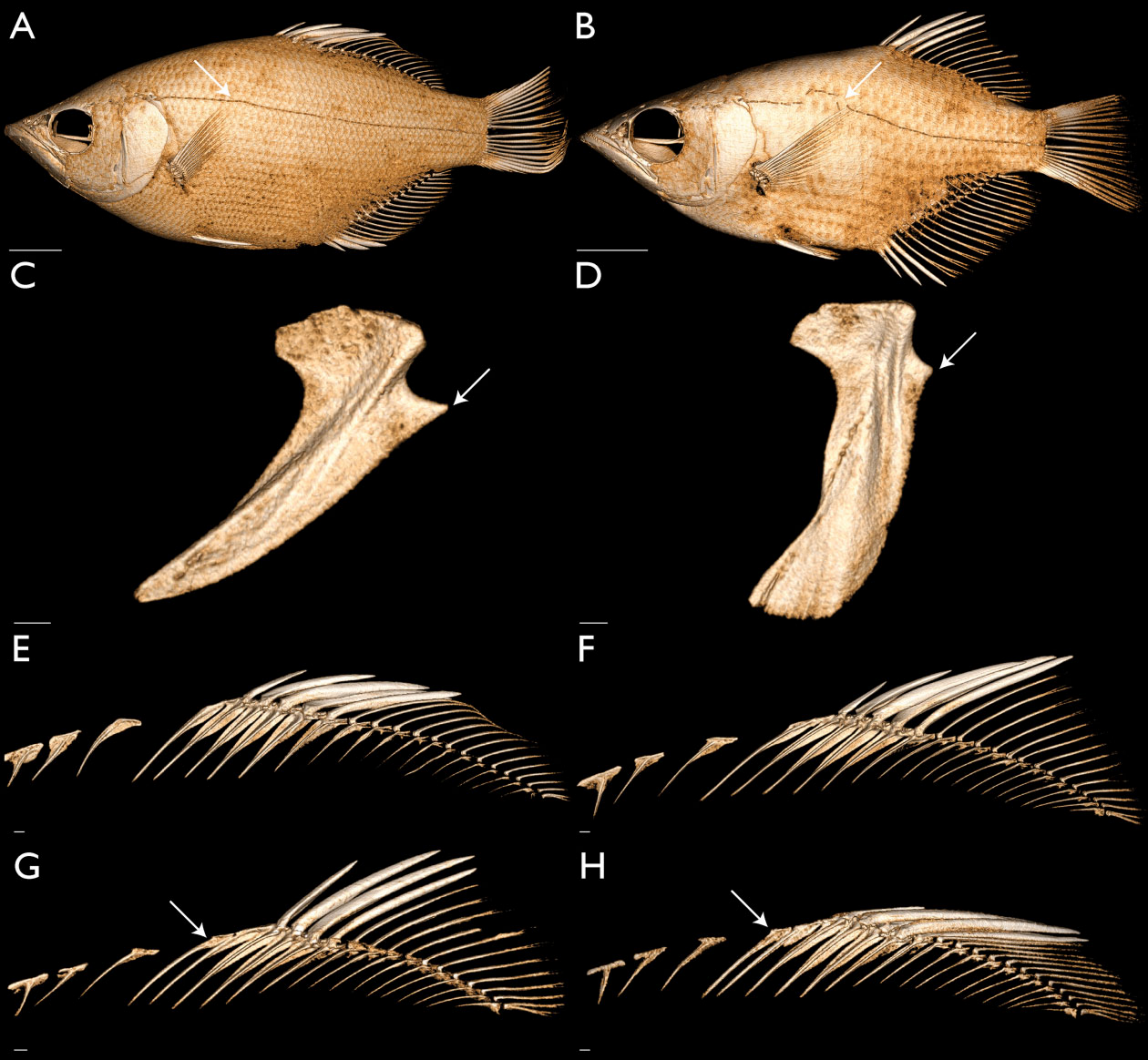
Morphological variation that supports the monophyly and relationships of *Toxotes* (see text). Images generated in 3D Slicer using µCT-scanned specimens. Lateral-line canal straight above pectoral fin (character state 99_0_) and confluent (character state 100_0_)—(A) *Protoxotes lorentzi* (USNM 406792), arrow points to lateral line, left lateral view, scale bar 1 cm. Lateral-line canal arched above pectoral fin (character state 99_1_) and interrupted (character state 100_1_)—(B) *T. jaculatrix* (SU 15516), arrow points to lateral line, left lateral view, scale bar 1 cm. Postcoracoid process strong with deep indentation dorsally (character state 69_1_)—(C) *P. lorentzi* (USNM 406792), left lateral view of left coracoid, scale bar 1 mm. Postcoracoid process weak with shallow indentation dorsally (character state 69_0_)—(D) *T. carpentariensis* (FMNH 63925), left lateral view of left coracoid, scale bar 1 cm. Absence of a spineless proximal-middle dorsal pterygiophore (character state 76_0_)—(E) *P. lorentzi* (USNM 406792), left lateral view of left coracoid, scale bar 1 mm; (F) *T. chatareus* (CAS 94720), left lateral view of left coracoid, scale bar 1 mm. Presence of a spineless proximal-middle dorsal pterygiophore (character state 76_1_)—(G) *T. jaculatrix* (SU 15516), arrow points to pterygiophore, left lateral view of left coracoid, scale bar 1 mm; (H) *T. oligolepis* (SU 29567), arrow points to pterygiophore, left lateral view of left coracoid, scale bar 1 mm.

Our combined analysis of the 13-terminal dataset recovers *P. lorentzi* as the sister group to the remaining members of the Toxotidae, with 11 characters supporting the monophyly of *Toxotes* (Figs. 3 and 4). Although this taxon had yet to be included in a phylogenetic analysis, Weber and de Beaufort (1936) and Whitley (1950:244) suggested this species is the “most primitive” archerfish based on a combination of characters (see above), with Whitley (1950) describing the genus *Protoxotes*. However, subsequent authors (Taylor, 1964; Allen, 1978) continued to place the taxon in *Toxotes*. We corroborate the findings of Weber and de Beaufort (1936) and Whitley (1950) regarding lateral-line shape, flank pigmentation, and scale size for this taxon (Figs. 9 and 10). We additionally find a set of characters that diagnose *P. lorentzi*, including serrations on the ventral aspect of the third circumorbital (character 16_1_) and a short pelvic axillary scale that extends no longer than ⅓ the length of the pelvic spine (character 97_0_). While all archerfishes examined in this study exhibit serrations along the lachrymal and second circumorbital, *P. lorentzi* possesses multiple serrations along the ventral margin of the third circumorbital. This condition is different from other archerfishes, which lack serrations on the third circumorbital (Fig. 9). This character, along with others in this study, and those highlighted by Weber and de Beaufort (1936) and Whitley (1950), show the number of differences between *P. lorentzi* and other species of archerfishes.

The monophyly of a restricted *Toxotes* is supported by 11 morphological characters. These include a weak postcoracoid process with a shallow indentation (character state 69_0_; Fig. 10) and an interrupted lateral line (character state 100_1_; Fig. 10). Positioned on the dorsoposterior angle of the coracoid and typically directly underneath the ventralmost pectoral radial, the postcoracoid process varies in presence and absence as well as shape among carangiform fishes as noted by Girard et al. (2020, characters 116 and 117). While all archerfishes sampled in this study have a postcoracoid process, the overall shape of this process varies, with species of *Toxotes* possessing a weak process that extends a small to moderate amount from the coracoid (Fig. 10). Taxa with a weak process also have a shallow indentation dorsal to the process (Fig. 10D). However, *P. lorentzi* has a strong postcoracoid process with a deep dorsal indentation (Fig. 10C) similar to other carangiform and non-carangiform taxa sampled. A weak postcoracoid process with a shallow indentation (character state 69_0_), along with 10 other morphological characters, support the monophyly of *Toxotes.*

Within *Toxotes*, we recover *T. sundaicus* as the sister taxon to the remaining species in the genus (Figs. 3 and 4). However, this placement is based solely on morphological data because specimens of this taxon were not available for sequencing. *Toxotes sundaicus,* which is restricted to Borneo and Sumatra, is similar morphologically to the widespread *T. chatareus*. We do not currently dispute the validity of this taxon, but encourage subsequent researchers to sample *T. sundaicus* using DNA-sequence data for comparison and analysis. The next clade of archerfishes we recover in our combined analysis is *T.chatareus* sister to a clade consisting of *T. blythii, T. carpentariensis*, *T. jaculatrix, T. kimberleyensis,* and *T. oligolepis*. This clade is supported by three morphological characters, including a large oculomotor foramen (character state 4_1_). We observed a foramen of various size and relative position in the anterior aspect of the prootic in many of the taxa sampled. We interpret this foramen to be the oculomotor foramen based on the illustrations in Patterson (1970, Fig. 5B), as it is positioned between the lateral arms of the basisphenoid and the prootic. In addition to the differential presence and absence of the foramen, its size varies among the taxa sampled. A large oculomotor foramen (character state 4_1_) optimizes in support of a clade including *T. blythii, T. carpentariensis, T. chatareus, T. jaculatrix, T. kimberleyensis,* and *T. oligolepis*. Among the taxa examined outside of the Toxotidae, only *Perca flavescens* possesses similar state with respect to the size and position of this foramen. While further investigation into the homology and phylogenetic significance of this foramen is needed, a large oculomotor foramen supports the monophyly of *T. blythii, T. carpentariensis, T. chatareus, T. jaculatrix, T. kimberleyensis,* and *T. oligolepis*.

The next clade we recover in our 13-terminal analysis includes *T.carpentariensis* sister to a clade of *T. blythii*, *T. jaculatrix, T. kimberleyensis,* and *T. oligolepis* (Figs. 3 and 4). This clade is supported by only DNA data, as no morphological characters optimized unambiguously on this node. An ACCTRAN optimization finds two characters in support of this node, with one being the presence of two or more serrations on the ventral margin of circumorbital two (character state 15_0_). While this character may support the relationship of *T. blythii, T. carpentariensis, T. jaculatrix, T. kimberleyensis,* and *T. oligolepis*, this character state also occurs in *Protoxotes* and *Lates* among taxa sampled in this study. We interpret this character to be symplesiomorphic and further investigations are needed to better understand the relationship among *T. blythii, T. carpentariensis, T. jaculatrix, T. kimberleyensis,* and *T. oligolepis.*

The next clade we recover in our 13-terminal analysis includes *T. blythii* as the sister taxon to a clade of *T. jaculatrix, T. kimberleyensis,* and *T. oligolepis,* which is supported by one morphological character and DNA-sequence data (Figs. 3 and 4). The character that optimizes onto this node is the unequal dorsal and ventral process-lengths on the lateral gill raker associated with the junction between the first epibranchial and first ceratobranchial (character state 60_1_). A lateral gill raker may be present and associated with the junction between the first epibranchial and first ceratobranchial. When present, this gill raker possesses both dorsal and ventral processes that emerge from the proximal aspect of the gill raker and interact with the epibranchial or ceratobranchial, respectively. The overall length of these processes is predominantly equal among taxa sampled in this study, except for *T. blythii, T. jaculatrix, T. kimberleyensis,* and *T. oligolepis,* where the dorsal process is reduced in length when compared to the ventral process*.*

We recover *T. kimberleyensis* as the sister group to a clade of *T. jaculatrix* and *T. oligolepis* in our combined analysis (Figs. 3 and 4). This clade is supported by two morphological characters, including between 25 and 32 lateral-line scales (character state 98_0_). *Toxotes jaculatrix*, *T. kimberleyensis,* and *T. oligolepis* possess the fewest number of lateral-line scales within the family, along with some specimens of *T. carpentariensis* and *T. chatareus* (see Allen, 1978, 2004; Kottelat and Tan, 2018)*.* Within this clade, *T. jaculatrix* and *T. oligolepis* are recovered as sister taxa that are supported by five morphological characters, including the basihyal obtaining greatest width medioposteriorly (character state 42_0_), presence of a spineless proximal-middle dorsal pterygiophore (character state 76_1_, Fig. 10), and body elongate or largely elongate (character state 85_0_). As noted above, the basihyal is similarly shaped across species of *Toxotes.* However, the basihyal in *T. jaculatrix* and *T. oligolepis* (Fig. 5) is slightly different from other toxotids, as it obtains its greatest width within the posterior ⅓ of the element's length. Another character in support of this clade is presence of a spineless proximal-middle dorsal pterygiophore (character state 76_1_). While modifications to the dorsal fin have occurred in the evolutionary history of fishes, archerfishes possess a serial arrangement between dorsal-fin spines and pterygiophores, resulting in only one spine associating with each proximal-middle pterygiophore of the dorsal fin (Fig. 10E and 10F). However, *T. jaculatrix* and *T. oligolepis* exhibit a spineless proximal-middle pterygiophore (Fig. 10G and 10H) anterior to the first proximal-middle pterygiophore that bears a dorsal spine. Spineless proximal-middle pterygiophores were not observed in any other species of *Toxotes* examined.

### Archerfish oral structures and the shooting mechanism

**Fig. 11:**
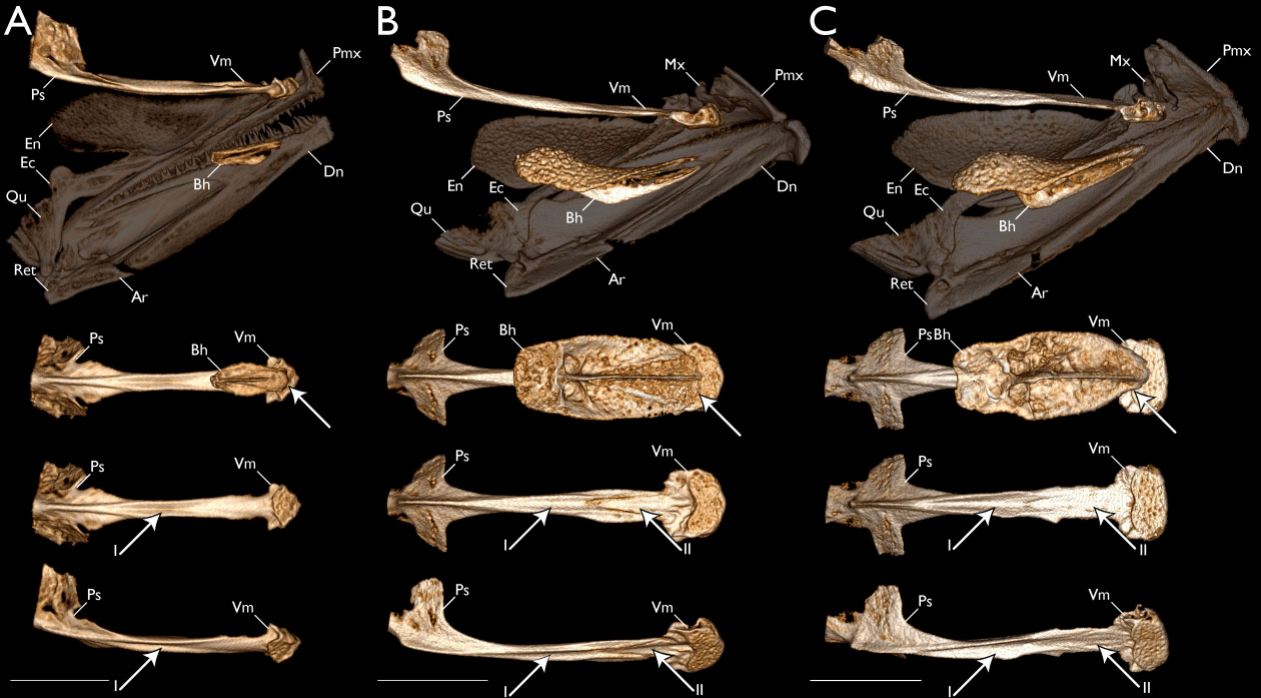
Osteology and orientation of oral structures in Toxotoidei. Images generated in 3D Slicer using µCT-scanned specimens. Position of basihyal relative to groove in parasphenoid and vomerine tooth plate at resting position—first row, right lateral view. Basihyal position relative to groove in parasphenoid and vomerine tooth plate at resting position—second row, arrow points to tip of basihyal overlapping vomerine tooth plate, ventral view. Groove in ventral margin of parasphenoid, vomer, and mesial indentation of vomerine tooth plate—third and fourth rows, arrow I points to ventral groove in parasphenoid, arrow II points to ventral groove in vomer, ventral view. (A) *Leptobrama pectoralis* (QM I.27586). (B) *Protoxotes lorentzi* (USNM 406792). (C) *Toxotes carpentariensis* (FMNH 63925); scale bars all 5 mm. Abbreviations: Ar, articular; Bh, basihyal; Dn, dentary; Ec, ectopterygoid; En, endopterygoid; Mx, maxilla; Pmx, premaxilla; Ps, parasphenoid truncated posterior to lateral arms; Ret, retroarticular; Qu, quadrate; Vm, vomer.

There are two hypotheses about the formation of the water jet archerfishes use to attack prey—the blowpipe and the pressure tank. That is, either these fishes rapidly change the volume of the buccal cavity, ejecting a stream of water, or pressurize the cavity until a valve releases the pressure, allowing the shot to emerge. Elshoud and Koomen (1985) found the blowpipe implausible because they found the ventral margin of the parasphenoid nearly straight, the groove in the soft tissue directed at the posterior margin of the vomer, and mismatch between the dorsal margin of the basihyal and ventral margin of the parasphenoid. We disagree with the finding that the ventral margin of the parasphenoid is nearly straight—there is a dorsally directed groove in not only soft tissue (i.e., palatal groove), but also in hard tissue, with the ventral aspect of the parasphenoid and vomer having grooves (Figs. 5 and 11). While we do not disagree that the hard- and soft-tissue grooves are directed at the vomerine tooth plate, we do not think this impacts the plausibility of the blowpipe hypothesis. A close examination of the soft tissue near the vomer shows a smooth ventrally sloping margin that leads toward the tooth plate (Figs. 5A and 11). This slope ends near the concave posterior margin of the vomerine tooth plate, allowing for a largely flush margin between the palatal groove and the ventral margin of the vomer (Fig. 5A). Elshoud and Koomen (1985) also mention that there is a mismatch between the palatal groove and dorsal margin of the basihyal. However, when taking the orientation and dorsal topology of the basihyal into account, we find the most-elevated portion of the basihyal lies beneath the most pronounced portion of the parasphenoid groove (Fig. 11). Where the anterior portion of the basihyal overlaps the posterior ⅓ of the vomerine tooth plate, the basihyal is mesially concave, allowing for a small gap to be formed between the palatal groove, slope of the vomer, concave posterior margin of the vomerine tooth plate, and anteriorly concave dorsal margin of the basihyal (Figs. 5 and 11). These hard and soft-tissue structures support the plausibility of the blowpipe hypothesis. However, we cannot support or refute that the oral valves play a role in the shooting apparatus. We did observe an anterior and mesial aperture in both the upper and lower oral valves of *P. lorentzi,T.chatareus,* and *T. jaculatrix* (Fig. 5A), which is a different condition to the continuous oral valves found in *Lates, Leptobrama,* and *Nematistius*. However, the study by Timmermans and Souren (2004) found that the shape of the shot is horizontally flat in cross section immediately after exiting from the archerfish mouth. They go on to state that this shape indicates that the shot passes over a horizontally flat aperture, not through a narrow aperture of the oral valves, before exiting the oral cavity. Other soft-tissue structures, such as those surrounding the basihyal, may also play a role in projecting water. The tissue associated with the basihyal may provide a gasket-like seal to prevent water from moving laterally or posteriorly when the basihyal is compressed against the palatal groove. Further studies are needed to test if the variation in basihyal shape and cartilaginous cap position, particularly the differences between *Protoxotes* and *Toxotes*, as well as if the oral valves or soft tissue surrounding the basihyal have any impact on the formation and projection of an archerfish shot.

### Evolution of archerfish oral structures

In examining the osteology of the archerfish sister group, the Leptobramidae, we were surprised to find it has relevant shooting features within the oral cavity. There is a soft-tissue palatal groove between the endopterygoid tooth patches, a groove in the ventral margin of the parasphenoid, and an ovoid basihyal with a ventral keel (Figs. 7 and 11). We also find the posterior flange associated with the metapterygoid in both leptobramids and toxotids, which in archerfishes supports the *adductor arcus palatini* and plays a role in the shooting mechanism (Fig. 7; Milburn and Alexander, 1976). However, we do not consider it likely that leptobramids shoot like archerfishes; the leptobramid basihyal lacks the posterior elevation (our character 40; see Figs. 5, 7, and 11) and is smaller than the basihyal of archerfishes. Furthermore, the ventral margin of the vomer and posterior margin of the vomerine tooth plate are convex in leptobramids, rather than possessing a groove or concavity seen in archerfishes (Fig. 11). Despite these differences, the resulting phylogeny and the characters suggest that the palatal groove, parasphenoid groove, keeled basihyal, and metapterygoid flange were present in the ancestors of the Toxotoidei lineage and are examples of exapted or co-opted traits for the pressurization of water through and out of the oral cavity in archerfishes. Exaptation is defined as the co-option of a character or trait toward a use that is different from the one it was selected (see Gould and Vrba, 1982). Species of *Leptobrama* and *Toxotes* feed on primarily arthropods and fishes (Goutham-Bharathi et al., 2013; Kembaren and Taufik, 2020). However, archerfishes consume a greater proportion of crustaceans and insects (Simon and Mazlan, 2010) and primarily live in freshwater habitats where encountering these terrestrial and benthic prey is a common occurrence. The co-option of the grooves in hard and soft tissue, posterior metapterygoid flange, keeled basihyal, and associated adaptive changes to the dorsal margin of the basihyal, would allow for archerfishes to take advantage of these abundant energy resources in terrestrial and buried prey. Additional features, such as improvements in vision, cognition, and swimming ability, are necessary for successful shooting behavior to be executed successfully and further comparisons between leptobramids and toxotids are needed to understand the evolution of these features more broadly.

We found that the oral structures of archerfishes are conserved across species, with some variation in the shape of the basihyal and the position of the rostral cartilaginous cap on the basihyal. We also found the oral structures of archerfishes agree with earlier descriptions of archerfish anatomy and support the original blowpipe hypothesis by Smith (1936, 1945) and Myers (1952). However, other soft-tissue oral structures, including the oral valves highlighted by Elshoud and Koomen (1985), may function in the mechanism. The combined morphological and molecular phylogeny presented provides an opportunity to investigate the evolution of the shooting structures associated with archerfish hunting behavior, which are the result of exaptation of oral structures in the leptobramid-toxotid ancestral lineage. We hope that our findings will inform delimitation of additional species and understanding the evolution of archerfishes. We encourage subsequent authors to generate both morphology-based and DNA-based characters when delimiting species and refining the current species of toxotids so additional evolutionary hypotheses can be produced and tested.

## Material examined

In the following section, specimens examined as prepared cleared-and-stained specimens are denoted “CS”; specimens examined as prepared stained specimens but omit the steps involving trypsin are denoted “SM”; specimens examined as whole ethanol specimens are denoted “ET” with an “*” indicating one or more specimen(s) were µCT scanned or x-rayed; specimens examined as dry osteological preparations and skeletons are denoted “SK.” Image stacks of µCT-scanned specimens have been uploaded to MorphoSource, with associated media identifiers listed in brackets that follow the preparation types. Following the listing of each taxon, an approximate standard length (SL) or range of SL are listed for the specimens examined.

### Non-carangiform taxa

***Forcipiger flavissimus***: USNM 166647 (1 CS), 76 mm SL.

***Heniochus diphreutes***: USNM 439405 (1 CS), 57 mm SL.

***Lethrinus harak***: USNM 290483 (1 CS), 85 mm SL.

***Lepomis cyanellus***: KUI 15960 (6 CS), 43–62 mm SL.

***Perca flavescens***: KUI 16973 (2 CS), 47–60 mm SL.

### Carangiform taxa

***Achirus lineatus***: FMNH 113137 (2 CS) 56–58 mm SL.

***Coryphaena hippurus***: FMNH 48561 (2 CS) 73–74 mm SL.

***Lates calcarifer***: AMNH 37839 (1 CS; 1 ET) 82–85 mm SL; USNM 367101 (1 ET) 68 mm SL.

***Leptobrama muelleri***: KUI 41406 (1 CS) 113 mm SL; UW 7204 (1 ET), 270 mm SL.

***Leptobrama pectoralis***: AMNH 219223 (1 SK) 268 mm SL; AMNH 219224 (1 SK) 242 mm SL; QM I.27586 (1 CS; 2 ET* [000415985]), 117–119 mm SL.

***Nematistius pectoralis***: ANSP 148654 (1 CS) 190 mm SL; SIO 12–3085 (1 CS) 78 mm SL; USNM 81985 (1 ET) 66 mm SL.

***Oligoplites saurus***: KUI 17205 (1 CS; 1 ET) 56–62 mm SL.

***Trachurus trachurus***: KUI 19964 (2 CS; 1 ET) 45–70 mm SL.

### Toxotidae

***Protoxotes lorentzi***: USNM 406792 (8 ET* [000415974]) 78–95 mm SL; USNM 454833 (1 CS) 79 mm SL.

***Toxotes blythii***: KUI 42173 (1 CS; 1 ET* [000415768]) 36–46 mm SL; KUI 42698 (3 ET) 41–45 mm SL; LSUMZ 17019 (2 ET) 74–81 mm SL.

***Toxotes carpentariensis***: CSIRO A3722 (1 ET* [000415810]) 78 mm SL; FMNH 63925 (3 ET* [000415956]) 57–87 mm SL; LSUMZ 17586 (2 ET* [000415830]) 51–57 mm SL; LSUMZ 17587 (3 ET) 21–23 mm SL; USNM 173503 (11 ET* [000415961]) 46–68 mm SL; USNM 454834 (1 CS) 53 mm SL.

***Toxotes chatareus***: CAS 93958 (2 ET) 118–127 mm SL; CAS 94720 (1 ET* [000415996]) 93 mm SL; CAS 94737 (2 ET) 94–96 mm SL; CAS 97101 (1 ET) 71 mm SL; CAS 97077 (2 ET) 40–46 mm SL; FMNH 68790 (1 ET* [000415844]) 93 mm SL**;** KUI 42697 (1 CS; 1 SM) 55–71 mm SL; KUI 42699 (1 ET) 58 mm SL; OS 4790 (1 ET) 98 mm SL; UF 173178 (3 ET* [000415834]) 48–53 mm SL; UF 188329 (2 ET) 21–31 mm SL; UF 188548 (3 ET) 17–30 mm SL; UF 241575 (1 ET) 83 mm SL; UMMZ 232552 (2 ET* [000415826]) 66–78 mm SL; UMMZ 232688 (1 CS; 2 ET* [000415848]) 43–67 mm SL; UMMZ 234546 (1 ET) 69 mm SL; UMMZ 236673 (1 CS; 3 ET* [000415822]) 66–94 mm SL; UMMZ 241226 (4 ET* [000415852]) 45–50 mm SL; UMMZ 241605 (3 ET* [000415862]) 51–89 mm SL; USNM 364588 (13 ET) 15–38 mm SL.

***Toxotes jaculatrix***: CAS 206638 (2 ET) 37–48 mm SL; CAS 206639 (3 ET) 54–85 mm SL; CAS 206640 (4 ET) 56–145 mm SL; CAS 206641 (6 ET) 18–46 mm SL; CAS 206642 (2 ET) 142–145 mm SL; CAS 206643 (1 ET) 138 mm SL; FMNH 69510 (3 CS, 4 ET) 61–80 mm SL; KUI 42174 (1 CS) 60 mm SL; SU 15516 (9 ET* [000415806]) 26–65 mm SL; USNM 331437 (1 SM; 9 ET) 30–75 mm SL; USNM 331439 (1 ET) 93 mm SL; USNM 441787 (2 CS) 28–52 mm SL.

***Toxotes kimberleyensis***: AMS I.42570–001 paratype (1 ET*) 107 mm SL; WAM P.2620–001 (1 ET* [000415816]) 61 mm SL.

***Toxotes oligolepis***: SU 29567 (1 CS; 1 ET* [000415840]) 61–72 mm SL.

***Toxotes sundaicus***: ZRC 42270 paratype (2 ET* [000415979]) 81–88 mm SL.

## Supplementary Material

obac013_Supplemental_Files

## Data Availability

The data underlying this article are available in the article, the online supplementary data, GenBank, and MorphoSource (see supplementary data). The authors declare no competing interests.
