# Supplementary material for: Phylogenetics of Archerfishes (Toxotidae) and Evolution of the Toxotid Shooting Apparatus
Source: Integr Org Biol. 2022 Mar 21;4(1):obac013. doi: 10.1093/iob/obac013 (PMC9259087; doi:10.1093/iob/obac013)
Supplement: obac013_Supplemental_Files [file obac013_supplemental_files.zip › Supplementary_file_1_rev.docx]

## Supplementary File 1 - Descriptions and States for the Characters Examined

In the following characters descriptions, we will use or modify a number of characters that are previously discussed or described by McAllister (1968), Patterson (1970), Miller and Lea (1972), Greenwood (1976), Allen (1978, 2004), Mok and Shen (1983), Webb (1989), Munroe (1992), Westneat (1993), Shinohara (1994), Ross (2001), Morgan and Gill (2006), Springer and Smith-Vaniz (2008), Pethiyagoda and Gill (2013), Kimura et al. (2016), Märss et al. (2017), Kottelat and Tan (2018), Rojo (2018), and Girard et al. (2020; citation information available in the References section of the main text). Further, a subset of these characters have long histories within the literature. In an effort to not repeat this information, we direct readers to the descriptions and historical use of these characters discussed in Girard et al. (2020). All multistate characters are unordered.

### Neurocranium

1. Accessory nasal ossifications (Girard et al., 2020 character 3):

Among taxa in this study, accessory nasal ossifications are present in *Nematistius pectoralis* and all members of the Toxotidae.

(1_0_)=Accessory nasal ossifications absent.

(1_1_)=Accessory nasal ossifications present.

2. Accessory nasal ossifications proximity to nasal:

As noted above, a subset of taxa in this study possess accessory ossifications associated with the nasal. When present, the proximity of the accessory ossification to the nasal varies from being closely applied to distantly separated. Taxa with accessory nasal ossifications that are closely applied to the nasal include all members of the Toxotidae. Taxa that do not possess accessory nasal ossifications (character 1) are coded as inapplicable (-) for this character.

(2_0_)=Accessory nasal ossifications distantly separated from nasal element.

(2_1_)=Accessory nasal ossifications closely applied to nasal element.

3. Foramen associated with prootic:

Viewing through the orbit, the anterior aspect of the prootic differentially possesses a foramen of various size and position relative to the meningost arms of the basisphenoid among taxa in this study. Based on the illustrations in Patterson (1970; figure 5B), we interpret the foramen to be the oculomotor foramen, which is positioned between the lateral arms of the basisphenoid and the prootic. All but one taxon in this study, *Lates calcarifer,* possess an oculomotor foramen associated with the prootic.

(3_0_)=Oculomotor foramen associated with prootic.

(3_1_)=Oculomotor foramen not associated with prootic.

4. Size of oculomotor foramen associated with prootic:

As noted above, most taxa in this study possess a foramen associated with the prootic. The size of this foramen varies, from a small foramen with discrete margins, to a large foramen with or without discrete margins. Taxa with a small oculomotor foramen with discrete margins include *Lepomis cyanellus, Leptobrama muelleri, Nematistius pectoralis, Protoxotes lorentzi,* and *T. sundaicus*. *Lates calcarifer,* which does not exhibit an oculomotor foramen (character 3), is coded as inapplicable (-) for this character.

(4_0_)=Oculomotor foramen small with discrete margins.

(4_1_)=Oculomotor foramen large, with or without discrete margins.

5. Ventral aspect of the parasphenoid:

Among the taxa in this study, many possess a dorsally directed groove on the ventral aspect of the parasphenoid and on the roof of the oral cavity. In outgroup taxa, the ventral aspect of the parasphenoid is made of flattened or smooth bone and lacked a groove. Taxa with a ventral groove in the parasphenoid include *Leptobrama muelleri* and all members of the Toxotidae.

(5_0_)=Ventral aspect of parasphenoid made of flattened or smooth bone.

(5_1_)=Ventral aspect of parasphenoid contains dorsally directed groove.

6. Parasphenoid keel:

On the ventral aspect of parasphenoid and ventral to the otic region of the neurocranium, the parasphenoid is either made of flattened bone or possesses a distinct ventrally-directed keel. This parasphenoid keel is present in *Lepomis cyanellus* and all members of the Toxotidae.

(6_0_)=Parasphenoid keel absent.

(6_1_)=Parasphenoid keel present.

7. Position of supraoccipital crest:

While all taxa in this study possess a supraoccipital, the position of the supraoccipital crest varies among them. The supraoccipital crest is either restricted to the posterior aspect of the occipital region of the neurocranium or extended rostrally towards the frontal. Taxa with a supraoccipital crest that is restricted to the posterior aspect of the occipital region of the neurocranium include *Perca flavescens* and all members of the Toxotidae.

(7_0_)=Supraoccipital crest extends rostrally towards frontal.

(7_1_)=Supraoccipital crest restricted to the posterior aspect of the occipital region of the neurocranium.

8. Parietal crest:

The parietal crest is a lamellar extension of bone that extends dorsolaterally from the parietal. Among taxa in this study, many do not exhibit a parietal crest. They include *Perca flavescens, Toxotes carpentariensis, T. chatareus, T. jaculatrix, T. kimberleyensis, T. oligolepis,* and *T. sundaicus*.

(8_0_)=Parietal crest present.

(8_1_)=Parietal crest absent.

9. Supraorbital commissure:

The supraorbital canal passes through the nasal and frontal bones in most fishes (Webb, 1989). Additionally, one or more commissure canals, such as the supratemporal commissure, are often present and link the sensory canals on the two sides of the neurocranium when complete (Webb, 1989). Among taxa in this study, most lacked a supraorbital commissure canal. Taxa that do not possess a supraorbital commissure canal include *Leptobrama muelleri, Perca flavescens,* and all members of the Toxotidae.

(9_0_)=Supraorbital commissure canal present.

(9_1_)=Supraorbital commissure canal absent.

10. Groove in dorsal margin of epiotic (based on Mok and Shen, 1983):

In their study of squammapines, Mok and Shen (1983) note variation in the arrangement and interaction between the pectoral girdle and neurocranium among the taxa in their study. Variation is also noted with respect to the dorsal margin of the epiotic, where the anterior process of the posttemporal either interacts with a groove in the dorsal margin of the epiotic or lays on top of a groove-less epiotic. Mok and Shen (1983) note that no epiotic groove is present in *Toxotes chatareus*, and the anteriorly directed posttemporal process firmly articulates with the epiotic. In applying this character to the taxa in this study, a groove in the dorsal margin of the epiotic is present in relatively few taxa. Taxa without a groove in the dorsal margin of the epiotic include all members of *Toxotes*. Note that this character is best observed by removing the posttemporal, which covers the dorsal margin of the epiotic.

(10_0_)=Dorsal margin of epiotic with distinct groove.

(10_1_)=Dorsal margin of epiotic without groove.

### Circumorbitals

11. Lamellar expansion of circumorbitals:

The circumorbitals are often shaped as thin, tube-like elements, but they can become ventrally or caudally expanded by lamellar bone in some taxa. Among the taxa in this study, one or more circumorbital elements are expanded by lamellar bone in some taxa, including *Lates calcarifer, Leptobrama muelleri, Nematistius pectoralis,* and all members of the Toxotidae. All other taxa possess tube-like circumorbitals.

(11_0_)=All circumorbitals thin and tube like.

(11_1_)=One or more circumorbitals expanded by lamellar bone.

12. Extent of lamellar circumorbital expansion:

As noted above, variation is present in the expansion of circumorbitals among the taxa in this study. When a lamellar expansion of the circumorbitals is present, variation is also present in which circumorbitals possess the expansion(s). Taxa possess expansion of only the second circumorbital, expansion of the second and third circumorbitals, or expansion of all circumorbitals. Taxa with second and third circumorbital expansions include *Lates calcarifer, Protoxotes lorentzi,* and *Toxotes jaculatrix*. Taxa with expansions on all circumorbitals include *Leptobrama muelleri*. Taxa that do not possesses expanded circumorbitals (character 11) are coded as inapplicable (-) for this character.

(12_0_)=Lamellar circumorbital expansion on second element only.

(12_1_)=Lamellar circumorbital expansion on second and third elements only.

(12_2_)=Lamellar circumorbital expansion on all circumorbitals.

13. Serrations on ventral margin of lachrymal (Girard et al., 2020 character 15):

Among the taxa in this study, taxa with serrations on the ventral margin of the lachrymal include *Lates calcarifer, Lepomis cyanellus, Leptobrama muelleri,* and all members of the Toxotidae.

(13_0_)=Ventral margin of lachrymal without serrations.

(13_1_)=Ventral margin of lachrymal serrated.

14. Serrations on ventral margin of second circumorbital:

As noted above, the lachrymal may possess ventral serrations. In some taxa, serrations are also present on the ventral margin of the second circumorbital element. Taxa with serrations on the second circumorbital element include *Lates calcarifer, Leptobrama muelleri,* and all members of the Toxotidae.

(14_0_)=Ventral margin of second circumorbital smooth

(14_1_)=Ventral margin of second circumorbital with one or more serrations.

15. Number of serrations on ventral margin of second circumorbital:

As noted above, the second circumorbital differentially possesses one or more serrations on the ventral margin. Variation is also present in the number of serrations found along the ventral margin of the second circumorbital element among the taxa in this study. Taxa with two or fewer serrations on the ventral margin of the second circumorbital include *Toxotes chatareus* and *T. sundaicus*. Taxa that lack serrations on the ventral margin of the second circumorbital (character 14) are coded as inapplicable (-) for this character.

(15_0_)=Three or more serrations on the ventral margin of the second circumorbital.

(15_1_)=Two or fewer serrations on the ventral margin of the second circumorbital.

16. Third circumorbital serrated:

As noted above, the lachrymal and second circumorbital may possess ventral serrations. In some taxa, serrations are also present on the ventral margin of the third circumorbital element. Taxa with serrations on the third circumorbital element include *Lates calcarifer* and *Protoxotes lorentzi*.

(16_0_)=Ventral margin of third circumorbital smooth.

(16_1_)=Ventral margin of third circumorbital with one or more serrations.

17. Number of circumorbitals including dermosphenotic (Girard et al., 2020 character 16):

Among the taxa in this study, those with seven or more circumorbitals include *Lepomis cyanellus, Leptobrama muelleri, Nematistius pectoralis,* and all members of the Toxotidae.

(17_0_)=Six or fewer circumorbitals.

(17_1_)=Seven or more circumorbitals.

18. Suborbital shelf (modified from Girard et al., 2020 character 18):

Among the taxa in this study, a suborbital shelf is present in *Lates calcarifer, Leptobrama muelleri,* and *Nematistius* *pectoralis*.

(18_0_)=Suborbital shelf present.

(18_1_)=Suborbital shelf absent.

### Oral Jaws

19. Supramaxilla (modified from Girard et al., 2020 character 24):

Among the taxa in this study, a supramaxilla is present in *Lates calcarifer, Lepomis cyanellus,* and *Nematistius pectoralis*.

(19_0_)=Supramaxilla present.

(19_1_)=Supramaxilla absent.

20. Premaxillary groove:

On the lateral aspect of the premaxilla, a distinct groove is present in some taxa that originates near the anteriormost portion of the premaxilla and runs just above the band of dentition associated with the upper oral jaw. When the oral jaws are closed, the maxilla fit snuggly into this groove. Taxa that lacked a premaxillary groove include *Lepomis cyanellus* and *Perca flavescens*. All other taxa in this study possess a premaxillary groove.

(20_0_)=Lateral aspect of premaxillae smooth and lack groove.

(20_1_)=Lateral aspect of premaxillae with distinct groove dorsal to the band of dentition on the upper oral jaw.

21. Interaction between ascending process and articular process of premaxilla (Girard et al., 2020 character 30):

Among the taxa in this study, the premaxillary process is closely applied or joined to the articular process of premaxilla in *Lepomis cyanellus, Leptobrama muelleri,* and all members of the Toxotidae.

(21_0_)= Ascending process distinct from articular process of premaxilla.

(21_1_)= Ascending process closely applied or joined to articular process of premaxilla.

22. External process on maxilla (Girard et al., 2020 character 33):

Among the taxa in this study, taxa without an external process include *Leptobrama muelleri* and *Perca flavescens*.

(22_0_)=External process on maxilla absent.

(22_1_)=External process on maxilla present.

23. Direction of external process of the maxilla (Girard et al., 2020 character 34):

Among the taxa in this study, taxa with a laterally pointed external process include *Lates calcarifer* and *Lepomis cyanellus*.

(23_0_)=External process on maxilla laterally directed.

(23_1_)=External process on maxilla dorsally directed.

24. Depth of maxilla posteriorly:

In some lineages of the Carangiformes (e.g., Carangidae, Latidae, Polynemidae; Greenwood et al., 1976; Girard et al., 2020), the maxilla becomes dorsoventrally deeper moving from the anterior-to-posterior direction of the element. However, we found the maxilla maintains a largely similar depth throughout the length of the element among species of archerfishes. This consistent depth is only present within the Toxotidae.

(24_0_)=Posterior portion of maxilla deeper than anterior portion of maxilla.

(24_1_)=Maxilla depth largely consistent throughout length of element.

25. Teeth on dentary visible and on external face of bone (Girard et al., 2020 character 52):

Among the taxa in this study, taxa with teeth on dentary visible and on external face of bone include *Lepomis cyanellus, Leptobrama muelleri,* and *Perca flavescens*.

(25_0_)=Teeth restricted to dorsal margin of dentary.

(25_1_)=Teeth on external face of dentary.

26. Ventral protuberance on dentary:

Among the taxa in this study, a ventral protuberance is present on the anteriormost aspect of the dentary. This vertical-shelf-like protuberance, when present, possesses a serrated ventral margin, which became smooth posteriorly as the protuberance joined in with the remainder of the dentary. Members of the Toxotidae are the only taxa in this study to exhibit a ventral protuberance on the dentary.

(26_0_)=Dentary without ventral protuberance.

(26_1_)=Dentary with ventral protuberance on the anteriormost aspect of the element.

27. Endosteal process on coronoid process of articular:

The coronoid process is found on the dorsal margin of the articular element (Rojo, 2018). While the dorsal margin of the coronoid process has been shown to vary in a variety of fishes (e.g., Hexagrammidae [Shinohara, 1994]; Labridae [Westneat, 1993]), including the taxa in this study (see below), a distinct endosteal process is found associated with the coronoid process of the articular. This endosteal process, when present, is found along the posterior aspect of the coronoid process, is pointed dorsally, and typically possesses a cartilaginous (i.e., alcian stained) cap. Most of the taxa in this study possesses an endosteal process on the coronoid process of the articular. Taxa that lacked an endosteal process associated with the coronoid process include *Leptobrama muelleri, Nematistius pectoralis,* and Pro*toxotes lorentzi*.

(27_0_)=Endosteal process associated with coronoid process of articular.

(27_1_)=Coronoid process of articular without endosteal process.

28. Position of endosteal process on coronoid process of articular:

As noted above, many of the taxa in this study possess an endosteal process associated with the coronoid process of the articular. When present, the position of this endosteal process varies with respect to the posterior margin of the coronoid process. The endosteal process is either contained within the margin of the coronoid process of the articular or is posteriorly displaced, extending beyond the posteriormost margin of the articular coronoid process. Many taxa that possess an endosteal process associated with the coronoid process have it posteriorly displaced. Taxa where the endosteal process is contained within the margin of the coronoid process of the articular include *Toxotes chatareus, T. jaculatrix,* and *T. oligolepis*.

(28_0_)=Endosteal process associated with coronoid process of articular posteriorly displaced, extending beyond margin of articular.

(28_1_)=Endosteal process associated with coronoid process of articular contained within the margin of the coronoid process of the articular.

29. Shape of coronoid process of articular:

As noted above, the shape of the coronoid process of the articular has been shown to vary in a variety of fishes. Among the taxa in this study, the coronoid process of the articular varies from a bluntly rounded to distinctly pointed process. Many of the taxa in this study possess a pointed coronoid process, including *Lepomis cyanellus, Nematistius pectoralis, Toxotes blythii, T. carpentariensis, T. chatareus, T. kimberleyensis,* and *T. sundaicus*.

(29_0_)=Coronoid process of articular rounded.

(29_1_)=Coronoid process of articular pointed.

### Hyopalatine Arch

30. Medial margin of the palatine:

Among outgroup taxa, the palatine is typically an elongate element, with a medial margin consisting of flattened bone. However, among the members of the Toxotidae in this study, the medial aspect of the palatine possesses a cup-like indentation that interacted with the lateral aspect of the vomer. No other taxa in this study possess a medial cup-like fossa on the palatine other than members of the Toxotidae.

(30_0_)=Medial margin of palatine consists of flattened bone without a fossa.

(30_1_)=Medial margin of palatine with distinct cup-like fossa.

31. Endopterygoid teeth:

Among taxa examined in this study, dentition associated with the endopterygoid, when present, is ankylosed to the element. This state is found in *Leptobrama muelleri* and all members of the Toxotidae.

(31_0_)=Endopterygoid teeth absent.

(31_1_)=Endopterygoid teeth present and ankylosed to element.

32. Ectopterygoid teeth:

Dentition is present on the ectopterygoid in *Lates calcarifer, Leptobrama muelleri,* and all members of the Toxotidae.

(32_0_)=Ectopterygoid teeth absent.

(32_1_)=Ectopterygoid teeth present.

33. Contact between ectopterygoid and metapterygoid (modified from Girard et al., 2020 character 48):

In most carangiform fishes, the ectopterygoid and metapterygoid can be distinctly separate or abut (Girard et al., 2020). However, in some taxa, a single pointed process originating from the posterior margin of the ectopterygoid extended posteriorly and inserts into an invagination on the metapterygoid. This pointed ectopterygoid process is present in *Lates calcarifer, Leptobrama muelleri,* and *Nematistius pectoralis*.

(33_0_)=Ectopterygoid and metapterygoid distinct and separate elements.

(33_1_)=Ectopterygoid with single pointed process that extends posteriorly and becomes enveloped by invagination on metapterygoid.

34. Posterior margin of metapterygoid:

In a subset of taxa in this study, the posterior margin of the metapterygoid possesses a flange-like extension of bone that overlapped the ventral arm of the hyomandibular, often continuing to overlap elements of the opercular series as well. When present, this flange-like extension on the metapterygoid typically tapered to a distal point. Among the taxa in this study, a posterior flange-like extension is present on the metapterygoid and overlaps the ventral arm of the hyomandibular in *Leptobrama muelleri* and all members of the Toxotidae.

(34_0_)=Posterior margin of metapterygoid simple.

(34_1_)=Posterior margin of metapterygoid with flange-like extension that overlaps hyomandibular ventral arm.

35. Dorsal margin of symplectic (modified from Girard et al., 2020 character 44):

The symplectic is often a tubular bone that varies in shape and overall length. In many of the taxa in this study, the symplectic possesses a lamellar extension of bone that extended from the dorsal margin of the element. When present, this lamellar extension often overlaps the ventroposterior aspect of the metapterygoid. Taxa with a lamellar extension from a dorsal margin of the symplectic include all members of the Toxotidae except *Toxotes kimberleyensis*. This character remains unknown (?) for *Toxotes kimberleyensis* as it is unable to be observed in our material examined.

(35_0_)=Symplectic simply shaped and largely tubular.

(35_1_)=Symplectic with lamellar bone extending from the dorsal margin.

36. Ventral margin of symplectic:

As noted above, the dorsal margin of the symplectic differentially possesses a lamellar extension of bone. In addition to the dorsal lamellar extension, the ventral margin of the symplectic possesses a ventrally-directed lamellar extension in some taxa. Taxa with a ventral extension of lamellar bone from the symplectic include *Lates calcarifer* and all members of the Toxotidae.

(36_0_)=Symplectic simply shaped and largely tubular.

(36_1_)=Symplectic with lamellar bone extending from the ventral margin.

### Opercular Series

37. Serrations on vertical arm of preopercle (modified from Girard et al., 2020 character 19):

Among the taxa in this study, serrations are present on the vertical arm of the preopercle in *Lates calcarifer* and *Perca flavescens*.

(37_0_)=Serrations absent on vertical arm of preopercle.

(37_1_)=Serrations present on vertical arm of preopercle.

### Hyoid Arch

38. Number of branchiostegal rays:

McAllister's (1968) study of the branchiostegal rays across the diversity of fishes shows that the number and attachment of branchiostegal rays vary widely––with increases or decreases in the number of elements as well as differential attachment of these elements to the ceratohyal and epihyal. Among the taxa in this study, six branchiostegal rays are present in *Lepomis cyanellus, Leptobrama muelleri,* and *Protoxotes lorentzi*.

(38_0_)=Seven branchiostegal rays present.

(38_1_)=Six branchiostegal rays present.

39. Beryciform foramen (Girard et al., 2020 character 57):

Taxa with a beryciform foramen include *Lates calcarifer, Lepomis cyanellus,* and *Nematistius pectoralis*.

(39_0_)=Beryciform foramen absent.

(39_1_)=Beryciform foramen present.

40. Posterior elevation of basihyal (Girard et al., 2020 character 64):

Among taxa in this study, only members of the Toxotidae possess a basihyal that is plate-like anteriorly and possess an elevation posteriorly.

(40_0_)=Basihyal largely uniform and flat posteriorly.

(40_1_)=Basihyal plate-like anteriorly with posterior elevation.

41. Shape of basihyal when viewed dorsally:

When viewing the basihyal from the dorsal aspect, the overall shape of the element varies among the taxa in this study. The basihyal ranged from a largely rectangular shape to an ovoid shape. Taxa with an ovoid basihyal when viewed dorsally include *Leptobrama muelleri* and all members of the Toxotidae.

(41_0_)=Basihyal largely rectangular in shape when viewed dorsally.

(41_1_)=Basihyal ovoid in shape when viewed dorsally.

42. Widest portion of ovoid basihyal:

As noted above, the overall shape of the basihyal varies among the taxa in this study. Among taxa with an ovoid basihyal, variation is also present in the position of the element’s greatest width, which ranges from an anterior to a medioposterior position. Taxa with an ovoid basihyal with the greatest width medioposteriorly include *Leptobrama muelleri, Toxotes jaculatrix,* and *T. oligolepis*. Taxa with a largely rectangular basihyal (character 41) are coded as inapplicable (-) for this character.

(42_0_)=Ovoid basihyal obtains greatest width medioposteriorly.

(42_1_)=Ovoid basihyal obtains greatest width anteriorly.

43. Basihyal dentition (Girard et al., 2020 character 65):

Of the taxa in this study, dentition associated with the basihyal is found in *Leptobrama muelleri* and all members of the Toxotidae.

(43_0_)=No dentition on basihyal.

(43_1_)=Dentition present on basihyal.

44. Position of rostral cartilage of basihyal:

As noted above, the overall shape of the basihyal varies among the taxa in this study. Variation is also present in the placement the rostral cartilage, or cap, of the element. Among the taxa in this study, the rostral cap of the basihyal is either in line with the dorsal plane of the ossified basihyal or is ventrally displaced. Taxa with the rostral cartilaginous cap of the basihyal being ventrally displaced include *Leptobrama muelleri* and *Protoxotes lorentzi*.

(44_0_)=Rostral cartilaginous cap of basihyal in the same or similar dorsal margin to ossified basihyal.

(44_1_)= Rostral cartilaginous cap of basihyal ventrally displaced.

45. Rostral expansion of basihyal teeth:

As noted above, teeth are differentially present or absent on the basihyal among the taxa in this study. For taxa with basihyal teeth, the anterior limit of basihyal dentition varies. Teeth on the basihyal are either restricted and absent from the rostral margin of the basihyal or extend to or close to the rostral margin of the element. Taxa with basihyal dentition that is restricted and absent from rostral margin of the basihyal include *Toxotes blythii* and *T. carpentariensis*. This character remains unknown (?) for *Toxotes kimberleyensis* and *T. sundaicus* as the state for these taxa are not able to be observed in our material examined. Taxa that lacked dentition associated with the basihyal (character 44) are coded as inapplicable (-) for this character.

(45_0_)=Basihyal dentition restricted and absent from rostral margin of the basihyal.

(45_1_)=Basihyal dentition extends to the rostral margin of the basihyal.

46. Ventral hypohyal with enlarged ventral spur:

As shown by McAllister (1968), the ceratohyal may interact with the ventral hypohyal via a bony protuberance that is enveloped by a fossa on the caudal aspect of the ventral hypohyal (e.g., Bothidae, Eleotridae, Monocentridae, Ophidiidae, Pleuronectidae). Underneath this fossa, on the ventral aspect of the ventral hypohyal, a subset of these fishes exhibit a spur-like process that extends ventrally and caudally and varies in overall size (Batrachoididae, Bothidae, Carangidae, Cobitidae, Mullidae, Pleuronectidae, Priacanthidae; McAllister, 1968). Among the taxa in this study, this ventral spur or process is greatly enlarged in some taxa, appearing almost leaf-like. An enlarged ventral spur is present on the ventral hypohyal in *Leptobrama muelleri* and members of the Toxotidae.

(46_0_)=Ventral hypohyal without enlarged ventral spur.

(46_1_)=Enlarged ventral spur extending ventrally and caudally on ventral hypohyal.

47. Shape of posterior margin of ventral hypohyal spur:

As noted above, an enlarged spur is differentially present or absent on the ventral margin of the ventral hypohyal in some taxa. When present, the shape of the posterior margin of this spur varies among the taxa examined. The posterior margin of the ventral hypohyal spur varies from a broad, leaf-like shape, to tapering to a distinct point. A broad and leaf-like posterior margin on the ventral hypohyal spur is present in *Protoxotes lorentzi* and *Toxotes jaculatrix*.

(47_0_)=Posterior margin of ventral hypohyal spur broad and leaf-like.

(47_1_)=Posterior margin of ventral hypohyal spur tapering.

48. Ventral margin of ceratohyal:

In half of the outgroup taxa, the ventral margin of the ceratohyal is smooth and interacts with the anteriormost branchiostegal rays. However, in some of the taxa in this study, the ventral margin of the ceratohyal possesses a pronounced ventrally-directed spur that often interacts with the third branchiostegal ray. This ventral spur on the ceratohyal is present in *Leptobrama muelleri, Perca flavescens,* and all species of *Toxotes*.

(48_0_)=Ventral margin of ceratohyal smooth.

(48_1_)=Ventral margin of ceratohyal with distinct ventrally-directed spur.

49. Basihyal keel:

In addition to the variation in shape and dentition associated with the basihyal, the ventral aspect of the basihyal also contained variation among the taxa in this study. In the leptobramid and toxotids, the ventral aspect of the basihyal possesses a distinct ventrally-directed keel that traveled along the mesial aspect of the element. Only *Leptobrama muelleri* and all species of the Toxotidae possess a keel on the ventral aspect of the basihyal.

(49_0_)=Basihyal keel absent.

(49_1_)=Basihyal keel present.

50. Basihyal position (Girard et al., 2020 character 63):

The basihyal inserts above basibranchial one and covers the element when viewing the branchial and hyoid arches dorsally in *Leptobrama muelleri* and all species in the Toxotidae.

(50_0_)=Basihyal inserts anterior to basibranchial one and does not cover the entirety of the first basibranchial when viewing the branchial and hyoid arches dorsally.

(50_1_)=Basihyal inserts above basibranchial one and covers the first basibranchial when viewing the branchial and hyoid arches dorsally.

### Gill Basket

51. Posterior margin of basibranchial one:

Most of the outgroup taxa lack an interaction between the posterior margin of basibranchial one and the anterior margin of basibranchial two. However, in toxotid taxa, the posterior margin of basibranchial one possesses a caudally directed protuberance that overlapped the anterior margin of basibranchial two. This morphology is present in *Leptobrama muelleri* and all members of the Toxotidae. This character remains unknown (?) for *Nematistius pectoralis* as it is unable to be observed in our material examined.

(51_0_)=Posterior margin of basibranchial one confluent and does not overlap anterior margin of basibranchial two.

(51_1_)=Posterior margin of basibranchial one with caudally directed protuberance that overlaps anterior margin of basibranchial two.

52. Basibranchial three dentition (Girard et al., 2020 character 72):

Most of the taxa in this study lack dentition associated with the third basibranchial, while *Lates calcarifer* and *Lepomis cyanellus* possess dentition on basibranchial three.

(52_0_)=No dentition present on basibranchial three.

(52_1_)=Toothplate(s) present on basibranchial three.

53. Basibranchial three shape:

Among all non-toxotid taxa, the third basibranchial is a largely rectangular or hourglass shaped element that is elongate. However, members of the Toxotidae in this study possess lateral expansions of the third basibranchial, thereby making the overall appearance reminiscent of the letter T. The lateral expansions on the third basibranchial typically covered the third hypobranchial processes dorsally. No other taxa outside of the Toxotidae possess lateral expansions on the third basibranchial.

(53_0_)=Basibranchial three largely rectangular or hourglass shaped.

(53_1_)=Basibranchial three with lateral expansions, T-shaped.

54. Hypobranchial three process:

The third hypobranchial possesses a ventral process which typically lays laterally to the urohyal and can vary in overall length. Among the taxa in this study, the third hypobranchial process varies from a long process to a short process. Taxa with a short hypobranchial three process include *Lepomis cyanellus* and all species in the Toxotidae.

(54_0_)=Hypobranchial three process long.

(54_1_)=Hypobranchial three process short.

55. Shape of lateral gill rakers on hypobranchial one (based on Girard et al., 2020 character 79):

All taxa in this study possess lateral gill rakers associated with hypobranchial one. However, the shape of the gill rakers associated with hypobranchial one varies among the taxa in this study. The overall shape ranges from anterior lateral gill rakers being flattened and posterior gill rakers being tubercle shaped, to flattened and broad plates covering the first hypobranchial. Taxa with all hypobranchial one gill rakers being flattened into plates include *Leptobrama muelleri, Nematistius pectoralis,* and all species in the Toxotidae*.*

(55_0_)=Lateral gill rakers on hypobranchial a mix of flattened and tubercle shaped.

(55_1_)=All lateral gill rakers on hypobranchial one as flattened plates.

56. Shape of lateral gill rakers on branchial arches two through four (modified from Girard et al., 2020 character 81):

Girard et al. (2020) note variation in the overall shape of the lateral gill rakers among carangiform and outgroup taxa. In this study, all taxa possess lateral gill rakers on branchial arches two through four, and we denote two gill-raker shapes that follow Girard et al. (2020): 1) flattened gill rakers that are plate-like, with little to no expansion of the gill raker; and 2) raised gill rakers, which are similar to that of the 'tubercle-shape gill raker' shown in the study by Märss et al. (2017). Among the taxa in this study, taxa with raised gill rakers on the lateral aspect of branchial arches two through four are only found in *Lepomis cyanellus* and *Perca flavescens*.

(56_0_)=Largely flat gill rakers on the lateral aspect of branchial arches two through four.

(56_1_)=Raised gill rakers on the lateral aspect of branchial arches two through four.

57. Dorsal broadening of lateral gill rakers on branchial arches two through four (Girard et al., 2020 character 81):

Taxa with lateral gill rakers with dorsal broadening around margin include *Leptobrama muelleri, Toxotes blythii, T. carpentariensis, T. chatareus*.

(57_0_)=Lateral gill rakers largely uniform throughout margin.

(57_1_)=Lateral gill rakers with dorsal broadening around margin.

58. Proximity of fifth left and right ceratobranchial toothplates medially:

In many taxa, the left and right fifth ceratobranchial toothplates are closely applied medially along approximately half of the length of the elements. However, the left and right ceratobranchial toothplates are distinctly separate in some taxa. Taxa with the left and right ceratobranchial toothplates separate include *Lates calcarifer, Lepomis cyanellus, Nematistius pectoralis, Toxotes blythii, T. carpentariensis, T. chatareus, T. oligolepis,* and *T. sundaicus*.

(58_0_)=Left and right fifth ceratobranchial toothplates closely applied medially.

(58_1_)=Left and right fifth ceratobranchial toothplates separated medially.

59. Size of gap between spaced apart fifth ceratobranchial toothplates:

As noted above, some taxa examined in this study possess a gap between the left and right fifth ceratobranchial toothplates. For taxa exhibiting this separated condition, the size of the gap varies from a medium gap to a large gap. Taxa with a large gap include *Nematistius pectoralis* and *Toxotes sundaicus*. Taxa with closely applied left and right fifth ceratobranchial toothplates (character 58) are coded as inapplicable (-) for this character.

(59_0_)=Medium sized gap between left and right fifth ceratobranchial toothplates.

(59_1_)=Large sized gap between left and right fifth ceratobranchial toothplates.

60. Length of dorsal and ventral processes of lateral gill raker associated with the junction between the first epibranchial and first ceratobranchial:

As noted above, most taxa in this study possess a lateral gill raker that is associated with the junction between the first epibranchial and first ceratobranchial. When present, a dorsal and ventral process emerge from the proximal aspect of the gill raker and interact with the epibranchial or ceratobranchial, respectively. The overall length of these processes vary from being equal to the dorsal process being reduced in length when compared to the ventral process. Taxa with the dorsal process being reduced in length when compared to the ventral processes on the lateral gill raker that associated with the junction between the first epibranchial and first ceratobranchial include *Toxotes blythii, T. jaculatrix, T. kimberleyensis,* and *T. oligolepis*. This character is coded as inapplicable (-) for *Protoxotes lorentzi* as this taxon lacked a gill raker on the junction between the first epibranchial and first ceratobranchial.

(60_0_)=Dorsal process of lateral gill raker equal to that of ventral process.

(60_1_)=Dorsal process of lateral gill raker reduced in length compared to that of the ventral process.

61. Shape of lateral gill rakers on first epibranchial:

Lateral gill rakers are found on the first epibranchial in all taxa examined in this study. However, the shape of these lateral gill rakers varies. Taxa possess either all flattened lateral gill rakers or lateral gill rakers that are tubercle-like or elongate in shape. Most of the taxa in this study possess tubercle-like to elongate gill rakers. Taxa with lateral gill rakers that are all flattened in shape include *Toxotes jaculatrix* and *Protoxotes lorentzi*.

(61_0_)=Lateral gill rakers on epibranchial one tubercle-like or elongate in shape.

(61_1_)=Lateral gill rakers on epibranchial one flattened in shape.

62. Proximity of dorsalmost epibranchial two toothplate with epibranchial:

As noted above, a toothplate associated with the second epibranchial is differentially present or absent among the taxa in this study. For taxa that possess a second epibranchial toothplate, the proximity between the dorsal most toothplate and the second epibranchial varies from being closely applied to spaced apart. Most of the taxa in this study possess the spaced apart condition. Taxa with the dorsalmost epibranchial two toothplate closely applied to the second epibranchial include *Lates calcarifer* and *Nematistius pectoralis*. Taxa that do not exhibit one or more epibranchial two toothplate(s) are coded as inapplicable (-) for this character.

(62_0_)=Dorsalmost epibranchial two toothplate closely applied to epibranchial two.

(62_1_)=Dorsalmost epibranchial two toothplate spaced apart from epibranchial two.

### Pectoral Girdle

63. Position of sensory canal passing through the supracleithrum:

While the trunk sensory canal passes through a pore in the supracleithrum in all taxa in this study, the position of this sensory canal pore on the supracleithrum varies. In some taxa, the sensory canal is in a more ventral position, passing through the supracleithrum but not near the dorsal margin of the element. Taxa with a more ventrally positioned sensory canal in the supracleithrum include *Lates calcarifer, Leptobrama muelleri, Nematistius pectoralis,* and *Protoxotes lorentzi*.

(63_0_)=Sensory canal positioned near or in the dorsal margin of supracleithrum.

(63_1_)=Sensory canal positioned within the supracleithrum but not near dorsal margin.

64. Medial extrascapular (Girard et al., 2020 character 103):

Taxa that possess a medial extrascapular include *Lepomis cyanellus, Nematistius pectoralis,* and *Perca flavescens*.

(64_0_)=Medial extrascapular absent.

(64_1_)=Medial extrascapular present.

65. Length of coracoid relative to cleithrum (Girard et al., 2020 character 113):

Taxa with a ventrally-elongate coracoid that reaches an equal or more ventral plane than the cleithrum include *Leptobrama muelleri* and all species in the Toxotidae.

(65_0_)=Ventral process of the coracoid does not reach same ventral plane as cleithrum.

(65_1_)=Ventral process of the coracoid reaches similar or past the ventral plane of cleithrum.

66. Proximity of ventral aspects of coracoid and cleithrum (Girard et al., 2020 character 114):

Taxa that exhibit separation between the cleithrum and ventral process of the coracoid include *Leptobrama muelleri* and all species in the Toxotidae.

(66_0_)=Coracoid and ventral process of the cleithrum in close proximity or touching at ventral aspect.

(66_1_)=Coracoid and ventral process of the cleithrum are not close in proximity; distinctly separate.

67. Width of the ventral process of the coracoid (Girard et al., 2020 character 115):

A robust coracoid that is widened by lamellae can be found in all species in the Toxotidae.

(67_0_)=Ventral process of coracoid tapering to rod-like process.

(67_1_)=Ventral process of coracoid broadened by lamellae; not rod-like at ventral aspect.

68. Shape of broadened ventral process of coracoid:

As noted above, the ventral process of the coracoid can vary from a rod-like process, to being widened by lamellae. Among taxa with a broad ventral process of the coracoid, the overall shape of this expanded element varies from a tapering process to a broad and largely rhomboid process. Taxa with a tapering ventral process of the coracoid include *Toxotes blythii*, *T. sundaicus,* and *Protoxotes lorentzi*. Taxa that possess a ventral process of the coracoid that is rod-like (character 67) are coded as inapplicable (-) for this character.

(68_0_)=Broadened ventral process of the coracoid tapering in shape.

(68_1_)=Broadened ventral process of the coracoid wide and largely rhomboid in shape.

69. Strength of postcoracoid process:

When present, the postcoracoid process is on the dorsalmost and posteriormost angle of the coracoid, directly underneath the lowermost pectoral radial. While all the taxa in this study possess a postcoracoid process, the strength of this process varies. The process ranged from a weak process and a shallow dorsal indentation to a strong process with a deep dorsal indentation. Taxa with a strong postcoracoid process include *Lates calcarifer, Lepomis cyanellus, Leptobrama muelleri, Perca flavescens,* and *Protoxotes lorentzi*.

(69_0_)=Postcoracoid process weak with shallow indentation dorsally.

(69_1_)=Postcoracoid process strong with deep indentation dorsally.

70. Medioposterior margin of the cleithrum:

While many taxa possess a smooth medioposterior margin of the cleithrum, a subset of taxa in this study possess a caudally directed protuberance present on the medioposterior margin of the cleithrum. When present, this protuberance is short and seemingly articulates with the anterior processes of the basipterygium. Taxa with a protuberance on the medioposterior margin of the cleithrum include *Leptobrama muelleri* and all species in the Toxotidae.

(70_0_)=Medioposterior margin of cleithrum smooth.

(70_1_)=Medioposterior margin of cleithrum with protuberance.

71. Margins of scapula and coracoid:

The scapula and coracoid are typically distinct elements with abutting margins in most of the taxa in this study. However, two taxa, *Leptobrama muelleri* and *Nematistius pectoralis*, possess suturing between the scapula and coracoid.

(71_0_)=Margins of scapula and coracoid distinct and abutting.

(71_1_)=Margins of scapula and coracoid sutured.

72. Margins of cleithrum and coracoid dorsally:

As noted above, suturing occurs between the scapula and coracoid in some taxa in this study. In addition to this suturing, suturing also occurred between the margins of the cleithrum and coracoid dorsally. Taxa that possess suturing between the cleithrum and coracoid dorsally include *Leptobrama muelleri* and *Nematistius pectoralis*.

(72_0_)=Margins of cleithrum and coracoid dorsally distinct and abutting.

(72_1_)=Margins of cleithrum and coracoid dorsally sutured.

### Pelvic Girdle

73. Ultimate pelvic-fin ray relationship to abdomen (based on Allen, 2004):

In his description of *Toxotes kimberleyensis*, Allen (2004) notes that the innermost pelvic-fin ray is attached to the abdomen by a membrane. In expanding this character and applying it to the taxa in this study, we find the ultimate pelvic-fin ray connected to abdomen in *Leptobrama muelleri* and all species of the Toxotidae.

(73_0_)=Ultimate pelvic-fin ray free from abdomen.

(73_1_)=Ultimate pelvic-fin ray connected to abdomen via membrane.

### Dorsal Fin

74. Insertion of first dorsal-fin pterygiophore:

The insertion of the first dorsal-fin pterygiophore has been used by many authors (e.g., Munroe, 1992; Morgan and Gill, 2006; Springer and Smith-Vaniz, 2008) as a phylogenetically informative character. Given the variation in dorsal-fin morphology across the taxa in this study (see subsequent characters), we also assess the variation in the insertion of the first dorsal-fin pterygiophore. We treat the space between the posterior elements of the neurocranium and first neural spine as interneural space one. Taxa examined in this study possess a first dorsal-fin pterygiophore in the third or anterior to the third interneural space, in the fourth interneural space, and in the fifth interneural space. Taxa with the first dorsal-fin pterygiophore inserting in the fourth interneural space include only *Leptobrama muelleri*. All members of the Toxotidae possesses the first dorsal pterygiophore inserting in the fifth interneural space.

(74_0_)=First dorsal pterygiophore inserting in third or anterior to third interneural space.

(74_1_)=First dorsal pterygiophore inserting in fourth interneural space.

(74_2_)=First dorsal pterygiophore inserting in fifth interneural space

75. Anteriormost dorsal-fin spine association with proximal-middle pterygiophore (modified from Johnson, 1986 characters 2 and 48 and Girard et al., 2020 character 136):

Among taxa in this study, those with the anteriormost dorsal-fin spine associated in serial association with the proximal-middle pterygiophore include *Lates calcarifer, Lepomis cyanellus, Leptobrama muelleri, Perca flavescens,* and all species in the Toxotidae.

(75_0_)=Single spine in serial correspondence associated with dorsal pterygiophore.

(75_1_)=Two or more supernumerary spines associated with dorsal pterygiophore.

76. Spineless proximal-middle pterygiophores of spinous dorsal fin (modified from Girard et al., 2020 character 135):

Spine(s) can be lost from the anteriormost proximal-middle pterygiophore of the spinous dorsal fin. In a subset of taxa, one or more spineless proximal-middle pterygiophores may be present in the spinous dorsal (e.g., Leptobramidae, Toxotidae; Girard et al., 2020). Among the taxa in this study, one or more spineless proximal-middle dorsal pterygiophores are found in *Leptobrama muelleri*, *Toxotes jaculatrix,* and *T. oligolepis* exclusively.

(76_0_)=Spineless proximal-middle dorsal-fin pterygiophore absent.

(76_1_)=One or more spineless proximal-middle dorsal-fin pterygiophores present.

77. Insertion of external morphology of dorsal fin relative to posterior margin of pectoral fin (based on Kottelat and Tan, 2018):

In their description of three new species of archerfishes, Kottelat and Tan (2018) note variation in the relationship between the posterior margin of the pectoral fin and the anterior insertion of the dorsal fin, with the pectoral fin reaching or not reaching the vertical through the dorsal-fin origin. In expanding this character and applying it to the taxa in this study, taxa with a dorsal fin that inserts posteriorly to posterior margin of pectoral fin include *Leptobrama muelleri* and *Protoxotes lorentzi*.

(77_0_)=External morphology of dorsal fin inserts anteriorly or above posterior margin of pectoral fin.

(77_1_)=External morphology of dorsal fin inserts posteriorly to posterior margin of pectoral fin.

78. Number of dorsal-fin spines:

The number of dorsal-fin spines has been used by Allen (1978, 2004) to differentiate different species of archerfishes. In expanding this character and applying it to the taxa in this study, the number of dorsal-fin spines varies from ten or more to four or fewer. Taxa with between nine and seven dorsal-fin spines present include *Lates calcarifer* and *Nematistius pectoralis*. Taxa with between five and six dorsal-fin spines present include *Toxotes blythii, T.* *carpentariensis, T. chatareus, T. kimberleyensis,* *T. sundaicus,* and *Protoxotes lorentzi*. Taxa with four or fewer dorsal-fin spines present include *Leptobrama muelleri* and *Toxotes jaculatrix*.

(78_0_)=Ten or more dorsal-fin spines present.

(78_1_)=Between seven and nine dorsal-fin spines present.

(78_2_)=Between five and six dorsal-fin spines present.

(78_3_)=Four or fewer dorsal-fin spines present.

79. Tripartite pterygiophores in posteriormost elements of soft dorsal fin (Girard et al., 2020 character 142):

Among taxa in this study, bipartite pterygiophores are exclusively present in the posteriormost pterygiophores of the soft-dorsal fin in *Lates calcarifer, Lepomis cyanellus,* and *Nematistius pectoralis*.

(79_0_)=Posteriormost pterygiophores of soft-dorsal fin bipartite.

(79_1_)=One or more tripartite pterygiophores in posteriormost elements of soft dorsal fin.

### Anal Fin

80. Dorsal- and anal-fin pterygiophore stays (Girard et al., 2020 character 145):

Among taxa in this study, many of the taxa in this study lack a dorsal- and anal-fin pterygiophore stay, with *Lates calcarifer, Lepomis cyanellus, Leptobrama muelleri, Nematistius pectoralis,* and *Perca flavescens* exhibiting both pterygiophore stays.

(80_0_)=Dorsal- and anal-fin pterygiophore stays present.

(80_1_)=Dorsal- and anal-fin pterygiophore stays absent.

81. Posterior elongation of dorsal- and anal-fin pterygiophore stays (Girard et al., 2020 character 146):

Taxa with elongate stays that extend posteriorly include *Leptobrama muelleri* and *Nematistius pectoralis*. This character is coded as inapplicable (-) for taxa that do not possess a dorsal- and anal-fin pterygiophore (character 80).

(81_0_)=Dorsal- and anal-fin pterygiophores smaller, square-like elements.

(81_1_)=Dorsal- and anal-fin pterygiophores elongate, extending posteriorly toward the caudal fin.

82. Number of anal-fin pterygiophores anterior to first hemal spine (modified from Girard et al., 2020 character 147):

While most of the taxa in this study possess only one anal-fin pterygiophore anterior to the first hemal spine, taxa with two anal-fin pterygiophores anterior to the first hemal spine include *Lepomis cyanellus* and *Toxotes oligolepis*.

(82_0_)=One anal-fin pterygiophore anterior to first hemal spine.

(82_1_)=Two anal-fin pterygiophores anterior to first hemal spine.

83. Angle of first anal-fin pterygiophore (modified from Girard et al., 2020 character 153):

Across the taxa in this study, the angle of the first anal-fin pterygiophore varies from being inclined at an oblique angle, to a largely vertical element. Taxa with a first anal-fin pterygiophore in a largely vertical position include *Leptobrama muelleri* and all species in the Toxotidae.

(83_0_)=First anal-fin pterygiophore inclined at oblique angle.

(83_1_)=First anal-fin pterygiophore positioned largely vertically.

84. Shape of first anal-fin pterygiophore (modified from Girard et al., 2020 character 153):

As noted above, the angle of the first anal-fin pterygiophore varies among the taxa in this study. In addition to the variation in the angle of the element, the overall shape of the element also varies from a largely strut-like and linear first anal-fin pterygiophore to an anteriorly recurved anal pterygiophore. Taxa with an anteriorly recurved anal-fin pterygiophore include all species of *Toxotes*. The character remains unknown (?) for *Protoxotes lorentzi* as the character state could not be determined for this taxon.

(84_0_)=First anal-fin pterygiophore largely strut-like and linear.

(84_1_)=First anal-fin pterygiophore recurved anteriorly.

**Postcranial Skeleton**

85. Overall body physiognomy:

Many authors that have differentiated and described new species of archerfishes (e.g., Allen, 2004; Kottelat and Tan, 2018) have commented on the overall shape or physiognomy of the body. Among the taxa in this study, overall body physiognomy ranges from an elongate or largely elongate body to a more robust and rounded body. Taxa exhibiting a robust and rounded body shape include *Lepomis cyanellus, Toxotes blythii, T.* *carpentariensis, T. chatareus, T. kimberleyensis,* and *T. sundaicus*.

(85_0_)=Body elongate or largely elongate.

(85_1_)=Body more robust and rounded.

86. Connection between ultimate pleural rib and ultimate abdominal vertebra:

Among the taxa in this study, the ultimate pleural rib may not articulate with the ultimate abdominal vertebra. Taxa that do not possess a connection between the ultimate pleural rib and the ultimate abdominal vertebra include all species of *Toxotes*.

(86_0_)=Ultimate pleural rib attached to ultimate abdominal vertebra.

(86_1_)=Ultimate pleural rib not connected to ultimate abdominal vertebra.

87. Shape of pleural rib on last abdominal vertebra (Girard et al., 2020 character 161):

The pleural rib on the ultimate abdominal vertebra is recurved with the distal tip of the ribs directed ventrally in *Leptobrama muelleri, Toxotes* *carpentariensis, T. chatareus, T. jaculatrix, T. kimberleyensis, T. oligolepis,* and *T. sundaicus* among the taxa in this study.

(87_0_)=Pleural rib on ultimate abdominal vertebra simple and largely linear.

(87_1_)=Pleural rib on ultimate abdominal vertebra recurved.

88. Distal tips of posterior abdominal parapophyses (Girard et al., 2020 character 163):

Among taxa in this study, most possess parapophyses that are separated in all abdominal vertebrae. Taxa with the posterior abdominal parapophyses directed medially and joined with the opposite parapophysis, often forming a single spine-like projection with a bifurcating distal tip include *Leptobrama muelleri* and *Nematistius pectoralis*.

(88_0_)=Distal tips of parapophyses separated in all abdominal vertebrae.

(88_1_)=Distal tips of posterior abdominal parapophyses directed medially and joined with the opposite parapophysis, often forming a single spine-like projection with a bifurcating distal tip.

89. Anal-fin ray count versus dorsal-fin ray count (Girard et al., 2020 character 165):

Taxa that possess equal or more fin rays in the dorsal fin include *Leptobrama muelleri* and all species of the Toxotidae.

(89_0_)=More dorsal-fin rays than anal-fin rays.

(89_1_)=Equal or more anal-fin rays than dorsal-fin rays.

### Caudal Skeleton

90. Uroneural two (Girard et al., 2020 character 182):

The second uroneural is absent in most of the fishes in this study. Fishes exhibiting only uroneural one include all species of the Toxotidae.

(90_0_)=Uroneural two present.

(90_1_)=Uroneural two absent.

91. Procurrent spur on ventral aspect of lower procurrent ray (Girard et al., 2020 character 188):

Among taxa in this study, those that possess a procurrent spur include *Lates calcarifer*, *Leptobrama muelleri,* and all species in the Toxotidae.

(91_0_)=Procurrent spur absent.

(91_1_)=Procurrent spur present.

92. Spur on rostral base of caudal-fin ray preceding procurrent ray:

In addition to the procurrent spur on the ventral aspect of the lower procurrent caudal-fin ray, the subsequent procurrent caudal-fin ray may possess a spur among the taxa in this study. Taxa with a spur on the ventral aspect of the second procurrent caudal-fin ray include *Leptobrama muelleri, Toxotes blythii, T. carpentariensis, T. chatareus, T. jaculatrix,* and *T. oligolepis*. This character remains uncoded (?) for *Toxotes kimberleyensis* and *T. sundaicus* as the state could not be observed in these taxa.

(92_0_)= Spur on rostral base of caudal-fin ray preceding procurrent ray absent.

(92_1_)= Spur on rostral base of caudal-fin ray preceding procurrent ray present.

93. Rostral base of caudal-fin ray preceding procurrent ray shortened (Girard et al., 2020 character 189):

Among taxa in this study, those that share a proximal base of caudal-fin ray preceding procurrent ray shortened include *Lates calcarifer, Leptobrama muelleri, Toxotes oligolepis*, and *Protoxotes lorentzi*.

(93_0_)=Proximal base of caudal-fin ray preceding procurrent ray not shortened.

(93_1_)=Proximal base of caudal-fin ray preceding procurrent ray shortened.

94. Number of lower procurrent caudal-fin rays:

In addition to the variation seen in the proximal bases of the lower procurrent caudal-fin rays, the overall number of procurrent caudal-fin rays also varies among taxa in this study. *Protoxotes lorentzi* is the only taxon in this study with six procurrent caudal-fin rays. Taxa with five procurrent caudal-fin rays include all species of *Toxotes*.

(94_0_)=Seven or more procurrent caudal-fin rays present.

(94_1_)=Six procurrent caudal-fin rays present.

(94_2_)=Five procurrent caudal-fin rays present.

### Squamation

95. Scales on the bases of the soft dorsal and anal fins:

One of the diagnostic characters of the traditional Squamipinnes (*sensu* Mok and Shen, 1983) is the presence of scales covering the base of the soft dorsal and anal fins. We coded this character across the taxa in this study prior to clearing and staining the specimens. We found scales covering the bases of the soft dorsal and anal fins of *Nematistius pectoralis,* but these scales are present on flaps of skin that the medial fins fit into. As these scales are not directly associated with the soft dorsal and anal fins, we elected to code this character as absent for *Nematistius pectoralis.* Scales are found covering the bases of the soft dorsal and anal fins in *Leptobrama muelleri* and all species in the Toxotidae.

(95_0_)=Bases of soft dorsal and anal fins without scales.

(95_1_)=Bases of soft dorsal and anal fins covered in scales.

96. Enlarged pelvic axial 'scale' (Girard et al., 2020 character 197):

Taxa that possess this enlarged pelvic axillary 'scale' include *Lates calcarifer*, *Leptobrama muelleri,* and all species in the Toxotidae.

(96_0_)=Pelvic axial 'scale' not enlarged at the point of insertion of the pelvic fin.

(96_1_)=Enlarged pelvic axial 'scale' enlarged at the point of insertion of the pelvic fin.

97. Length of pelvic axillary ‘scale’:

As noted above, many taxa in this study possess an enlarged pelvic axial 'scale.' Allen (2004) notes variation in the overall length of the pelvic axillary scale in his description of *T. kimberleyensis* as it relates to other species of archerfishes. In expanding this character and applying it to the taxa in this study, we quantify the length of the scale into three categories: a pelvic axillary scale that extends up to ⅓ the length of the pelvic spine, a pelvic axillary scale that extends between ⅓ and ⅔ the length of the pelvic spine, and a pelvic axillary scale that extends more than ⅔ the length of the pelvic spine. *Protoxotes lorentzi* exhibits an axillary scale extending up to ⅓ the length of the pelvic spine. Taxa with a pelvic axillary scale extending more than ⅔ the length of the pelvic spine include *Toxotes blythii*, *T. chatareus*, and *T. jaculatrix*. Taxa that lacked a pelvic axillary ‘scale’ (character 96) are coded as inapplicable (-) for this character.

(97_0_)=Pelvic axillary scale extending up to ⅓ the length of the pelvic spine.

(97_1_)=Pelvic axillary scale extending between ⅓ and ⅔ the length of the pelvic spine.

(97_2_)=Pelvic axillary scale extending more than ⅔ the length of the pelvic spine.

98. Number of lateral-line scales:

Given the wide variability in lateral-line scales among members of the Toxotidae (see Allen 1978) and the potential phylogenetic and diagnostic significance of lateral-line scale counts, we assess the variation in the number of lateral line scales in this character. We coded members of the Toxotidae based on the mode number of lateral-line scales gathered from the whole specimens examined in this study. We coded *Toxotes carpentariensis* and *T. chatareus* as both state 0 and state 1, as these species have been shown to exhibit widely different counts of lateral-line scales by previous authors (Allen, 1978, 2004; Kottelat and Tan, 2018) and the specimens examined in this study. This character is coded for *Lates calcarifer* from Pethiyagoda and Gill (2013); *Lepomis cyanellus* from Ross (2001); *Leptobrama muelleri* from Kimura et al., 2016; *Nematistius pectoralis* from Miller and Lea (1972).

(98_0_)=Between 25 and 32 lateral-line scales.

(98_1_)=Between 33 and 50 lateral-line scales.

(98_2_)=Between 51 or more lateral-line scales.

99. Shape of lateral-line canal dorsal to pectoral fin:

Among the taxa in this study, the shape of the lateral line varies above the pectoral fin. Taxa possess either a straight lateral line canal above the pectoral fin, or an arched lateral line canal above the pectoral fin. Those with an arched lateral-line canal dorsal to the pectoral fin include *Lepomis cyanellus, Leptobrama muelleri, Perca flavescens,* and all species of *Toxotes*.

(99_0_)=Lateral-line canal straight above pectoral fin.

(99_1_)=Lateral-line canal arched above pectoral fin.

100. Lateral-line confluence:

Among the members of the Toxotidae in this study, all species except *Protoxotes lorentzi* possess a broken or interrupted lateral line posterior to the pectoral fin. This break in the lateral line is only present in species of *Toxotes*.

(100_0_)=Lateral-line canal confluent.

(100_1_)=Lateral-line canal interrupted.
