# Supplementary material for: Phylogenetics of Archerfishes (Toxotidae) and Evolution of the Toxotid Shooting Apparatus
Source: Integr Org Biol. 2022 Mar 21;4(1):obac013. doi: 10.1093/iob/obac013 (PMC9259087; doi:10.1093/iob/obac013)
Supplement: obac013_Supplemental_Files [file obac013_supplemental_files.zip › Supplementary_table_1_rev.pdf]

	Voucher for Molecular Dataset	Tissue for Molecular Dataset	SRA Accession Numbers	UCE Loci Collected	Total bps	Mean Contig Length (bps)	Median Contig Length (bps)	Minimum Contig Length (bps)	Maximum Contig Length (bps)	Contigs Over 1,000 bps	95% Confidence Interval
**<u>Outgroup Taxa</u>**											
**Centrarchidae**											
Lepomis cyanellus[4]	JFBM Uncat.	See voucher	SRR11016327	457	668,734	1,463.31	1,432	606	3,083	430	15.18
**Percidae**											
Perca flavescens[4]	JFBM Uncat.	See voucher	SRR11016340	459	729,626	1,589.60	1,604	318	3,265	430	16.39
**<u>Carangiformes</u>**											
**Latidae**											
Lates calcarifer[4]	AMNH 233713	See voucher	SRR11016328	457	668,734	1,463.31	1,432	606	3,083	430	15.18
**Leptobramidae**											
Leptobrama muelleri[4]	H. Larson Pers. Col.	See voucher	SRR11016326	449	633,923	1,411.86	1,429	369	3,160	420	12.86
**Nematistiidae**											
Nematistius pectoralis[4]	K. Carpenter Pers. Col.	See voucher	SRR11016342	459	574,659	1,251.98	1,249	553	2,565	417	10.38
**Toxotidae**											
Protoxotes lorentzi[4]	MAGNT S.17681-003	MAGNT A01103	SRR17680287	133	159,149	1,196.61	1,217	161	2,336	98	35.01
Toxotes blythii[4]	KUI 42173	KUIT 11258	SRR11016333	455	852,318	1,873.23	1,858	737	3,853	449	17.54
Toxotes blythii	KUI 42698	KUIT 11263	SRR17680286	427	521,284	1,220.81	1,184	154	2,747	388	12.43
Toxotes blythii	ZRC 60598	See voucher	SRR17680280	324	417,400	1,288.27	1,274	146	3,159	277	20.69
Toxotes carpentariensis[1]	CDU SP-113	See voucher	SRR17680278	370	519,392	1,403.76	1,376	247	2,841	335	19.45
Toxotes carpentariensis[1]	CDU SP-81	See voucher	SRR17680279	420	428,974	1,021.37	982	534	1,984	196	10.51
Toxotes carpentariensis[1]	CDU SP-141	See voucher	SRR17680277	419	511,526	1,220.83	1,156	629	3,388	373	13.56
Toxotes carpentariensis[14]	LSUMZ 17586	LSUMZ F6590	SRR17680276	460	637,110	1,385.02	1,379	446	3,080	433	12.42
Toxotes carpentariensis[1]	LSUMZ 17587	LSUMZ F6596	SRR17680275	462	717,357	1,552.72	1,570	271	3,063	447	12.22
Toxotes chatareus	UF 191640	UF ICH-01933	SRR17680285	268	366,265	1,366.66	1,338	295	2,839	242	20.17
Toxotes chatareus	ZRC Uncat.	See voucher	SRR17680282	392	571,289	1,457.37	1,404	244	3,168	365	19.02
Toxotes chatareus[2]	UF 188548	UF ICH-00930	SRR17680273	410	615,565	1,501.38	1,437	269	3,609	398	16.24
Toxotes chatareus[3]	UF 188329	UF ICH-00115	SRR17680274	418	538,623	1,288.57	1,219	171	2,929	388	15.18
Toxotes chatareus[3]	ZRC 60599	See voucher	SRR17680283	429	548,488	1,278.53	1,260	241	2,811	386	12.98
Toxotes chatareus[4]	UF 241575	See voucher	SRR17680284	438	584,748	1,335.04	1,289	222	2,743	406	13.01
Toxotes jaculatrix[4]	KUI 42174	KUIT 11260	SRR11016332	463	728,311	1,573.03	1,597	551	2,859	446	12.92
Toxotes jaculatrix	ZRC Uncat.	See voucher	SRR17680281	330	395,155	1,197.44	1,138	256	2,388	274	16.05

[1]Previously identified as *Toxotes chatareus*. [2]Previously identified as *Toxotes mekongensis*. [3]Previously identified as *Toxotes siamensis*. [4]Sample used in combined analysis.

Supplementary Table 1 – Molecular voucher information, GenBank SRA accession numbers, and statistics for UCE Loci. All specimens from Charles Darwin University marked as "CDU".
