# Supplementary material for: Phylogenetics of Archerfishes (Toxotidae) and Evolution of the Toxotid Shooting Apparatus
Source: Integr Org Biol. 2022 Mar 21;4(1):obac013. doi: 10.1093/iob/obac013 (PMC9259087; doi:10.1093/iob/obac013)
Supplement: obac013_Supplemental_Files [file obac013_supplemental_files.zip › Supplementary_table_2_rev.pdf]

	Characters									1
	1	1111111112	2222222223	3333333334	4444444445	5555555556	6666666667	7777777778	8888888889	9999999990
	1234567890	1234567890	1234567890	1234567890	1234567890	1234567890	1234567890	1234567890	1234567890	1234567890
Lepomis cyanellus (root)	0-00010000	0-10-01100	1100100010	0000000110	0-00-0-000	0101010100	0101000-10	0000000010	0100100000	000000-110
Lates calcarifer	0-1-000000	1111010001	0100000000	0110011010	0-00-0-000	0100000100	0010000-10	0000000110	0000000000	1010011200
Leptobrama muelleri	0-00100010	1211001011	10-0101-00	1111000100	1011111111	10001010-0	0110110-11	1111011300	1010001110	1110111210
Nematistius pectoralis	1000000000	1000-01001	0110001-10	0010000010	0-00-0-000	?000100110	0011000-00	1100100110	1000000100	000000-300
Perca flavescens	0-01001110	0-00-00110	00-0100000	0000001000	0-00-0-100	00000100-0	0-01000-10	0000000000	0000000000	000000-210
Protoxotes lorentzi	1100111010	1111011111	1111011-01	1101110101	1111110011	00111000--	1110111011	0012001201	-01?000011	1011110100
Toxotes blythii	1101111011	1011001111	1111010011	1101110001	1110011111	1011101101	0100111001	0012000201	-011110011	1102112111
Toxotes carpentariensis	1101111111	1011001111	1111010011	1101110001	1110011111	1011101100	0100111101	0012000201	-011111011	110211*11
Toxotes chatareus	1101111111	1011101111	1111010111	1101110001	1110111111	1011101100	0100111101	0012000201	-011111011	110211*11
Toxotes jaculatrix	1101111111	1111001111	1111010101	1101110001	1010110111	10111000-1	1100111101	0012010301	-011011011	1102112011
Toxotes kimberleyensis	1101111111	1011001111	1111010011	1101010001	1110?11111	10111000-1	0100111101	0012000201	-011111011	1?02111011
Toxotes oligolepis	1101111111	1011001111	1111010101	1101110001	1010111111	1011100101	0100111101	0012010201	-111011011	1112111011
Toxotes sundaicus	1100111111	1011101111	1111010011	1101110001	1110?11111	1011100110	0100111001	0012000201	-011111011	1?02111111

Supplementary Table 2 – Morphological matrix of characters analyzed in the current study (characters 1–100).
Characters coded with an “*” indicate a character coded for multiple states. Characters coded with an “-” indicate a character that was inapplicable for that taxon. Characters coded with an “?” indicate a character that remains uncoded for that taxon. See character descriptions for details.
