# Supplementary material for: Phylogenetics of Archerfishes (Toxotidae) and Evolution of the Toxotid Shooting Apparatus
Source: Integr Org Biol. 2022 Mar 21;4(1):obac013. doi: 10.1093/iob/obac013 (PMC9259087; doi:10.1093/iob/obac013)
Supplement: obac013_Supplemental_Files [file obac013_supplemental_files.zip › Supplementary_table_3.pdf]

Partiton Subset	Best Fitting Model	Number of Sites in Partition	Region(s) in Partition
UCE Partition 1	GTR+G	1,655	UCE 1001 Left Region, UCE 332 Right Region, UCE 408 Right Region, UCE 139 Left Region
UCE Partition 2	GTR+I+G	2,099	UCE 1113 Left Region, UCE 574 Core Region, UCE 509 Left Region, UCE 1001 Core Region
UCE Partition 3	GTR+G	1,372	UCE 1001 Right Region, UCE 383 Core Region, UCE 187 Right Region, UCE 642 Core Region
UCE Partition 4	GTR	97	UCE 1007 Left Region
UCE Partition 5	GTR+G	5,279	UCE 653 Core Region, UCE 1007 Core Region, UCE 1044 Core Region, UCE 600 Core Region, UCE 399 Right Region, UCE 51 Right Region, UCE 915 Core Region, UCE 471 Right Region, UCE 1062 Right Region, UCE 3 Left Region, UCE 435 Core Region, UCE 377 Left Region, UCE 524 Right Region, UCE 181 Left Region
UCE Partition 6	GTR+G	5,781	UCE 410 Left Region, UCE 468 Left Region, UCE 1243 Right Region, UCE 1007 Right Region, UCE 1334 Left Region, UCE 1192 Left Region, UCE 307 Right Region
UCE Partition 7	GTR	843	UCE 335 Right Region, UCE 231 Left Region, UCE 1017 Left Region, UCE 151 Right Region
UCE Partition 8	GTR+G	1,939	UCE 1017 Core Region, UCE 309 Left Region, UCE 570 Left Region, UCE 192 Right Region, UCE 934 Left Region, UCE 376 Left Region, UCE 154 Right Region
UCE Partition 9	GTR+I+G	4,397	UCE 129 Core Region, UCE 143 Right Region, UCE 408 Core Region, UCE 359 Left Region, UCE 1017 Right Region
UCE Partition 10	GTR	115	UCE 1018 Left Region, UCE 162 Left Region
UCE Partition 11	GTR+I+G	2,606	UCE 201 Core Region, UCE 394 Core Region, UCE 1018 Core Region, UCE 716 Right Region
UCE Partition 12	GTR+G	1,818	UCE 116 Right Region, UCE 1046 Left Region, UCE 496 Left Region, UCE 883 Left Region, UCE 1018 Right Region, UCE 541 Left Region, UCE 1260 Left Region
UCE Partition 13	GTR	2,403	UCE 129 Right Region, UCE 88 Right Region, UCE 139 Right Region, UCE 1020 Left Region, UCE 356 Right Region, UCE 493 Right Region, UCE 944 Core Region
UCE Partition 14	GTR+G	2,313	UCE 834 Left Region, UCE 310 Right Region, UCE 1224 Right Region, UCE 1020 Core Region, UCE 1160 Left Region
UCE Partition 15	GTR	3,059	UCE 982 Core Region, UCE 1256 Left Region, UCE 832 Left Region, UCE 526 Right Region, UCE 1020 Right Region, UCE 768 Core Region, UCE 1321 Left Region, UCE 344 Right Region, UCE 597 Left Region, UCE 811 Core Region, UCE 85 Right Region
UCE Partition 16	GTR+G	2,649	UCE 1027 Left Region, UCE 695 Left Region, UCE 331 Core Region, UCE 99 Core Region, UCE 718 Right Region, UCE 737 Right Region
UCE Partition 17	GTR+I+G	2,537	UCE 245 Core Region, UCE 1027 Core Region, UCE 167 Core Region, UCE 472 Left Region, UCE 407 Core Region, UCE 358 Core Region
UCE Partition 18	GTR+G	1,140	UCE 1270 Left Region, UCE 1027 Right Region, UCE 295 Left Region, UCE 351 Core Region, UCE 1160 Right Region
UCE Partition 19	GTR+G	767	UCE 108 Left Region, UCE 1032 Left Region
UCE Partition 20	GTR+I+G	6,186	UCE 476 Left Region, UCE 699 Core Region, UCE 1229 Right Region, UCE 175 Right Region, UCE 289 Left Region, UCE 566 Core Region, UCE 992 Core Region, UCE 3 Core Region, UCE 1032 Core Region
UCE Partition 21	GTR+G	1,817	UCE 1032 Right Region, UCE 143 Left Region, UCE 624 Left Region
UCE Partition 22	GTR	2,262	UCE 1036 Left Region, UCE 122 Right Region, UCE 348 Core Region, UCE 846 Left Region, UCE 149 Core Region, UCE 46 Right Region
UCE Partition 23	GTR+G	2,487	UCE 338 Left Region, UCE 179 Right Region, UCE 1036 Core Region, UCE 1334 Right Region
UCE Partition 24	GTR	2,573	UCE 858 Core Region, UCE 564 Core Region, UCE 1036 Right Region, UCE 227 Core Region, UCE 215 Core Region, UCE 536 Core Region, UCE 815 Core Region, UCE 461 Left Region, UCE 1233 Left Region
UCE Partition 25	GTR+G	3,626	UCE 1038 Left Region, UCE 1234 Core Region, UCE 449 Right Region, UCE 166 Right Region, UCE 792 Core Region
UCE Partition 26	GTR+G	268	UCE 1038 Core Region
UCE Partition 27	GTR+G	348	UCE 1038 Right Region
UCE Partition 28	GTR	3,966	UCE 278 Core Region, UCE 990 Right Region, UCE 834 Right Region, UCE 981 Left Region, UCE 869 Core Region, UCE 1040 Left Region, UCE 220 Right Region, UCE 1173 Core Region, UCE 640 Right Region, UCE 1168 Right Region, UCE 1169 Right Region, UCE 1234 Left Region
UCE Partition 29	GTR+G	2,823	UCE 830 Left Region, UCE 1061 Core Region, UCE 1040 Core Region, UCE 1191 Left Region
UCE Partition 30	GTR+G	2,321	UCE 846 Right Region, UCE 690 Left Region, UCE 1040 Right Region, UCE 374 Core Region, UCE 466 Right Region
UCE Partition 31	GTR+G	2,923	UCE 682 Left Region, UCE 666 Left Region, UCE 3 Right Region, UCE 516 Right Region, UCE 459 Left Region, UCE 305 Right Region, UCE 1042 Left Region, UCE 372 Left Region
UCE Partition 32	GTR+I+G	3,982	UCE 67 Right Region, UCE 203 Right Region, UCE 149 Right Region, UCE 1042 Core Region, UCE 736 Left Region
UCE Partition 33	GTR+G	2,123	UCE 588 Right Region, UCE 1042 Right Region, UCE 1232 Core Region, UCE 167 Right Region, UCE 302 Left Region, UCE 636 Core Region, UCE 471 Left Region
UCE Partition 34	GTR+G	4,415	UCE 201 Right Region, UCE 154 Left Region, UCE 556 Right Region, UCE 167 Left Region, UCE 1044 Left Region, UCE 368 Left Region, UCE 1192 Right Region, UCE 566 Right Region, UCE 620 Left Region, UCE 1279 Right Region, UCE 30 Right Region
UCE Partition 35	GTR+I+G	2,646	UCE 1044 Right Region, UCE 1143 Core Region, UCE 192 Left Region, UCE 450 Core Region, UCE 891 Right Region, UCE 966 Core Region, UCE 705 Right Region, UCE 552 Right Region
UCE Partition 36	GTR+I+G	3,797	UCE 535 Right Region, UCE 802 Right Region, UCE 636 Left Region, UCE 1046 Core Region, UCE 990 Core Region
UCE Partition 37	GTR+G	1,533	UCE 1046 Right Region, UCE 302 Right Region, UCE 392 Right Region, UCE 351 Left Region
UCE Partition 38	GTR+G	3,082	UCE 585 Right Region, UCE 116 Left Region, UCE 245 Right Region, UCE 1048 Left Region
UCE Partition 39	GTR	71	UCE 1048 Core Region
UCE Partition 40	GTR+G	1,103	UCE 1209 Core Region, UCE 1048 Right Region
UCE Partition 41	GTR	3,769	UCE 288 Right Region, UCE 674 Left Region, UCE 271 Core Region, UCE 1175 Left Region, UCE 1053 Left Region, UCE 431 Core Region, UCE 175 Left Region, UCE 605 Right Region, UCE 126 Left Region
UCE Partition 42	GTR+G	465	UCE 1053 Core Region, UCE 348 Right Region
UCE Partition 43	GTR+G	3,423	UCE 532 Right Region, UCE 344 Left Region, UCE 1053 Right Region, UCE 837 Left Region
UCE Partition 44	GTR+G	1,769	UCE 1133 Core Region, UCE 84 Right Region, UCE 1054 Left Region, UCE 288 Core Region
UCE Partition 45	GTR+I+G	122	UCE 1054 Core Region
UCE Partition 46	GTR+G	235	UCE 1054 Right Region, UCE 585 Core Region
UCE Partition 47	GTR+G	2,678	UCE 1056 Left Region, UCE 305 Left Region, UCE 759 Left Region, UCE 696 Left Region, UCE 392 Left Region, UCE 1281 Right Region, UCE 952 Right Region, UCE 731 Core Region
UCE Partition 48	GTR+G	1,718	UCE 1056 Core Region, UCE 191 Core Region
UCE Partition 49	GTR	3,502	UCE 691 Right Region, UCE 1056 Right Region, UCE 655 Left Region, UCE 663 Core Region, UCE 85 Core Region, UCE 139 Core Region, UCE 231 Core Region, UCE 695 Core Region
UCE Partition 50	GTR+G	793	UCE 368 Right Region, UCE 712 Right Region, UCE 106 Left Region
UCE Partition 51	GTR	577	UCE 106 Core Region, UCE 179 Left Region
UCE Partition 52	GTR+G	3,001	UCE 191 Left Region, UCE 106 Right Region, UCE 860 Left Region, UCE 731 Right Region, UCE 47 Left Region, UCE 183 Core Region, UCE 1287 Left Region
UCE Partition 53	GTR	1,887	UCE 74 Core Region, UCE 666 Right Region, UCE 1061 Left Region, UCE 172 Right Region, UCE 924 Core Region, UCE 792 Left Region, UCE 1260 Right Region, UCE 1324 Core Region
UCE Partition 54	GTR	122	UCE 1061 Right Region
UCE Partition 55	GTR+G	120	UCE 442 Right Region, UCE 1062 Left Region
UCE Partition 56	GTR+G	2,516	UCE 833 Left Region, UCE 1062 Core Region, UCE 98 Core Region, UCE 1075 Right Region
UCE Partition 57	GTR	86	UCE 1064 Left Region
UCE Partition 58	GTR+G	986	UCE 1097 Left Region, UCE 1064 Core Region, UCE 151 Core Region, UCE 529 Left Region
UCE Partition 59	GTR+I+G	988	UCE 1064 Right Region
UCE Partition 60	GTR	1,427	UCE 1066 Left Region, UCE 118 Left Region, UCE 376 Right Region, UCE 372 Right Region, UCE 568 Right Region, UCE 943 Left Region, UCE 830 Right Region
UCE Partition 61	GTR+G	1,274	UCE 1211 Left Region, UCE 1106 Left Region, UCE 1251 Right Region, UCE 1066 Core Region
UCE Partition 62	GTR+G	216	UCE 1066 Right Region
UCE Partition 63	GTR+G	595	UCE 1069 Left Region, UCE 795 Left Region
UCE Partition 64	GTR	346	UCE 1238 Left Region, UCE 1069 Core Region
UCE Partition 65	GTR+G	3,922	UCE 47 Right Region, UCE 1069 Right Region, UCE 699 Left Region, UCE 157 Left Region, UCE 564 Left Region, UCE 717 Left Region
UCE Partition 66	GTR+G	847	UCE 1075 Left Region
UCE Partition 67	GTR+G	1,329	UCE 1075 Core Region, UCE 243 Right Region, UCE 319 Left Region
UCE Partition 68	GTR	4,251	UCE 759 Right Region, UCE 26 Core Region, UCE 1184 Core Region, UCE 428 Core Region, UCE 108 Core Region, UCE 99 Left Region, UCE 372 Core Region
UCE Partition 69	GTR+G	3,066	UCE 413 Core Region, UCE 1175 Right Region, UCE 598 Left Region, UCE 108 Right Region, UCE 518 Right Region, UCE 573 Left Region, UCE 536 Left Region
UCE Partition 70	GTR+G	1,501	UCE 1080 Left Region, UCE 30 Left Region
UCE Partition 71	GTR+I+G	1,825	UCE 23 Core Region, UCE 442 Core Region, UCE 1080 Core Region, UCE 685 Core Region

Partiton Subset	Best Fitting Model	Number of Sites in Partition	Region(s) in Partition
UCE Partition 72	GTR+G	4,266	UCE 883 Right Region, UCE 934 Right Region, UCE 435 Right Region, UCE 375 Right Region, UCE 1080 Right Region, UCE 99 Right Region, UCE 183 Left Region, UCE 494 Right Region, UCE 699 Right Region, UCE 837 Right Region, UCE 136 Core Region, UCE 1293 Right Region, UCE 566 Left Region
UCE Partition 73	GTR	530	UCE 736 Core Region, UCE 1087 Left Region, UCE 446 Right Region
UCE Partition 74	GTR+I+G	7,646	UCE 151 Left Region, UCE 535 Left Region, UCE 987 Core Region, UCE 527 Right Region, UCE 524 Left Region, UCE 1326 Left Region, UCE 1087 Core Region, UCE 84 Left Region, UCE 640 Core Region, UCE 1200 Left Region
UCE Partition 75	GTR+G	2,697	UCE 537 Core Region, UCE 115 Right Region, UCE 1087 Right Region, UCE 73 Left Region, UCE 411 Right Region, UCE 242 Left Region
UCE Partition 76	GTR	1,015	UCE 518 Core Region, UCE 109 Left Region, UCE 605 Core Region
UCE Partition 77	GTR	505	UCE 109 Core Region, UCE 546 Core Region
UCE Partition 78	GTR+G	1,291	UCE 109 Right Region, UCE 249 Right Region, UCE 214 Right Region
UCE Partition 79	GTR+I+G	436	UCE 1093 Left Region
UCE Partition 80	GTR+G	3,494	UCE 1093 Core Region, UCE 271 Right Region, UCE 1110 Core Region, UCE 622 Right Region, UCE 495 Left Region
UCE Partition 81	GTR+G	656	UCE 428 Right Region, UCE 1093 Right Region
UCE Partition 82	GTR+G	4,436	UCE 70 Left Region, UCE 154 Core Region, UCE 1097 Core Region, UCE 879 Core Region, UCE 1269 Core Region, UCE 537 Right Region, UCE 690 Core Region, UCE 414 Core Region
UCE Partition 83	GTR+G	1,873	UCE 57 Core Region, UCE 1097 Right Region, UCE 713 Core Region, UCE 971 Left Region
UCE Partition 84	GTR+G	379	UCE 1105 Left Region
UCE Partition 85	GTR+I+G	1,064	UCE 709 Left Region, UCE 287 Core Region, UCE 1154 Core Region, UCE 1105 Core Region
UCE Partition 86	GTR+G	1,513	UCE 546 Left Region, UCE 1105 Right Region, UCE 1154 Right Region
UCE Partition 87	GTR	2,037	UCE 1106 Core Region, UCE 495 Right Region, UCE 697 Right Region, UCE 27 Core Region, UCE 1326 Right Region
UCE Partition 88	GTR+G	1,377	UCE 1106 Right Region
UCE Partition 89	GTR+G	4,509	UCE 864 Right Region, UCE 1108 Left Region, UCE 749 Core Region, UCE 598 Right Region, UCE 238 Core Region, UCE 88 Left Region
UCE Partition 90	GTR	188	UCE 1108 Core Region
UCE Partition 91	GTR+G	371	UCE 1108 Right Region
UCE Partition 92	GTR	597	UCE 408 Left Region, UCE 993 Core Region, UCE 1110 Left Region
UCE Partition 93	GTR+G	2,888	UCE 556 Core Region, UCE 858 Left Region, UCE 1110 Right Region, UCE 830 Core Region, UCE 1242 Core Region, UCE 242 Core Region, UCE 174 Left Region, UCE 1321 Right Region, UCE 396 Right Region, UCE 748 Core Region, UCE 160 Core Region, UCE 468 Core Region
UCE Partition 94	GTR+G	1,961	UCE 245 Left Region, UCE 537 Left Region, UCE 1111 Left Region
UCE Partition 95	GTR	73	UCE 1111 Core Region
UCE Partition 96	GTR+G	1,007	UCE 574 Left Region, UCE 957 Core Region, UCE 1111 Right Region
UCE Partition 97	GTR+G	561	UCE 577 Left Region, UCE 1113 Core Region
UCE Partition 98	GTR+I+G	2,926	UCE 231 Right Region, UCE 1113 Right Region, UCE 493 Left Region, UCE 750 Left Region
UCE Partition 99	GTR+G	1,324	UCE 570 Right Region, UCE 149 Left Region, UCE 1119 Left Region
UCE Partition 100	GTR+G	2,615	UCE 777 Left Region, UCE 1119 Core Region, UCE 792 Right Region, UCE 691 Left Region
UCE Partition 101	GTR+G	650	UCE 377 Right Region, UCE 1119 Right Region, UCE 1167 Left Region
UCE Partition 102	GTR+G	6,132	UCE 51 Core Region, UCE 427 Right Region, UCE 327 Core Region, UCE 498 Left Region, UCE 113 Left Region, UCE 1286 Right Region, UCE 311 Left Region, UCE 159 Left Region, UCE 243 Left Region
UCE Partition 103	GTR	103	UCE 113 Core Region, UCE 569 Core Region
UCE Partition 104	GTR	1,404	UCE 113 Right Region, UCE 734 Right Region, UCE 247 Left Region, UCE 813 Right Region
UCE Partition 105	GTR+G	111	UCE 1133 Left Region, UCE 622 Core Region
UCE Partition 106	GTR	263	UCE 283 Right Region, UCE 1133 Right Region
UCE Partition 107	GTR+G	1,107	UCE 1139 Left Region, UCE 461 Core Region, UCE 295 Right Region
UCE Partition 108	GTR+I+G	5,055	UCE 1256 Core Region, UCE 274 Core Region, UCE 653 Left Region, UCE 1139 Core Region, UCE 267 Core Region, UCE 642 Left Region, UCE 418 Core Region, UCE 570 Core Region
UCE Partition 109	GTR+G	3,477	UCE 1167 Right Region, UCE 1139 Right Region, UCE 338 Right Region, UCE 561 Core Region, UCE 191 Right Region, UCE 1251 Core Region, UCE 1211 Right Region
UCE Partition 110	GTR+G	2,269	UCE 1143 Left Region, UCE 295 Core Region, UCE 1143 Right Region, UCE 363 Right Region, UCE 565 Core Region
UCE Partition 111	GTR+G	5,377	UCE 447 Right Region, UCE 115 Left Region, UCE 70 Core Region, UCE 881 Left Region, UCE 609 Right Region, UCE 214 Left Region
UCE Partition 112	GTR+I+G	2,608	UCE 971 Core Region, UCE 1175 Core Region, UCE 115 Core Region, UCE 577 Core Region
UCE Partition 113	GTR+G	472	UCE 1154 Left Region
UCE Partition 114	GTR+G	2,162	UCE 757 Core Region, UCE 73 Core Region, UCE 116 Core Region, UCE 747 Right Region
UCE Partition 115	GTR+G	1,076	UCE 787 Right Region, UCE 966 Right Region, UCE 1160 Core Region
UCE Partition 116	GTR	318	UCE 1307 Right Region, UCE 1167 Core Region
UCE Partition 117	GTR+G	711	UCE 174 Right Region, UCE 1168 Left Region
UCE Partition 118	GTR+G	2,092	UCE 908 Left Region, UCE 672 Left Region, UCE 826 Left Region, UCE 1168 Core Region, UCE 805 Core Region
UCE Partition 119	GTR+I+G	2,094	UCE 1169 Left Region, UCE 181 Right Region
UCE Partition 120	GTR	304	UCE 282 Left Region, UCE 1169 Core Region
UCE Partition 121	GTR	1,431	UCE 1173 Left Region, UCE 648 Core Region
UCE Partition 122	GTR+G	2,917	UCE 1173 Right Region, UCE 944 Left Region, UCE 449 Left Region
UCE Partition 123	GTR+G	1,814	UCE 444 Core Region, UCE 118 Core Region, UCE 187 Core Region, UCE 368 Core Region
UCE Partition 124	GTR+I+G	50	UCE 118 Right Region
UCE Partition 125	GTR+G	5,301	UCE 1251 Left Region, UCE 921 Right Region, UCE 669 Left Region, UCE 553 Right Region, UCE 24 Right Region, UCE 460 Left Region, UCE 1296 Right Region, UCE 1184 Left Region, UCE 997 Right Region
UCE Partition 126	GTR+G	1,405	UCE 1184 Right Region, UCE 444 Right Region, UCE 196 Core Region, UCE 310 Left Region
UCE Partition 127	GTR+G	3,255	UCE 685 Right Region, UCE 247 Right Region, UCE 1186 Left Region, UCE 201 Left Region, UCE 26 Right Region
UCE Partition 128	GTR+G	56	UCE 1186 Core Region
UCE Partition 129	GTR	1,604	UCE 1263 Left Region, UCE 987 Left Region, UCE 358 Right Region, UCE 879 Left Region, UCE 1186 Right Region, UCE 1200 Right Region
UCE Partition 130	GTR+G	1,601	UCE 759 Core Region, UCE 660 Right Region, UCE 1191 Core Region, UCE 787 Core Region
UCE Partition 131	GTR	702	UCE 750 Core Region, UCE 1191 Right Region, UCE 761 Core Region
UCE Partition 132	GTR+I+G	4,930	UCE 852 Left Region, UCE 837 Core Region, UCE 1192 Core Region, UCE 351 Right Region, UCE 310 Core Region, UCE 411 Core Region, UCE 934 Core Region
UCE Partition 133	GTR+G	1,526	UCE 120 Left Region, UCE 1282 Right Region
UCE Partition 134	GTR	2,289	UCE 120 Core Region, UCE 734 Core Region, UCE 891 Core Region, UCE 660 Core Region, UCE 894 Core Region, UCE 795 Core Region
UCE Partition 135	GTR+G	2,885	UCE 120 Right Region, UCE 414 Left Region, UCE 795 Right Region, UCE 320 Left Region, UCE 1224 Left Region, UCE 706 Right Region
UCE Partition 136	GTR+G	1,364	UCE 971 Right Region, UCE 509 Core Region, UCE 663 Left Region, UCE 54 Right Region, UCE 1200 Core Region
UCE Partition 137	GTR	1,507	UCE 975 Right Region, UCE 1204 Left Region, UCE 585 Left Region
UCE Partition 138	GTR+I+G	3,406	UCE 768 Left Region, UCE 915 Left Region, UCE 573 Core Region, UCE 1204 Core Region, UCE 1263 Core Region
UCE Partition 139	GTR	2,435	UCE 54 Left Region, UCE 855 Right Region, UCE 852 Right Region, UCE 73 Right Region, UCE 1204 Right Region, UCE 76 Left Region, UCE 757 Left Region, UCE 331 Left Region
UCE Partition 140	GTR+G	4,329	UCE 399 Core Region, UCE 383 Left Region, UCE 1209 Left Region, UCE 564 Right Region, UCE 737 Left Region, UCE 367 Right Region, UCE 858 Right Region
UCE Partition 141	GTR	1,596	UCE 203 Left Region, UCE 194 Core Region, UCE 1209 Right Region
UCE Partition 142	GTR+G	3,542	UCE 569 Right Region, UCE 1296 Left Region, UCE 238 Right Region, UCE 348 Left Region, UCE 1211 Core Region, UCE 648 Left Region
UCE Partition 143	GTR+G	4,647	UCE 136 Left Region, UCE 952 Core Region, UCE 1219 Left Region, UCE 311 Right Region, UCE 68 Core Region, UCE 472 Core Region

Partiton Subset	Best Fitting Model	Number of Sites in Partition	Region(s) in Partition
UCE Partition 144	GTR	98	UCE 1219 Core Region
UCE Partition 145	GTR+G	6,696	UCE 427 Left Region, UCE 748 Left Region, UCE 600 Left Region, UCE 1219 Right Region, UCE 863 Right Region, UCE 758 Right Region, UCE 442 Left Region, UCE 435 Left Region
UCE Partition 146	GTR+G	1,441	UCE 469 Left Region, UCE 122 Left Region
UCE Partition 147	GTR+G	824	UCE 122 Core Region, UCE 756 Core Region, UCE 869 Right Region
UCE Partition 148	GTR	978	UCE 1224 Core Region, UCE 835 Core Region
UCE Partition 149	GTR+I+G	3,893	UCE 605 Left Region, UCE 620 Right Region, UCE 1228 Left Region, UCE 808 Core Region, UCE 321 Left Region
UCE Partition 150	GTR	1,281	UCE 24 Core Region, UCE 705 Left Region, UCE 1228 Core Region, UCE 504 Core Region, UCE 568 Core Region, UCE 836 Core Region
UCE Partition 151	GTR	169	UCE 1228 Right Region
UCE Partition 152	GTR	1,118	UCE 157 Core Region, UCE 643 Left Region, UCE 1229 Left Region
UCE Partition 153	GTR+G	209	UCE 1229 Core Region
UCE Partition 154	GTR+G	557	UCE 1232 Left Region, UCE 88 Core Region
UCE Partition 155	GTR+G	1,783	UCE 1232 Right Region, UCE 74 Right Region
UCE Partition 156	GTR+G	1,501	UCE 1233 Core Region, UCE 811 Right Region, UCE 67 Left Region, UCE 885 Right Region
UCE Partition 157	GTR+G	425	UCE 1233 Right Region
UCE Partition 158	GTR	1,116	UCE 1234 Right Region, UCE 474 Core Region
UCE Partition 159	GTR	320	UCE 27 Left Region, UCE 1238 Core Region
UCE Partition 160	GTR+G	2,662	UCE 701 Core Region, UCE 1238 Right Region, UCE 468 Right Region
UCE Partition 161	GTR+G	2,441	UCE 124 Left Region, UCE 649 Left Region, UCE 891 Left Region, UCE 461 Right Region
UCE Partition 162	GTR+I+G	5,029	UCE 1338 Left Region, UCE 46 Core Region, UCE 666 Core Region, UCE 968 Core Region, UCE 705 Core Region, UCE 124 Core Region, UCE 706 Core Region
UCE Partition 163	GTR+G	757	UCE 124 Right Region, UCE 287 Right Region
UCE Partition 164	GTR+G	1,667	UCE 402 Right Region, UCE 1242 Left Region, UCE 356 Left Region
UCE Partition 165	GTR+G	2,551	UCE 851 Left Region, UCE 76 Core Region, UCE 797 Right Region, UCE 1242 Right Region, UCE 466 Core Region
UCE Partition 166	GTR+G	1,700	UCE 636 Right Region, UCE 813 Core Region, UCE 1243 Left Region
UCE Partition 167	GTR+G	74	UCE 1243 Core Region
UCE Partition 168	GTR	210	UCE 1249 Left Region, UCE 26 Left Region
UCE Partition 169	GTR+G	644	UCE 458 Right Region, UCE 1249 Core Region, UCE 851 Right Region
UCE Partition 170	GTR+I+G	1,029	UCE 1249 Right Region
UCE Partition 171	GTR+G	947	UCE 1253 Left Region
UCE Partition 172	GTR	124	UCE 416 Core Region, UCE 1253 Core Region
UCE Partition 173	GTR	2,124	UCE 556 Left Region, UCE 567 Left Region, UCE 140 Right Region, UCE 1253 Right Region, UCE 319 Right Region, UCE 1277 Left Region, UCE 166 Left Region
UCE Partition 174	GTR+G	1,071	UCE 61 Right Region, UCE 1256 Right Region, UCE 170 Core Region, UCE 251 Right Region
UCE Partition 175	GTR+I+G	974	UCE 126 Core Region
UCE Partition 176	GTR+G	1,364	UCE 126 Right Region, UCE 808 Right Region, UCE 525 Right Region, UCE 161 Left Region
UCE Partition 177	GTR+G	3,626	UCE 810 Core Region, UCE 924 Left Region, UCE 975 Left Region, UCE 1260 Core Region, UCE 1277 Core Region, UCE 525 Core Region
UCE Partition 178	GTR+I+G	471	UCE 1263 Right Region
UCE Partition 179	GTR+G	2,580	UCE 1269 Left Region, UCE 1338 Right Region, UCE 802 Left Region, UCE 162 Right Region, UCE 993 Right Region, UCE 717 Right Region
UCE Partition 180	GTR+G	396	UCE 474 Left Region, UCE 642 Right Region, UCE 1269 Right Region
UCE Partition 181	GTR+G	4,006	UCE 665 Right Region, UCE 374 Right Region, UCE 1270 Core Region, UCE 185 Core Region, UCE 494 Core Region, UCE 727 Left Region
UCE Partition 182	GTR	2,509	UCE 987 Right Region, UCE 835 Left Region, UCE 327 Left Region, UCE 143 Core Region, UCE 418 Right Region, UCE 1270 Right Region, UCE 975 Core Region, UCE 399 Left Region, UCE 968 Right Region
UCE Partition 183	GTR+G	369	UCE 1273 Left Region
UCE Partition 184	GTR+G	2,765	UCE 649 Core Region, UCE 1273 Core Region, UCE 815 Left Region, UCE 861 Left Region
UCE Partition 185	GTR+G	1,025	UCE 1273 Right Region, UCE 287 Left Region, UCE 526 Left Region
UCE Partition 186	GTR+G	3,401	UCE 692 Left Region, UCE 1276 Left Region, UCE 885 Left Region, UCE 1324 Right Region, UCE 541 Right Region
UCE Partition 187	GTR+G	50	UCE 1276 Core Region
UCE Partition 188	GTR+G	4,161	UCE 568 Left Region, UCE 420 Left Region, UCE 1276 Right Region, UCE 358 Left Region, UCE 952 Left Region, UCE 894 Left Region, UCE 152 Left Region, UCE 283 Left Region
UCE Partition 189	GTR+G	237	UCE 1277 Right Region
UCE Partition 190	GTR+G	830	UCE 1279 Left Region, UCE 170 Left Region, UCE 359 Right Region
UCE Partition 191	GTR+G	5,040	UCE 376 Core Region, UCE 1279 Core Region, UCE 170 Right Region, UCE 332 Core Region, UCE 392 Core Region, UCE 697 Core Region, UCE 1316 Core Region, UCE 718 Core Region
UCE Partition 192	GTR+G	3,181	UCE 660 Left Region, UCE 1281 Left Region, UCE 545 Right Region, UCE 249 Core Region, UCE 547 Right Region
UCE Partition 193	GTR+I+G	1,882	UCE 997 Core Region, UCE 283 Core Region, UCE 692 Core Region, UCE 1281 Core Region
UCE Partition 194	GTR	1,851	UCE 921 Left Region, UCE 567 Core Region, UCE 1282 Left Region, UCE 600 Right Region
UCE Partition 195	GTR+G	707	UCE 669 Core Region, UCE 1282 Core Region
UCE Partition 196	GTR+G	623	UCE 983 Right Region, UCE 68 Left Region, UCE 1286 Left Region
UCE Partition 197	GTR+G	1,282	UCE 802 Core Region, UCE 450 Left Region, UCE 142 Right Region, UCE 879 Right Region, UCE 1286 Core Region
UCE Partition 198	GTR+G	1,606	UCE 552 Core Region, UCE 1287 Core Region, UCE 948 Core Region
UCE Partition 199	GTR	1,511	UCE 643 Right Region, UCE 1287 Right Region
UCE Partition 200	GTR+G	385	UCE 129 Left Region, UCE 307 Core Region, UCE 663 Right Region
UCE Partition 201	GTR+G	2,590	UCE 516 Left Region, UCE 932 Left Region, UCE 1293 Left Region
UCE Partition 202	GTR	55	UCE 1293 Core Region
UCE Partition 203	GTR+G	82	UCE 1295 Left Region
UCE Partition 204	GTR+I+G	1,688	UCE 160 Left Region, UCE 1324 Left Region, UCE 1295 Core Region
UCE Partition 205	GTR+G	848	UCE 1295 Right Region
UCE Partition 206	GTR+G	242	UCE 1296 Core Region, UCE 74 Left Region, UCE 516 Core Region
UCE Partition 207	GTR	1,782	UCE 346 Core Region, UCE 359 Core Region, UCE 204 Left Region, UCE 13 Left Region, UCE 553 Core Region
UCE Partition 208	GTR	278	UCE 13 Core Region
UCE Partition 209	GTR+I+G	2,287	UCE 215 Left Region, UCE 836 Left Region, UCE 13 Right Region
UCE Partition 210	GTR+G	4,100	UCE 157 Right Region, UCE 870 Right Region, UCE 410 Right Region, UCE 21 Right Region, UCE 1305 Left Region, UCE 353 Right Region
UCE Partition 211	GTR	102	UCE 1305 Core Region
UCE Partition 212	GTR+G	6,002	UCE 540 Left Region, UCE 458 Core Region, UCE 526 Core Region, UCE 1305 Right Region, UCE 238 Left Region, UCE 220 Core Region, UCE 561 Left Region
UCE Partition 213	GTR+G	809	UCE 1307 Left Region
UCE Partition 214	GTR+G	232	UCE 1307 Core Region
UCE Partition 215	GTR+G	931	UCE 1316 Left Region, UCE 418 Left Region, UCE 932 Right Region
UCE Partition 216	GTR	1,467	UCE 431 Right Region, UCE 159 Core Region, UCE 545 Core Region, UCE 592 Left Region, UCE 1316 Right Region
UCE Partition 217	GTR+I+G	3,472	UCE 1318 Left Region, UCE 227 Left Region, UCE 305 Core Region, UCE 204 Right Region, UCE 647 Left Region

Partiton Subset	Best Fitting Model	Number of Sites in Partition	Region(s) in Partition
UCE Partition 218	GTR+G	50	UCE 1318 Core Region
UCE Partition 219	GTR+G	2,991	UCE 251 Core Region, UCE 1318 Right Region, UCE 924 Right Region, UCE 761 Left Region
UCE Partition 220	GTR+I+G	2,075	UCE 693 Core Region, UCE 335 Left Region, UCE 1321 Core Region
UCE Partition 221	GTR	164	UCE 204 Core Region, UCE 1326 Core Region
UCE Partition 222	GTR	57	UCE 1334 Core Region
UCE Partition 223	GTR	2,306	UCE 331 Right Region, UCE 870 Core Region, UCE 762 Left Region, UCE 1338 Core Region, UCE 819 Right Region, UCE 980 Right Region, UCE 472 Right Region, UCE 833 Core Region
UCE Partition 224	GTR+G	4,122	UCE 624 Right Region, UCE 665 Left Region, UCE 25 Left Region, UCE 136 Right Region, UCE 252 Left Region, UCE 748 Right Region, UCE 819 Core Region
UCE Partition 225	GTR	313	UCE 427 Core Region, UCE 140 Left Region
UCE Partition 226	GTR+I+G	5,181	UCE 161 Core Region, UCE 540 Right Region, UCE 34 Left Region, UCE 982 Right Region, UCE 140 Core Region, UCE 420 Right Region, UCE 383 Right Region
UCE Partition 227	GTR	485	UCE 808 Left Region, UCE 142 Left Region, UCE 701 Right Region
UCE Partition 228	GTR+G	805	UCE 142 Core Region
UCE Partition 229	GTR	145	UCE 152 Core Region
UCE Partition 230	GTR+G	1,584	UCE 152 Right Region, UCE 609 Left Region
UCE Partition 231	GTR+G	2,580	UCE 159 Right Region, UCE 549 Right Region, UCE 567 Right Region, UCE 607 Right Region
UCE Partition 232	GTR+G	1,067	UCE 160 Right Region
UCE Partition 233	GTR+G	1,456	UCE 810 Left Region, UCE 320 Right Region, UCE 161 Right Region, UCE 565 Right Region
UCE Partition 234	GTR+G	2,997	UCE 38 Core Region, UCE 943 Right Region, UCE 549 Left Region, UCE 162 Core Region
UCE Partition 235	GTR+G	834	UCE 166 Core Region, UCE 580 Left Region, UCE 527 Core Region
UCE Partition 236	GTR+G	3,193	UCE 466 Left Region, UCE 172 Left Region, UCE 981 Right Region, UCE 574 Right Region, UCE 474 Right Region, UCE 672 Right Region
UCE Partition 237	GTR+I+G	1,304	UCE 469 Right Region, UCE 749 Right Region, UCE 172 Core Region
UCE Partition 238	GTR+G	1,324	UCE 498 Right Region, UCE 176 Core Region, UCE 174 Core Region
UCE Partition 239	GTR+G	2,720	UCE 249 Left Region, UCE 394 Right Region, UCE 761 Right Region, UCE 836 Right Region, UCE 61 Left Region, UCE 685 Left Region, UCE 494 Left Region, UCE 175 Core Region, UCE 861 Core Region
UCE Partition 240	GTR+G	1,501	UCE 176 Left Region, UCE 713 Left Region, UCE 540 Core Region, UCE 813 Left Region, UCE 215 Right Region
UCE Partition 241	GTR	184	UCE 176 Right Region
UCE Partition 242	GTR	544	UCE 179 Core Region
UCE Partition 243	GTR	180	UCE 181 Core Region
UCE Partition 244	GTR+I+G	2,203	UCE 459 Core Region, UCE 183 Right Region, UCE 966 Left Region
UCE Partition 245	GTR+G	344	UCE 185 Left Region
UCE Partition 246	GTR+G	301	UCE 185 Right Region
UCE Partition 247	GTR+G	712	UCE 252 Core Region, UCE 187 Left Region
UCE Partition 248	GTR	1,690	UCE 192 Core Region, UCE 717 Core Region, UCE 420 Core Region
UCE Partition 249	GTR+G	1,721	UCE 194 Left Region, UCE 444 Left Region, UCE 747 Core Region, UCE 846 Core Region
UCE Partition 250	GTR+G	1,021	UCE 194 Right Region, UCE 527 Left Region
UCE Partition 251	GTR+G	50	UCE 196 Left Region
UCE Partition 252	GTR+G	2,019	UCE 196 Right Region, UCE 21 Left Region
UCE Partition 253	GTR	1,434	UCE 536 Right Region, UCE 982 Left Region, UCE 750 Right Region, UCE 274 Left Region, UCE 199 Left Region, UCE 572 Left Region, UCE 718 Left Region
UCE Partition 254	GTR+G	2,613	UCE 199 Core Region, UCE 31 Core Region, UCE 737 Core Region, UCE 289 Right Region, UCE 320 Core Region
UCE Partition 255	GTR+G	3,327	UCE 510 Right Region, UCE 227 Right Region, UCE 199 Right Region, UCE 655 Right Region
UCE Partition 256	GTR	292	UCE 203 Core Region
UCE Partition 257	GTR+G	367	UCE 498 Core Region, UCE 21 Core Region
UCE Partition 258	GTR+G	1,597	UCE 826 Core Region, UCE 870 Left Region, UCE 695 Right Region, UCE 609 Core Region, UCE 214 Core Region
UCE Partition 259	GTR+G	2,282	UCE 957 Left Region, UCE 860 Right Region, UCE 219 Left Region, UCE 851 Core Region
UCE Partition 260	GTR	1,582	UCE 219 Core Region, UCE 777 Core Region, UCE 653 Right Region, UCE 25 Core Region, UCE 493 Core Region
UCE Partition 261	GTR	1,447	UCE 524 Core Region, UCE 219 Right Region, UCE 547 Core Region
UCE Partition 262	GTR	1,332	UCE 810 Right Region, UCE 220 Left Region, UCE 992 Right Region
UCE Partition 263	GTR+G	808	UCE 23 Left Region, UCE 697 Left Region
UCE Partition 264	GTR+G	1,474	UCE 23 Right Region, UCE 276 Right Region, UCE 34 Core Region
UCE Partition 265	GTR	953	UCE 24 Left Region, UCE 762 Right Region
UCE Partition 266	GTR+G	364	UCE 242 Right Region
UCE Partition 267	GTR+G	103	UCE 549 Core Region, UCE 243 Core Region
UCE Partition 268	GTR+G	2,948	UCE 247 Core Region, UCE 805 Right Region, UCE 709 Right Region, UCE 749 Left Region
UCE Partition 269	GTR+G	938	UCE 25 Right Region
UCE Partition 270	GTR+G	91	UCE 251 Left Region
UCE Partition 271	GTR	530	UCE 651 Right Region, UCE 252 Right Region, UCE 607 Left Region
UCE Partition 272	GTR+I+G	887	UCE 456 Core Region, UCE 852 Core Region, UCE 267 Left Region, UCE 993 Left Region
UCE Partition 273	GTR+G	1,231	UCE 620 Core Region, UCE 271 Left Region, UCE 57 Left Region, UCE 267 Right Region
UCE Partition 274	GTR+G	2,616	UCE 532 Core Region, UCE 27 Right Region, UCE 278 Left Region, UCE 476 Right Region, UCE 768 Right Region
UCE Partition 275	GTR+G	3,447	UCE 98 Right Region, UCE 60 Left Region, UCE 647 Core Region, UCE 510 Left Region, UCE 274 Right Region, UCE 446 Left Region
UCE Partition 276	GTR	547	UCE 276 Left Region, UCE 460 Core Region
UCE Partition 277	GTR+I+G	522	UCE 276 Core Region
UCE Partition 278	GTR	817	UCE 834 Core Region, UCE 777 Right Region, UCE 278 Right Region
UCE Partition 279	GTR	735	UCE 881 Right Region, UCE 68 Right Region, UCE 280 Left Region
UCE Partition 280	GTR+G	1,771	UCE 545 Left Region, UCE 280 Core Region
UCE Partition 281	GTR	584	UCE 280 Right Region
UCE Partition 282	GTR+G	477	UCE 282 Core Region
UCE Partition 283	GTR+G	2,160	UCE 282 Right Region, UCE 377 Core Region
UCE Partition 284	GTR+G	1,995	UCE 908 Right Region, UCE 321 Core Region, UCE 67 Core Region, UCE 864 Left Region, UCE 288 Left Region, UCE 64 Right Region, UCE 410 Core Region, UCE 671 Left Region
UCE Partition 285	GTR+G	54	UCE 289 Core Region
UCE Partition 286	GTR+G	567	UCE 30 Core Region
UCE Partition 287	GTR+I+G	2,582	UCE 302 Core Region, UCE 309 Core Region, UCE 54 Core Region, UCE 541 Core Region
UCE Partition 288	GTR+G	1,891	UCE 690 Right Region, UCE 983 Left Region, UCE 307 Left Region, UCE 31 Right Region
UCE Partition 289	GTR+G	933	UCE 309 Right Region, UCE 762 Core Region
UCE Partition 290	GTR	352	UCE 31 Left Region
UCE Partition 291	GTR	51	UCE 311 Core Region

Partiton Subset	Best Fitting Model	Number of Sites in Partition	Region(s) in Partition
UCE Partition 292	GTR	64	UCE 319 Core Region
UCE Partition 293	GTR+I+G	2,649	UCE 321 Right Region, UCE 682 Right Region, UCE 712 Left Region
UCE Partition 294	GTR+G	532	UCE 327 Right Region, UCE 46 Left Region
UCE Partition 295	GTR	1,322	UCE 458 Left Region, UCE 332 Left Region, UCE 416 Left Region
UCE Partition 296	GTR+G	805	UCE 335 Core Region
UCE Partition 297	GTR+I+G	558	UCE 338 Core Region
UCE Partition 298	GTR+G	367	UCE 34 Right Region, UCE 665 Core Region
UCE Partition 299	GTR	793	UCE 344 Core Region, UCE 960 Left Region, UCE 693 Right Region
UCE Partition 300	GTR+G	151	UCE 346 Left Region
UCE Partition 301	GTR+I+G	1,851	UCE 504 Right Region, UCE 346 Right Region
UCE Partition 302	GTR+G	2,389	UCE 960 Right Region, UCE 353 Left Region, UCE 450 Right Region, UCE 572 Core Region, UCE 758 Left Region
UCE Partition 303	GTR	57	UCE 353 Core Region
UCE Partition 304	GTR+G	667	UCE 734 Left Region, UCE 356 Core Region
UCE Partition 305	GTR+G	61	UCE 363 Left Region
UCE Partition 306	GTR+G	1,986	UCE 61 Core Region, UCE 713 Right Region, UCE 363 Core Region
UCE Partition 307	GTR+G	970	UCE 367 Left Region, UCE 38 Right Region, UCE 535 Core Region, UCE 504 Left Region, UCE 948 Left Region
UCE Partition 308	GTR	550	UCE 367 Core Region, UCE 932 Core Region
UCE Partition 309	GTR+G	1,834	UCE 431 Left Region, UCE 411 Left Region, UCE 374 Left Region
UCE Partition 310	GTR+I+G	351	UCE 375 Left Region
UCE Partition 311	GTR	1,159	UCE 855 Core Region, UCE 375 Core Region
UCE Partition 312	GTR+G	679	UCE 38 Left Region
UCE Partition 313	GTR+G	184	UCE 394 Left Region
UCE Partition 314	GTR	1,493	UCE 396 Left Region, UCE 552 Left Region, UCE 990 Left Region
UCE Partition 315	GTR+G	680	UCE 396 Core Region
UCE Partition 316	GTR+G	1,261	UCE 402 Left Region, UCE 525 Left Region
UCE Partition 317	GTR	316	UCE 669 Right Region, UCE 402 Core Region
UCE Partition 318	GTR+G	614	UCE 968 Left Region, UCE 407 Left Region
UCE Partition 319	GTR+G	1,334	UCE 407 Right Region, UCE 835 Right Region
UCE Partition 320	GTR+G	1,308	UCE 532 Left Region, UCE 546 Right Region, UCE 98 Left Region, UCE 413 Left Region
UCE Partition 321	GTR+G	2,803	UCE 471 Core Region, UCE 883 Core Region, UCE 832 Right Region, UCE 413 Right Region
UCE Partition 322	GTR+G	707	UCE 588 Left Region, UCE 414 Right Region
UCE Partition 323	GTR+G	1,837	UCE 957 Right Region, UCE 416 Right Region
UCE Partition 324	GTR+G	78	UCE 428 Left Region
UCE Partition 325	GTR+G	1,450	UCE 446 Core Region, UCE 908 Core Region
UCE Partition 326	GTR+G	2,563	UCE 915 Right Region, UCE 580 Core Region, UCE 447 Left Region
UCE Partition 327	GTR	1,711	UCE 597 Right Region, UCE 447 Core Region, UCE 980 Left Region
UCE Partition 328	GTR	257	UCE 449 Core Region, UCE 57 Right Region
UCE Partition 329	GTR+G	1,533	UCE 869 Left Region, UCE 456 Right Region, UCE 456 Left Region
UCE Partition 330	GTR+G	140	UCE 692 Right Region, UCE 459 Right Region
UCE Partition 331	GTR+G	2,205	UCE 671 Right Region, UCE 944 Right Region, UCE 460 Right Region
UCE Partition 332	GTR	52	UCE 469 Core Region
UCE Partition 333	GTR	273	UCE 47 Core Region
UCE Partition 334	GTR+G	113	UCE 476 Core Region
UCE Partition 335	GTR	143	UCE 495 Core Region
UCE Partition 336	GTR+G	442	UCE 496 Core Region
UCE Partition 337	GTR+G	409	UCE 496 Right Region
UCE Partition 338	GTR+G	1,051	UCE 649 Right Region, UCE 668 Left Region, UCE 503 Left Region
UCE Partition 339	GTR+G	297	UCE 592 Core Region, UCE 503 Core Region
UCE Partition 340	GTR+G	1,462	UCE 706 Left Region, UCE 503 Right Region
UCE Partition 341	GTR+I+G	1,606	UCE 672 Core Region, UCE 509 Right Region
UCE Partition 342	GTR+I+G	283	UCE 51 Left Region
UCE Partition 343	GTR	382	UCE 510 Core Region
UCE Partition 344	GTR+G	1,086	UCE 518 Left Region
UCE Partition 345	GTR+G	1,892	UCE 529 Core Region, UCE 863 Left Region, UCE 833 Right Region, UCE 811 Left Region
UCE Partition 346	GTR+G	288	UCE 529 Right Region
UCE Partition 347	GTR	173	UCE 547 Left Region, UCE 624 Core Region
UCE Partition 348	GTR+G	1,336	UCE 592 Right Region, UCE 647 Right Region, UCE 553 Left Region
UCE Partition 349	GTR	990	UCE 861 Right Region, UCE 607 Core Region, UCE 561 Right Region
UCE Partition 350	GTR+G	529	UCE 819 Left Region, UCE 565 Left Region, UCE 716 Core Region
UCE Partition 351	GTR+G	1,720	UCE 569 Left Region, UCE 797 Left Region
UCE Partition 352	GTR	983	UCE 960 Core Region, UCE 572 Right Region
UCE Partition 353	GTR+G	266	UCE 573 Right Region
UCE Partition 354	GTR+G	523	UCE 577 Right Region
UCE Partition 355	GTR	328	UCE 701 Left Region, UCE 580 Right Region
UCE Partition 356	GTR	491	UCE 588 Core Region
UCE Partition 357	GTR+G	3,378	UCE 597 Core Region, UCE 70 Right Region, UCE 85 Left Region, UCE 716 Left Region
UCE Partition 358	GTR+G	124	UCE 598 Core Region
UCE Partition 359	GTR	51	UCE 60 Core Region
UCE Partition 360	GTR+G	419	UCE 60 Right Region
UCE Partition 361	GTR+G	1,104	UCE 756 Right Region, UCE 622 Left Region
UCE Partition 362	GTR+G	768	UCE 64 Left Region, UCE 992 Left Region
UCE Partition 363	GTR+G	903	UCE 64 Core Region
UCE Partition 364	GTR+I+G	150	UCE 640 Left Region
UCE Partition 365	GTR	81	UCE 643 Core Region

Partiton Subset	Best Fitting Model	Number of Sites in Partition	Region(s) in Partition
UCE Partition 366	GTR	788	UCE 648 Right Region
UCE Partition 367	GTR+G	62	UCE 651 Left Region
UCE Partition 368	GTR+I+G	1,252	UCE 651 Core Region
UCE Partition 369	GTR+G	105	UCE 655 Core Region
UCE Partition 370	GTR+G	1,063	UCE 674 Right Region, UCE 668 Core Region
UCE Partition 371	GTR	1,145	UCE 668 Right Region, UCE 781 Right Region
UCE Partition 372	GTR	50	UCE 671 Core Region
UCE Partition 373	GTR	51	UCE 674 Core Region
UCE Partition 374	GTR+G	128	UCE 682 Core Region, UCE 757 Right Region
UCE Partition 375	GTR+G	74	UCE 691 Core Region
UCE Partition 376	GTR+G	590	UCE 693 Left Region
UCE Partition 377	GTR	51	UCE 696 Core Region
UCE Partition 378	GTR+G	1,047	UCE 696 Right Region
UCE Partition 379	GTR+I+G	342	UCE 709 Core Region
UCE Partition 380	GTR+G	399	UCE 712 Core Region, UCE 832 Core Region
UCE Partition 381	GTR	211	UCE 727 Core Region, UCE 885 Core Region
UCE Partition 382	GTR	726	UCE 747 Left Region, UCE 727 Right Region
UCE Partition 383	GTR+G	1,548	UCE 731 Left Region, UCE 787 Left Region
UCE Partition 384	GTR	565	UCE 736 Right Region
UCE Partition 385	GTR+G	1,015	UCE 756 Left Region
UCE Partition 386	GTR	64	UCE 758 Core Region
UCE Partition 387	GTR	59	UCE 76 Right Region
UCE Partition 388	GTR+G	1,362	UCE 781 Left Region
UCE Partition 389	GTR+G	92	UCE 781 Core Region
UCE Partition 390	GTR	68	UCE 797 Core Region
UCE Partition 391	GTR+I+G	338	UCE 805 Left Region
UCE Partition 392	GTR	62	UCE 815 Right Region
UCE Partition 393	GTR+G	275	UCE 826 Right Region
UCE Partition 394	GTR	52	UCE 84 Core Region
UCE Partition 395	GTR+G	329	UCE 855 Left Region
UCE Partition 396	GTR+G	54	UCE 860 Core Region
UCE Partition 397	GTR+G	163	UCE 863 Core Region
UCE Partition 398	GTR	50	UCE 864 Core Region
UCE Partition 399	GTR	57	UCE 881 Core Region
UCE Partition 400	GTR+I+G	1,276	UCE 89 Left Region
UCE Partition 401	GTR+G	293	UCE 89 Core Region
UCE Partition 402	GTR+G	370	UCE 89 Right Region
UCE Partition 403	GTR+G	623	UCE 997 Left Region, UCE 894 Right Region
UCE Partition 404	GTR+G	114	UCE 921 Core Region
UCE Partition 405	GTR	195	UCE 943 Core Region
UCE Partition 406	GTR	295	UCE 948 Right Region
UCE Partition 407	GTR+G	1,139	UCE 980 Core Region
UCE Partition 408	GTR+G	393	UCE 981 Core Region
UCE Partition 409	GTR+G	56	UCE 983 Core Region
